# Coding quality of deaths and its impact on elderly unintentional fall mortality data from 1990 to 2019: a retrospective analysis of the WHO Mortality Database

**DOI:** 10.1186/s12877-021-02744-3

**Published:** 2022-01-24

**Authors:** Junjie Hua, Peishan Ning, Peixia Cheng, Zhenzhen Rao, Jieyi He, Wangxin Xiao, Li Li, Yanhong Fu, Ruotong Li, Jie Li, Wanhui Wang, David C. Schwebel, Guoqing Hu

**Affiliations:** 1 Department of Epidemiology and Health Statistics; Hunan Provincial Key Laboratory of Clinical Epidemiology, Xiangya School of Public Health; National Clinical Research Center for Geriatric Disorders, Xiangya Hospital, Central South University, Changsha, 410078 China; 2 Department of Psychology, University of Alabama at Birmingham, Birmingham, AL 35294 USA

**Keywords:** Unintentional fall, Mortality, Data availability, Coding quality, WHO Mortality Database

## Abstract

**Background:**

Several studies have assessed the reporting quality of all-cause mortality data from the WHO Mortality Database, but little is known about coding quality and its impact on elderly unintentional fall mortality data worldwide. We aimed to assess the coding quality of deaths and its impact on elderly unintentional fall mortality.

**Methods:**

Using data from the WHO Mortality Database, 1990–2019, we calculated the number of countries/territories that had mortality data in the database, and the proportion of deaths with five types of problematic codes based on the 10th International Classification of Disease (unspecified deaths, injury deaths with undetermined intent, unspecified unintentional injury, unintentional falls with unspecified mechanism, unintentional falls with unknown occurrence place). We estimated age-adjusted unintentional fall mortality before and after correcting problematic codes.

**Results:**

Only 64% (124/194) of WHO member states had at least 1 year of mortality data in the database during 1990–2019, and data unavailability was more common for underdeveloped countries/territories than for developed countries/territories. Coding quality was poor for many countries/territories. Among the study years when countries/territories possessed mortality data, 80, 53, 51, and 63% had a proportion of unintentional fall deaths with unspecified mechanism over 50% in low-income, lower middle-income, upper middle-income, and high-income countries/territories, respectively; comparable proportions for unintentional fall deaths with unknown occurrence place were 100, 42, 71, and 62%. Among the 94 countries/territories having mortality data, problematic codes caused a relative mortality difference ≥ 50% in 59 countries/territories (63%). After correcting problematic codes, 5 of 55 countries/territories with data witnessed a reverse in mortality changes between 2005 and 2015. Among the 82 countries/territories with mortality data for 5 or more years, 18 countries/territories (22%) experienced a directional reverse in linear regression coefficient.

**Conclusions:**

The availability and coding quality of global data related to elderly unintentional fall mortality was poor between 1990 and 2019. When data are available, varying coding quality across countries/territories and over time have a substantial impact on mortality estimates and mortality comparisons. Global agencies plus each individual government should be aware of the importance of collecting and sharing high-quality mortality data, and take action to improve data quality for inclusion in the WHO Mortality Database.

**Supplementary Information:**

The online version contains supplementary material available at 10.1186/s12877-021-02744-3.

## Introduction

High-quality death data form a base to develop health policies, conduct health research, and make health-related decisions [1]. Mortality has been selected as the indicator to assess progress in several targets of the Sustainable Development Goals (SDGs), such as maternal mortality (SDG 3.1), newborn and child mortality (SDG 3.2), and road traffic injuries (SDG 3.6) [2]. The World Health Organization (WHO) requests its member states to report basic death data to the WHO annually and compiles these data into the WHO Mortality Database [3].

Currently, the WHO Mortality Database is the only open-access mortality data source that details deaths from specific International Classification of Disease (ICD) codes in the world. WHO mortality data have been frequently used by other international organizations like World Bank, the Global Burden of Disease (GBD) study group, and health researchers [4–7]. It is concerning, therefore, that data users may not realize that data quality from many low- and middle-income countries in the WHO Mortality Database is not as high as those from high-income countries [3, 8].

A limited number of previous studies have assessed the reporting quality of all-cause mortality data from the WHO Mortality Database [5, 8–10]. Although valuable, the results of these studies are difficult to compare because they use different quality indicators and examine data across different study time periods. In addition, previous studies relied on general quality indicators to assess the reporting quality of all-cause mortality data, inadequately reflecting the reporting quality of specific causes of death relevant to policy-making like unintentional falls among old adults.

Elderly falls have been a leading cause of global unintentional injury deaths for many years [11], and as the population gets older, elderly falls have become an increasingly important public health challenge worldwide [12]. Two publications adopted the WHO mortality data to examine changes in fall-related mortality from 2010 to 2014 in the United States [13] and between 2006 and 2016 in Australia and the United Kingdom [14], but neither assessed the reporting quality of fall-related mortality data and its impact on the results. Three studies evaluating the reporting quality of fall mortality in the United States suggested that the recent reporting quality of mortality data has improved substantially and therefore created an artificial impression of potential impact of sharply increasing rates of elderly unintentional fall mortality [15–17]. Research evidence, however, is absent for all other countries and territories included in the WHO Mortality Database.

Using a set of unified quality indicators, we assessed the reporting quality of elderly fall deaths for all countries and territories included in the WHO Mortality Database and its impact on elderly fall mortality rates from 1990 to 2019.

## Methods

### Data source

Data were obtained from the WHO Mortality Database, which collects the number of deaths by country/territory, year, cause of death, sex, and age group since 1950 and allows free online access to the aggregated data online. Four versions (7th to 10th revisions) of the International Classification of Disease (ICD) were used to code the deaths by different countries across different time periods [3]. The WHO Mortality Database is updated regularly, with the update used in this manuscript occurring in March 2021.

Population data for each country/territory were derived from the estimates and projections of United Nations (UN) World Population Prospects 2019 (WPP 2019). The population by country/territory, year, sex, and age is available from 1950 to 2100 [18].

### Quality measures of mortality data

Because limited data were available in the WHO Mortality Database, we used two types of indicators to assess the quality of unintentional fall mortality data; these indicators were selected based on previously published literature [8–10].

- *Data availability*: We used the presence of mortality data in the WHO Mortality Database to reflect data availability for free access from each country/territory and year. Since most countries/territories started to use the ICD-10 codes to classify deaths in the 1990s, we chose the time period of 1990–2019 for this study’s focus. We divided the study time period of 1990–2019 into seven groups to reflect data availability for a particular amount of time from each country/territory: 0 years (unavailable), 1–5 years, 6–10 years, 11–15 years, 16–20 years, 21–25 years, or 26–30 years. In addition, we reported the version of ICD that each country/territory used to code deaths in each year. We limited data quality analysis to the years that countries/territories adopted the ICD-10 to code the deaths.
- *Coding quality*: Based on previous frameworks for classifying and coding injury morbidity and mortality [19, 20], we defined five types of problematic ICD-10 codes related to unintentional falls: (1) unspecified deaths (ICD-10 codes: R96, R98, R99), (2) injuries with undetermined intent (ICD-10 codes: Y30, Y31, Y34, Y87.2, Y89.9), (3) unspecified unintentional injuries (ICD-10 codes: X59), (4) unintentional falls with unspecified mechanism (ICD-10 codes: W19), and (5) unintentional falls with unknown occurrence place (the fourth digit code of W00-W19 is 9). The first three types of problematic codes have an impact on the estimate of overall unintentional falls mortality, while the latter two types of problematic codes affect the estimates of subgroup unintentional fall mortality by mechanism and by occurrence place. The proportion of five types of problematic codes was calculated as follows:

$$Proportion\ one=\frac{Number\ of\ unspecified\ deaths}{Number\ of\ total\ deaths}\times 100\%$$

$$Proportion\ two=\frac{Number\ of\ injury\ deaths\ with\ undetermined\ intent}{Number\ of\ injury\ deaths}\times 100\%$$

$$Proportion\ three=\frac{Number\ of\ unspecified\ unintentional\ injury\ deaths}{Number\ of\ unintentional\ injury\ deaths}\times 100\%$$

$$Proportion\ four=\frac{Number\ of\ unspecified\ unintentional\ fall\ deaths}{Number\ of\ unintentional\ fall\ deaths}\times 100\%$$

$$Proportion\ five=\frac{Number\ of\ unintentioanl\ fall\ deaths\ with\ unknown\ occurrence\ place}{Number\ of\ unintentional\ fall\ deaths}\times 100\%$$

### Models correcting problematic codes

The proportionate method was used to correct (redistribute) problematic codes to cause-specific codes in a specific year for each country/territory. The proportionate method assumes that deaths with problematic codes follow the same cause distribution as deaths with specific codes, and thus could be redistributed to cause-specific deaths [21]. Because only the first three types of problematic codes affect the overall unintentional fall mortality, we applied the proportionate method to redistribute the first three types of problematic codes (unspecified deaths, injury deaths with undetermined intent, and unspecified unintentional injury deaths). When the proportion of deaths with problematic codes was less than 30% in a specific year, we used the proportions of cause-specific deaths in the same year to redistribute deaths with problematic codes. When the proportion of deaths with problematic codes fell between 30 and 49% in a specific year, we used the proportions of cause-specific deaths for 3 years (the study year, the year before the study year, and the year after the study year) to redistribute deaths with problematic codes. When the proportion of deaths with problematic codes ranged between 50 and 69% in a specific year, we used the proportions of cause-specific deaths of 5 years (the study year, the 2 years before the study year, and the 2 years after the study year) to redistribute deaths with problematic codes. When the proportion of deaths with problematic codes in a specific year was equal to or greater than 70%, we used the proportions of cause-specific deaths of 7 years (the study year, the 3 years before the study year, and the 3 years after the study year) to redistribute deaths with problematic codes.

### Statistical analysis

Stacked bar charts and geographic maps were used to present the availability of unintentional fall mortality from 1990 and 2019 in the WHO Mortality Database. The proportions of the five types of deaths with problematic ICD-10 codes were calculated for each year and for each country/territory. To demonstrate the coding quality of mortality data related to unintentional falls across countries/territories, we graphed the distribution of each of the five proportions of deaths with problematic codes by economic level of country/territory. According to the World Bank Analytical Classifications 2019 [22], we classified countries/territories into four categories: low-income countries/territories (LICTs), lower middle-income countries/territories (LMICTs), upper middle-income countries/territories (UMICTs), and higher-income countries/territories (HICTs). Kruskal-Wallis rank sum test was used to examine differences in coding quality of unintentional fall mortality across the four income groups (Additional file 1**: Appendix 1**). In addition, we plotted trends in coding quality of unintentional fall mortality for each country/territory during the study period (Additional file 1**: Appendix 2**).

To assess the impact of problematic codes on injury statistics, we compared age-adjusted unintentional fall mortality among adults aged 65 years and older before and after correcting (redistributing) deaths with problematic codes. The direct method and the new WHO World Standard Population [23] were used to calculate age-adjusted mortality rates. The ratio of corrected/uncorrected mortality rate, computed based on the first three types of problematic codes (unspecified deaths, injury deaths with undetermined intent, and unspecified unintentional injury), was used to quantify the impact of deaths with problematic codes on each country’s mortality estimates.

In addition, we employed two strategies to assess the impact of coding quality on mortality changes over time. First, we selected the 2 years (2005 and 2015) when most countries/territories had mortality data to compare mortality changes between 2005 and 2015 before and after correcting deaths with problematic codes. Second, for countries/territories with 5 or more years of mortality data, we fitted linear regression (with year as the independent variable and age-adjusted mortality as the dependent variable) and compared the regression coefficient before and after correcting deaths with problematic codes for each country/territory (Additional file 1**: Appendix 3**).

“*p* ≤ 0.05” was considered statistically significant for two-sided statistical tests. SAS version 9.4, R version 4.0.4, and Excel spreadsheet 2016 were used for data analysis. The research protocol was approved by the Medical Ethics Committee of Central South University on 25 January 2021 (No. XYGW-2021-06). We complied with the Guidelines for Accurate and Transparent Health Estimates Reporting (GATHER) recommendations [24]. All analyses, interpretations and conclusions are attributed to the authors, not to WHO, which is responsible only for providing the raw data.

## Results

### Data availability

**Fig. 1:**
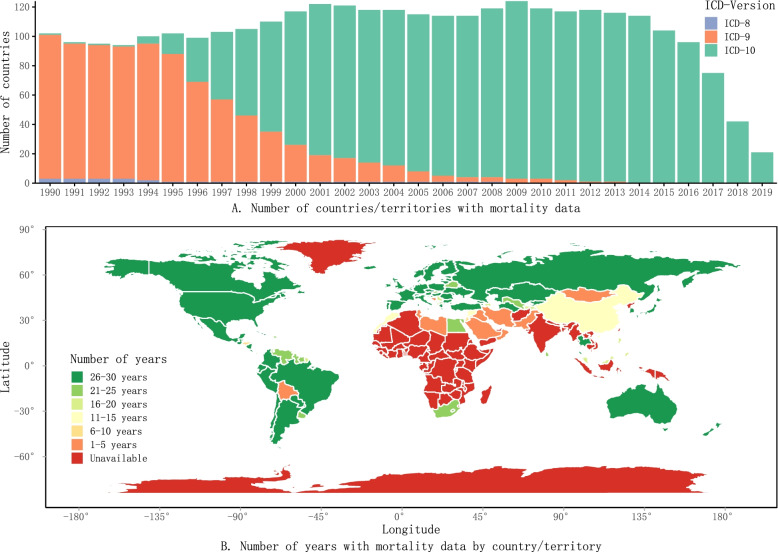
**Availability of mortality data in the World Health Organization Mortality Database, 1990–2019** ICD: International Classification of Disease. The map was created using the R package “maps”, which was imported from the public domain Natural Earth project

Figure 1 shows the availability of unintentional fall mortality for older adults in the WHO Mortality Database from 1990 to 2019. Of the 194 WHO member states, 124 countries/territories had at least 1 year of death data in the WHO Mortality Database. During the study period, the number of countries/territories with mortality data was highest in 2009 (124) and lowest in 2019 (21); most countries/territories with death data in the WHO Mortality Database adopted the ICD-9 from 1990 to 1995; but during and after the mid-1990s, the number of countries/territories adopting ICD-10 increased quickly (Fig. 1**A**). Of the countries/territories reporting data to the WHO, those from the Americas, western and northern Europe, Russia, Australia, and New Zealand reported data for the greatest number of years; countries/territories from Africa, Southern Asia and Oceania tended to report for fewer or no years (Fig. 1**B**).

### Coding quality

Among the 102 countries/territories that reported death data to the WHO and used the 4-digit ICD-10 codes (104 or 10 M lists) to classify the deaths, one was a low-income country/territory (LICT), 13 were lower middle-income countries/territories (LMICTs), 31 were upper middle-income countries/territories (UMICTs), and 57 were high-income countries/territories (HICTs).

**Fig. 2:**
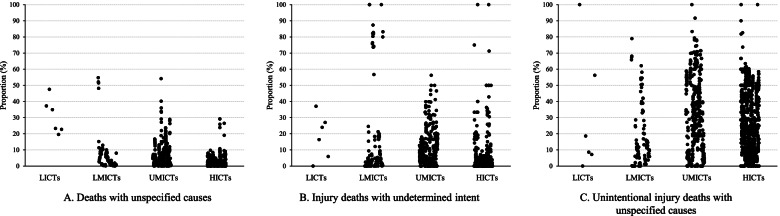
**Coding quality related to unintentional fall mortality among older adults in 102 countries/territories, 1990–2019** Notes: LICTs: low-income countries/territories, LMICTs: lower middle-income countries/territories, UMICTs: upper middle-income countries/territories, HICTs: high-income countries/territories. For deaths with unspecified causes, there were 6 years for LICTs, 121 years for LMICTs, 454 years for UMICTs, and 970 years for HICTs; for injury deaths with undetermined intent, there were 6 years for LICTs, 119 years for LMICTs, 454 years for UMICTs, and 955 years for HICTs; for unintentional injury deaths with unspecified causes, there were 6 years for LICTs, 116 years for LMICTs, 454 years for UMICTs, and 950 years for HICTs

Figure 2A shows that the proportion of deaths with unspecified causes varied significantly across the four types of countries/territories (*p* < 0.01). LICTs had the highest proportion (median: 29.12%, *P*_25_-*P*_75_: 22.88–36.68%) and HICTs the lowest (median: 0.68%, *P*_25_-*P*_75_: 0.20–1.94%). Note that each point in the figure represents data from a single year and the six points for LICTs were all from the same country/territory.

The proportions of injury deaths with undetermined intent and unspecified unintentional injury deaths were also generally lower in countries/territories with high income than in those with low income (Fig 2B and C). Notably, four particular years in high-income countries/territories had extremely high proportions of injury deaths with undetermined intent: 1998 for Saint Kitts and Nevis (100%), 2002 for Cayman Islands (100%), 2005 for Antigua and Barbuda (75%), and 2009 for Saudi Arabia (71%) (Fig. 2B). Similarly, for six particular years, individual high-income countries/territories reported a proportion of unspecified unintentional injury higher than 70% (Fig. 2C). Detailed reporting quality for the three measures for each country/territory and each year are available in the supplementary information (Additional file 1**: Appendix 2**).

**Fig. 3:**
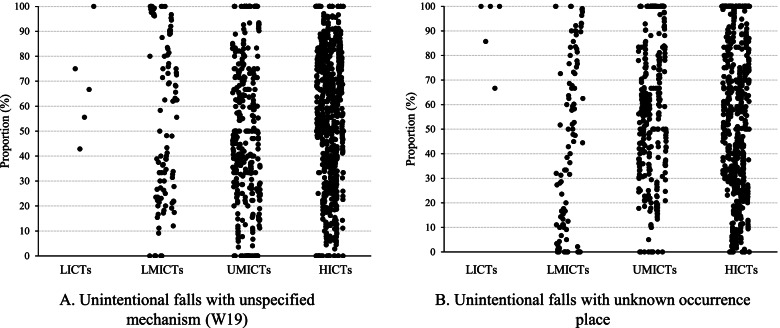
**Coding quality of unintentional fall mortality among older adults in 102 countries/territories, 1990–2019** Notes: LICTs: low-income countries/territories, LMICTs: lower middle-income countries/territories, UMICTs: upper middle-income countries/territories, HICTs: high-income countries/territories. For unintentional fall deaths with unspecified mechanisms, there were 5 years for LICTs, 114 years for LMICTs, 414 years for UMICTs, and 875 years for HICTs; for unintentional fall deaths with undetermined occurrence place, there were 5 years for LICTs, 114 years for LMICTs, 411 years for UMICTs, and 841 years for HICTs

Within the deaths that were coded as unintentional falls, the specificity involving the mechanism of falls and the occurrence place were highly similar and consistently poor **(**Fig. 3A and B). There were 80, 53, 51, and 63% of study years having a proportion of unintentional falls with unspecified mechanism (W19) over 50% in LICTs, LMICTs, UMICTs, and HICTs, respectively (Fig. 3A). The corresponding proportions for unintentional fall deaths with unknown occurrence place were 100, 42, 71, and 62% for the four types of countries/territories (Fig. 3B).

### Impact of coding quality

Of 94 countries/territories having both available death data and population data, we calculated the ratio of age-adjusted unintentional fall mortality for adults aged 65 years and older before and after correcting three types of problematic codes (unspecified deaths, injury deaths with undetermined intent, and unspecified unintentional injury deaths), and divided the countries/territories into six categories based on the value of the mortality rate ratio between corrected and uncorrected age-adjusted unintentional fall mortality (note: the UN WPP2019 does not offer population estimates for 6 countries/territories). Table 1 illustrates largely inconsistent impacts of coding quality on elderly unintentional fall mortality across the 94 countries/territories. Six countries/territories (Belarus, New Zealand, Singapore, Cuba, Hungary, and Mongolia) had the best unintentional fall mortality data, with the maximum ratio of corrected/uncorrected age-adjusted unintentional fall mortality) ranging from 1.00–1.10. Remarkably, 59 countries/territories had poor data, with a ratio greater than 50%.

**Table 1:** Ratio of unintentional fall mortality before and after correcting data coding in 94 countries/territories, 1990–2019

Level of quality	Country/territory (number of years, median, minimum-maximum)
Highest	**6 countries/territories:** Belarus (1, 1.00, 1.00–1.00), New Zealand (17, 1.02, 1.01–1.03), Singapore (7, 1.02, 1.01–1.03), Cuba (17, 1.03, 1.02–1.04), Hungary (24, 1.03, 1.00–1.06), Mongolia (1, 1.03, 1.03–1.03)
Higher	**10 countries/territories:** Malta (23, 1.00, 1.00–1.15), Switzerland (23, 1.07, 1.05–1.16), Turkey (9, 1.07, 1.02–1.17), Estonia (8, 1.08, 1.05–1.12), Romania (20, 1.08, 1.04–1.13), Lithuania (20, 1.09, 1.04–1.18), Japan (23, 1.10, 1.07–1.14), Sri Lanka (8, 1.10, 1.05–1.13), Bulgaria (6, 1.12, 1.04–1.17), Poland (20, 1.16, 1.10–1.19)
High	**7 countries/territories:** Kuwait (23, 1.03, 1.00–1.26), El Salvador (19, 1.11, 1.03–1.26), United States of America (19, 1.14, 1.11–1.25), Kyrgyzstan (17, 1.16, 1.05–1.23), Latvia (11, 1.17, 1.11–1.22), Germany (21, 1.20, 1.15–1.23), Greece (4, 1.27, 1.19–1.29)
Low	**8 countries/territories:** Belize (20, 1.01, 1.00–1.40^a^), Croatia (23, 1.08, 1.04–1.34), Armenia (4, 1.22, 1.09–1.34), Republic of Moldova (12, 1.25, 1.17–1.33), Ireland (9, 1.27, 1.16–1.36), Slovakia (4, 1.29, 1.01–1.35), Spain (19, 1.30, 1.25–1.40^a^), Serbia (1, 1.34, 1.34–1.34)
Lower	**4 countries/territories:** China, Hong Kong SAR (17, 1.24, 1.11–1.48), Philippines (10, 1.28, 1.05–1.41), Honduras (6, 1.30, 1.26–1.49), Brunei Darussalam (4, 1.34, 1.18–1.44)
Lowest	**59 countries/territories:** Grenada (18, 1.00, 1.00–1.88), Virgin Islands (USA) (17, 1.00, 1.00–1.52), Saint Vincent and Grenadines (18, 1.02, 1.00–1.66), Barbados (14, 1.04, 1.01–2.87), Saint Lucia (19, 1.14, 1.00–2.40), Iceland (16, 1.15, 1.00–1.98), Panama (20, 1.16, 1.05–1.59), Czech Republic (26, 1.18, 1.06–2.61), Colombia (21, 1.22, 1.14–1.58), Puerto Rico (19, 1.22, 1.06–1.50), Maldives (10, 1.25, 1.04–1.94), Aruba (18, 1.26, 1.04–1.83), Canada (18, 1.26, 1.16–1.62), Chile (22, 1.26, 1.11–2.44), Australia (20, 1.28, 1.24–2.00), Suriname (20, 1.28, 1.02–2.00), Venezuela (19, 1.33, 1.27–1.55), Mauritius (15, 1.34, 1.06–2.26), Brazil (23, 1.35, 1.22–1.80), Norway (21, 1.35, 1.08–2.24), Netherlands (23, 1.38, 1.13–2.26), Republic of Korea (23, 1.39, 1.07–1.70), Bahamas (16, 1.41, 1.07–2.56), Belgium (17, 1.42, 1.27–1.52), Luxembourg (20, 1.47, 1.27–1.93), Denmark (25, 1.50, 1.04–2.63), Antigua and Barbuda (18, 1.54, 1.02–2.00), Portugal (14, 1.60, 1.24–1.78), United Kingdom (16, 1.60, 1.30–1.71), Austria (18, 1.66, 1.08–1.91), Ecuador (21, 1.66, 1.48–1.93), Nicaragua (22, 1.69, 1.25–5.02), Fiji (11, 1.70, 1.00–2.70), France (17, 1.70, 1.64–1.81), Jordan (6, 1.76, 1.25–2.17), Thailand (14, 1.76, 1.52–2.89), Sweden (22, 1.78, 1.66–1.98), Mexico (20, 1.81, 1.66–1.95), Trinidad and Tobago (14, 1.81, 1.33–2.07), Guyana (14, 1.82, 1.47–3.44), Georgia (15, 1.96, 1.35–6.20), Haiti (6, 1.97, 1.74–3.59), Peru (19, 1.99, 1.35–3.45), Dominican Republic (18, 2.05, 1.68–2.57), Italy (15, 2.22, 1.98–2.50), Paraguay (22, 2.22, 1.73–3.15), Uruguay (20, 2.30, 1.76–2.86), Cyprus (15, 2.33, 1.67–3.09), Israel (21, 2.41, 1.83–2.75), Oman (4, 2.70, 1.84–4.02), Argentina (22, 2.74, 1.84–3.12), Costa Rica (22, 2.91, 2.59–4.26), Lebanon (3, 3.25, 2.50–3.59), Guatemala (13, 3.39, 2.39–5.87), Saudi Arabia (1, 4.28, 4.28–4.28), Jamaica (13, 4.29, 2.23–10.00), Morocco (15, 4.38, 1.79–9.86), Bolivia (4, 6.93, 6.58–7.27), Tunisia (2, 7.30, 6.35–8.24)

Note: Countries were divided into six categories according to the maximum ratio of corrected/uncorrected unintentional fall mortality for adults aged 65 years and older: ① highest, 1.00–1.09; ② higher, 1.10–1.19; ③ high, 1.20–1.29; ④ low, 1.30–1.39; ⑤ lower, 1.40–1.49; ⑥ lowest, ≥1.50

^a^The maximum value was less than 1.40 but was rounded to 1.40 when two decimal places were kept

**Fig. 4:**
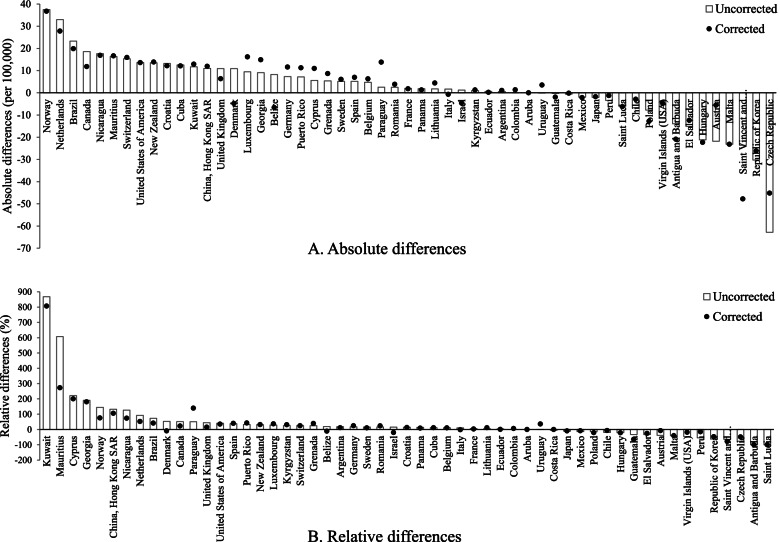
**Absolute and relative differences in age-adjusted unintentional fall mortality rates among older adults between 2005 and 2015 in 55 countries and territories before and after correcting data coding** Notes: Uncorrected: Mortality rates that were not corrected to adjust for the impact of unspecified deaths, injury deaths with undetermined intent, and unspecified unintentional injury deaths. Corrected: Mortality rates after correction for the impact of unspecified deaths, injury deaths with undetermined intent, and unspecified unintentional injury deaths

When comparing changes in elderly age-adjusted unintentional fall mortality between 2005 and 2015 for the 55 countries/territories with mortality data, either the absolute rate difference or the relative rate changes, or both, changed somewhat over time in most countries/territories (Fig. 4A and B). Strikingly, increases became decreases in 4 countries (Belize, Denmark, Israel, and Italy) and a reduction in Uruguay changed to be an increase after correcting the problematic codes. In addition, among countries/territories whose mortality changes remained in the same direction (increase or decrease) between 2005 and 2015, the largest alterations in relative mortality changes occurred in several countries, including Mauritius (from 607 to 273%) and Paraguay (from 51 to 139%).

Of the 82 countries/territories with mortality data for 5 or more years, 29 of the 48 countries/territories with positive regression coefficients were statistically significant (*p* < 0.05) before correction. 17 of the 34 countries/territories with negative regression coefficients were statistically significant (*p* < 0.05) before correction (Table 2).

**Table 2:** Regression coefficients of year on age-adjusted mortality rate in 82 countries/territories when uncorrected and corrected

Country/territory	*b* (*S*_*b*_)	*b’*(*S*_*b′*_)	Country/territory	*b*(*S*_*b*_)	*b’*(*S*_*b′*_)
Netherlands	2.92 (0.20)^*^	2.22 (0.23)^*^	Colombia	0.10 (0.06)	0.10 (0.06)
Australia	2.11 (0.08)^*^	2.04 (0.08)^*^	Portugal	0.10 (0.41)	0.52 (0.51)
Nicaragua	1.94 (0.24)^*^	2.23 (0.31)^*^	Barbados	0.09 (0.18)	0.47 (0.28)
Canada	1.91 (0.18)^*^	1.33 (0.13)^*^	Morocco	0.09 (0.03)^*^	0.10 (0.07)
Georgia	1.78 (0.44)^*^	2.93 (0.66)^*^	Honduras	0.03 (0.25)	0.16 (0.28)
Croatia	1.50 (0.37)^*^	1.30 (0.29)^*^	Israel	0.02 (0.04)	−0.32 (0.11)^*^
United States of America	1.45 (0.04)^*^	1.42 (0.04)^*^	Dominican Republic	0.01 (0.04)	−0.03 (0.08)
Grenada	1.39 (0.64)^*^	2.22 (1.05)	Costa Rica	0.00 (0.06)	0.22 (0.16)
Chile	1.02 (0.33)^*^	1.20 (0.31)^*^	Peru	−0.01 (0.05)	0.17 (0.07)^*^
New Zealand	1.01 (0.20)^*^	1.03 (0.20)^*^	Guatemala	−0.05 (0.01)^*^	−0.29 (0.07)^*^
Germany	0.93 (0.12)^*^	1.22 (0.15)^*^	Republic of Moldova	−0.08 (0.19)	−0.21 (0.22)
Cyprus	0.89 (0.21)^*^	1.05 (0.51)	Turkey	−0.09 (0.91)	− 0.48 (0.81)
Switzerland	0.87 (0.09)^*^	0.89 (0.10)^*^	Estonia	−0.13 (0.36)	− 0.13 (0.36)
United Kingdom	0.87 (0.10)^*^	0.60 (0.12)^*^	France	−0.13 (0.10)	− 0.44 (0.19)^*^
Mauritius	0.83 (0.26)^*^	0.63 (0.32)	Belize	−0.15 (0.75)	− 0.16 (0.82)
Sweden	0.82 (0.08)^*^	1.10 (0.13)^*^	Kyrgyzstan	−0.15 (0.07)^*^	− 0.18 (0.08)^*^
Brazil	0.78 (0.23)^*^	−0.29 (0.37)	Japan	−0.18 (0.02)^*^	−0.16 (0.02)^*^
Fiji	0.73 (0.51)	1.33 (1.14)	Singapore	−0.20 (0.25)	−0.18 (0.26)
Saint Vincent and Grenadines	0.65 (0.51)	0.56 (0.72)	Aruba	−0.23 (0.49)	−0.29 (0.59)
Puerto Rico	0.64 (0.21)^*^	0.92 (0.23)^*^	Panama	−0.23 (0.19)	−0.64 (0.22)^*^
Philippines	0.58 (0.14)^*^	0.78 (0.27)^*^	Virgin Islands (USA)	−0.24 (0.45)	− 0.13 (0.46)
Cuba	0.52 (0.28)	0.59 (0.29)	Argentina	−0.25 (0.07)^*^	−0.32 (0.12)^*^
Bulgaria	0.48 (0.43)	0.31 (0.44)	Trinidad and Tobago	−0.27 (0.24)	−0.46 (0.39)
China, Hong Kong SAR	0.44 (0.10)^*^	0.39 (0.12)^*^	Antigua and Barbuda	−0.29 (0.29)	−0.33 (0.46)
Thailand	0.44 (0.02)^*^	0.72 (0.05)^*^	Mexico	−0.34 (0.04)^*^	−0.44 (0.07)^*^
Spain	0.42 (0.05)^*^	0.54 (0.07)^*^	Venezuela	−0.34 (0.16)^*^	−0.66 (0.17)^*^
Saint Lucia	0.38 (0.33)	0.55 (0.37)	Ireland	−0.35 (0.47)	−0.02 (0.45)
Paraguay	0.36 (0.05)^*^	1.20 (0.13)^*^	Suriname	−0.54 (0.58)	−0.91 (0.69)
Belgium	0.35 (0.13)^*^	0.34 (0.16)	Jordan	−0.61 (0.32)	−0.62 (0.69)
Luxembourg	0.35 (0.23)	−0.46 (0.39)	El Salvador	−0.85 (0.32)^*^	−0.88 (0.41)^*^
Maldives	0.34 (1.07)	1.07 (1.77)	Republic of Korea	−1.16 (0.33)^*^	−0.81 (0.35)^*^
Jamaica	0.33 (0.07)^*^	0.80 (0.19)^*^	Bahamas	−1.20 (0.69)	−0.96 (0.87)
Lithuania	0.32 (0.18)	0.49 (0.17)^*^	Austria	−1.22 (0.20)^*^	0.23 (0.17)
Latvia	0.25 (0.32)	0.32 (0.38)	Guyana	−1.25 (0.30)^*^	−2.02 (0.72)^*^
Kuwait	0.20 (0.19)	0.24 (0.22)	Malta	−1.33 (0.50)^*^	−1.37 (0.50)^*^
Haiti	0.19 (0.23)	0.18 (0.50)	Poland	−1.44 (0.20)^*^	−1.86 (0.22)^*^
Italy	0.19 (0.07)^*^	−0.08 (0.09)	Sri Lanka	−1.88 (0.24)^*^	−1.78 (0.23)^*^
Romania	0.17 (0.05)^*^	0.26 (0.06)^*^	Norway	−2.59 (0.69)^*^	−0.94 (0.46)
Ecuador	0.16 (0.08)	0.13 (0.12)	Denmark	−3.66 (0.82)^*^	−3.63 (0.57)^*^
Iceland	0.13 (0.48)	1.32 (0.52)^*^	Hungary	−5.50 (0.49)^*^	−5.46 (0.48)^*^
Uruguay	0.11 (0.04)^*^	0.34 (0.08)^*^	Czech Republic	−6.37 (0.40)^*^	−5.57 (0.43)^*^

Notes: b, regression coefficients when uncorrected; b’, regression coefficients when corrected;

^*^ *p*-value ≤0.05

Of the 48 countries/territories with positive regression coefficients, after correcting the problematic codes, the direction reversed in 5 countries/territories (Brazil, Dominican Republic, Israel, Italy, Luxembourg), the test results reversed to insignificant in 7 countries (Belgium, Brazil, Cyprus, Grenada, Italy, Mauritius, Morocco) and the test results reversed to significant in 3 countries (Iceland, Israel, Lithuania).

Of the 34 countries/territories with negative regression coefficients, after correcting the problematic codes, the direction reversed in 3 countries (Austria, Costa Rica, Peru), the test results reversed to insignificant in 2 countries (Austria, Norway) and the test results reversed to significant in 3 countries (France, Panama, Peru).

## Discussion

### Summary of main findings

This study assessed reporting quality for elderly unintentional fall mortality data over the past three decades from the WHO Mortality Database and quantified the impact of reporting quality on estimated mortality data. The study generated three primary findings: (a) only 64% (124/194) of WHO member countries reported mortality data to the WHO for at least 1 year during 1990–2019, and lack of reporting was more common for underdeveloped countries/territories in the WHO Mortality Database; (b) problematic codes related to elderly unintentional fall deaths were common for many countries/territories with available data in the WHO Mortality Database, including frequent reporting of deaths coded as falls with unspecified mechanism (W19) and with unknown occurrence place; and (c) problematic codes largely or moderately distorted elderly unintentional fall mortality rates in certain years for some countries/territories, and therefore created potentially-misleading data to examine trends over time or compare elderly fall rates across countries/territories.

### Interpretation of findings

#### Data unavailability

Open data are critical to guide global, regional, and national public health actions like the post-2015 UN SDGs [25, 26]. Unfortunately, mortality data from 1990 to 2019 are unavailable in the WHO Mortality Database for 70 WHO member states. This finding replicates the result from two earlier studies that indicated that the WHO Mortality Database did not have data from 75 countries in 1990–2003 [8] and that only 83 countries provided death registration data from 2000 to 2009 [27]. The results suggest minimal progress in data-sharing over time.

The lack of data in the WHO Mortality Database is generally ascribed to at least four causes. First, some countries/territories do not have a vital registration system to gather basic health indicators. For example, The Indian national vital registration system is still under development [28]. Second, the vital registration system in some countries was destroyed by political turmoil and war (e.g., Iraq, Syria, Libya) [29, 30]. Third, some countries/territories have not adopted the ICD codes yet, or still use non-standard ICD codes to gather death data [31] for their vital registration systems. Fourth, some countries/territories decline to submit their data to the WHO Mortality Database despite the fact that they possess the data. For example, Vietnam does not report data to the WHO Mortality Database regularly although it has had an established death registration system (“A6 death register”) for almost 30 years [32]. These governments may worry about unwanted outcomes of submitting mortality data for public distribution, such as data misinterpretation or misuse, disruption of domestic legislation, and/or loss of data ownership [33].

#### Coding quality

We considered two types of evidence about coding quality. First, we considered problematic ICD codes for deaths in general by examining the rates of deaths with unspecified causes, injury deaths with undetermined intent, and unintentional injury deaths with unspecified causes. Replicating previous results from Mathers et al. [8] and Bhalla et al. [27], we found high proportions of such problematic codes across time and across countries/territories.

Second, we considered two problematic codes within those deaths caused by unintentional falls among older adults: unintentional falls with unspecified mechanism and unintentional falls with unknown occurrence place. Our findings underscore the severity of two types of problematic codes. Problematic ICD codes were common for elderly unintentional fall mortality, with the proportions of unintentional falls with unspecified mechanism ranging from 50 to 66.67% and of unintentional falls with unknown occurrence place ranging from 33.33 to 100% (see Additional file 1: Appendix 1).

Problematic codes are attributed to a range of factors in previous research, including a) lack of necessary knowledge and skills in disease diagnosis and use of ICD codes by medical practitioners and coders [34–37]; b) heavy workload for medical practitioners and coders [38]; and c) lack of work enthusiasm for many coders [39, 40].

#### Influence of problematic codes

Three previous studies examined recent trends in elderly unintentional fall mortality in the United States and suggested that the recent mortality increases in elderly unintentional fall may be partially related to the improvement in coding practice rather than entirely to actual increases in the rates of the falls [15–17]. This finding raises concerns about global data.

By examining global rather than only United States data, our research considered additional relevant indicators to assess the quality related to elderly unintentional fall mortality. We found that the first three types of problematic codes had a varying influence on elderly unintentional fall mortality across countries/territories and over time for some countries/territories.

More broadly, we replicated previous reports from the United States [15–17] and found that poor-quality data may seriously or moderately bias estimates of unintentional fall mortality in many countries/territories. With imprecise coding, estimates may be significantly distorted and comparisons of mortality rates across countries/territories and over time appear even to be subverted in some cases. These findings emphasize the need to attend carefully to the reporting quality of crude data when using the WHO Mortality Database for injury prevention research and policy-making.

### Policy implications

Our findings raise several policy implications. First, there is urgent need to improve data availability of the WHO Mortality Database. For countries/territories without an established vital registration system, resources should be directed to develop a system that can collect high-quality data. WHO has developed programs, guidelines and tools, including a World Health Data Hub, to aid these countries/territories [41–44]. For countries that collect mortality data but do not report it to the WHO, efforts should be made to clarify the significance of data-sharing to support both global and national health decision-making and development of health policy.

Second, global and national efforts should be taken to improve the quality of elderly unintentional fall mortality data. This may require addressing the challenge of improving infrastructure capacities in many LMICTs, including recruiting and maintaining adequate and qualified medical certifiers to code data. Regular inspections of data should be conducted in a rigorous manner to detect and correct problematic disease diagnoses and ICD codes. Information technologies (e.g., automatic machine learning models based electronic health records) could be introduced to improve data accuracy [45, 46].

Last, researchers and policy-makers should be aware of the limitations of the crude data included in the WHO Mortality Database and consider how to clean and/or interpret the data given those limitations. The WHO might consider organizing a group of experts to assess the quality of mortality data in the WHO Mortality Database and standardize a correction approach to resolve data issues and release corrected data estimates. The WHO might also organize a team to conduct a systematic review of national policy documents for mortality data collection to help understand the reasons for poor data availability and low data quality and to identify corresponding solutions.

### Study limitations

This study was primarily limited by lack of external information that is needed to systematically assess data quality. Without such information, we relied on proxies like problematic data coding but were unable to fully assess completeness and misclassification of mortality data. In related areas like road traffic mortality data, under-reporting and or over-reporting are demonstrated by comparing health data with highway safety reports [47]. We also were unable to examine misclassification of data. Among old adults, a minor or moderate fall injury may lead to death after a lengthy interval [48]. In those cases, especially as the time interval increases, the underlying cause of death may be coded as a terminal illness rather due to an unintentional fall, and our data were unable to uncover such situations [49].

Further, because we were unable to gather policy documents concerning mortality data collection for each country/territory included in the WHO Mortality Database due to language barriers and/or unavailability of relevant documents, we cannot conduct a systematic analysis of policy documents to explore factors influencing data availability and reporting quality. Future research might address this question. Finally, the linear regression analysis that we conducted may not capture non-linear time trends in age-adjusted unintentional fall mortality.

## Conclusion

Despite global efforts across many years, the availability and quality of data related to elderly unintentional fall mortality in the WHO Mortality Database is poor. Data availability and quality were comparatively worse in LICTs in Africa, Southern Asia and Oceania. Varying data quality across countries/territories or over time have substantial impact on mortality estimates and comparisons of mortality across time and across countries.

We recommend continued global and national efforts to increase the availability and quality of unintentional fall mortality data in the WHO Mortality Database. While that happens, researchers and policy-makers should interpret and use results based on crude data with caution. In addition, we recommend the WHO lead an effort to assess the quality of available mortality data in the WHO Mortality Database, develop correction approaches as needed, and provide corrected data for public use.

## Supplementary Information

**Additional file 1: Appendix 1.** Coding quality by county/territory income. **Appendix 2.** Country-specific coding quality. **Appendix 3.** Crude and corrected age-adjusted unintentional fall mortality. **Appendix 4.** Flow chart of selection of eligible countries/territories. **Appendix 5.** GATHER checklist

## Data Availability

Datasets for the current study are available in the WHO Mortality Database, https://www.who.int/data/data-collection-tools/who-mortality-database, and UN World Population Prospects 2019, https://population.un.org/wpp/Download/Standard/Population/.

## Abbreviations

GATHER: Guidelines for Accurate and Transparent Health Estimates Reporting
GBD: Global Burden of Disease
HICTs: higher-income countries/territories (HICTs)
ICD: International Classification of Disease
LICTs: low-income countries/territories
LMICTs: lower middle-income countries/territories
SDGs: Sustainable Development Goals
UMICTs: upper middle-income countries/territories
UN: United Nations
WHO: World Health Organization
WPP: World Population Prospects
