# Supplementary material for: Coding quality of deaths and its impact on elderly unintentional fall mortality data from 1990 to 2019: a retrospective analysis of the WHO Mortality Database
Source: BMC Geriatr. 2022 Jan 24;22:72. doi: 10.1186/s12877-021-02744-3 (PMC8785483; doi:10.1186/s12877-021-02744-3)
Supplement: Supplementary file 1 — Additional file 1: Appendix 1. Coding quality by county/territory income. Appendix 2. Country-specific coding quality. Appendix 3. Crude and corrected age-adjusted unintentional fall mortality. Appendix 4. Flow chart of selection of eligible countries/territories. Appendix 5. GATHER checklist [file 12877_2021_2744_MOESM1_ESM.docx]

**Supplemental Information**

**Coding quality and it impact related to** **elderly unintentional fall mortality from 1990 to 2019: A retrospective analysis of the WHO Mortality Database**

Junjie Hua, Peishan Ning, Peixia Cheng, Zhenzhen Rao, Jieyi He, Wangxin Xiao, Li Li, Yanhong Fu, Ruotong Li, Jie Li, Wanhui Wang, David C. Schwebel, Guoqing Hu

**Table of contents**

**Introduction**…………………………………………………………………………………2

**Appendix 1.** Coding quality by county/territory income………………………………3

**Appendix 2.** Country-specific coding quality……………………………………………4

Proportion of deaths with unspecified causes……………………………………………4

Proportion of injury deaths with undetermined intent………………………………….55

Proportion of unspecified unintentional injury deaths………………………………...106

Proportion of unintentional falls with unspecified mechanism………………………...157

Proportion of unintentional falls with unknown occurrence place……………………...208

**Appendix 3.** Crude and corrected age-adjusted unintentional fall mortality ……………….259

**Appendix 4.** Flow chart of selection of eligible countries/territories………………………300

**Appendix 5.** GATHER checklist……………………………………………………301

**Introduction**

This appendix provides detailed results that are not presented in the main text of manuscript. Five types of problematic codes were assessed: unspecified deaths, injury deaths with undetermined intent, unspecified unintentional injury, unintentional falls with unspecified mechanism, and unintentional falls with unknown occurrence place.

Differences in proportion of problematic codes were examined across level of country/territory income using Kruskal-Wallis rank sum test (**Appendix 1**).

Scatter plots present the relationship between proportion of deaths with each of the five types of problematic codes separately for each country/territory (**Appendix 2**). A curve was fitted to demonstrate the trend of each quality indicator over time. The shaded area in each figure illustrates the 95% confidence interval of estimated proportion. Points outside of the shaded area represent outliers that fall out of the 95% confidence interval.

**Appendix 3** shows the relationship between age-adjusted unintentional fall mortality and years based on locally estimated scatterplot smoothing (LOESS).

**Appendix 1. Coding quality by county/territory income**

**Table 1. Distribution of coding quality indicators for each income group, 1990 to 2019**

| Quality indicator | Economic level^a^ | Median | *P*_25_ | *P*_75_ | Min | Max | *P*-value^b^ |
| --- | --- | --- | --- | --- | --- | --- | --- |
| Deaths with unspecified causes | LICTs | 29.12 | 22.88 | 36.68 | 19.54 | 47.53 | <0.01 |
|  | LMICTs | 1.63 | 0.56 | 5.63 | 0.00 | 54.72 |  |
|  | UMICTs | 1.48 | 0.58 | 5.41 | 0.00 | 54.16 |  |
|  | HICTs | 0.68 | 0.20 | 1.94 | 0.00 | 29.24 |  |
| Injury deaths with undetermined intent | LICTs | 20.20 | 8.51 | 26.24 | 0.00 | 37.04 | <0.01 |
|  | LMICTs | 2.85 | 0.30 | 13.74 | 0.00 | 100.00 |  |
|  | UMICTs | 4.05 | 0.39 | 12.41 | 0.00 | 56.25 |  |
|  | HICTs | 0.34 | 0.00 | 2.07 | 0.00 | 100.00 |  |
| Unintentional injury deaths with unspecified causes | LICTs | 13.59 | 7.50 | 46.84 | 0.00 | 100.00 | <0.01 |
|  | LMICTs | 9.87 | 1.27 | 20.13 | 0.00 | 78.97 |  |
|  | UMICTs | 24.18 | 4.06 | 46.68 | 0.00 | 100.00 |  |
|  | HICTs | 15.02 | 4.65 | 36.17 | 0.00 | 100.00 |  |
| Unintentional falls with unspecified mechanism | LICTs | 66.67 | 55.56 | 75.00 | 42.86 | 100.00 | <0.05 |
|  | LMICTs | 60.17 | 26.40 | 89.90 | 0.00 | 100.00 |  |
|  | UMICTs | 50.00 | 28.43 | 75.00 | 0.00 | 100.00 |  |
|  | HICTs | 60.85 | 37.13 | 79.82 | 0.00 | 100.00 |  |
| Unintentional falls with unknown occurrence place | LICTs | 100.00 | 85.71 | 100.00 | 66.67 | 100.00 | <0.01 |
|  | LMICTs | 33.33 | 2.90 | 73.03 | 0.00 | 100.00 |  |
|  | UMICTs | 66.67 | 44.75 | 100.00 | 0.00 | 100.00 |  |
|  | HICTs | 66.44 | 32.73 | 100.00 | 0.00 | 100.00 |  |

^a^ LICTs: low-income countries/territories, LMICTs: lower middle-income countries/territories, UMICTs: upper middle-income countries/territories, HICTs: high-income countries/territories.

^b^ The Kruskal-Wallis rank sum test examined differences in each of five types of quality indicators across county/territory economic level.

**Appendix 2. Country-specific coding quality**

**
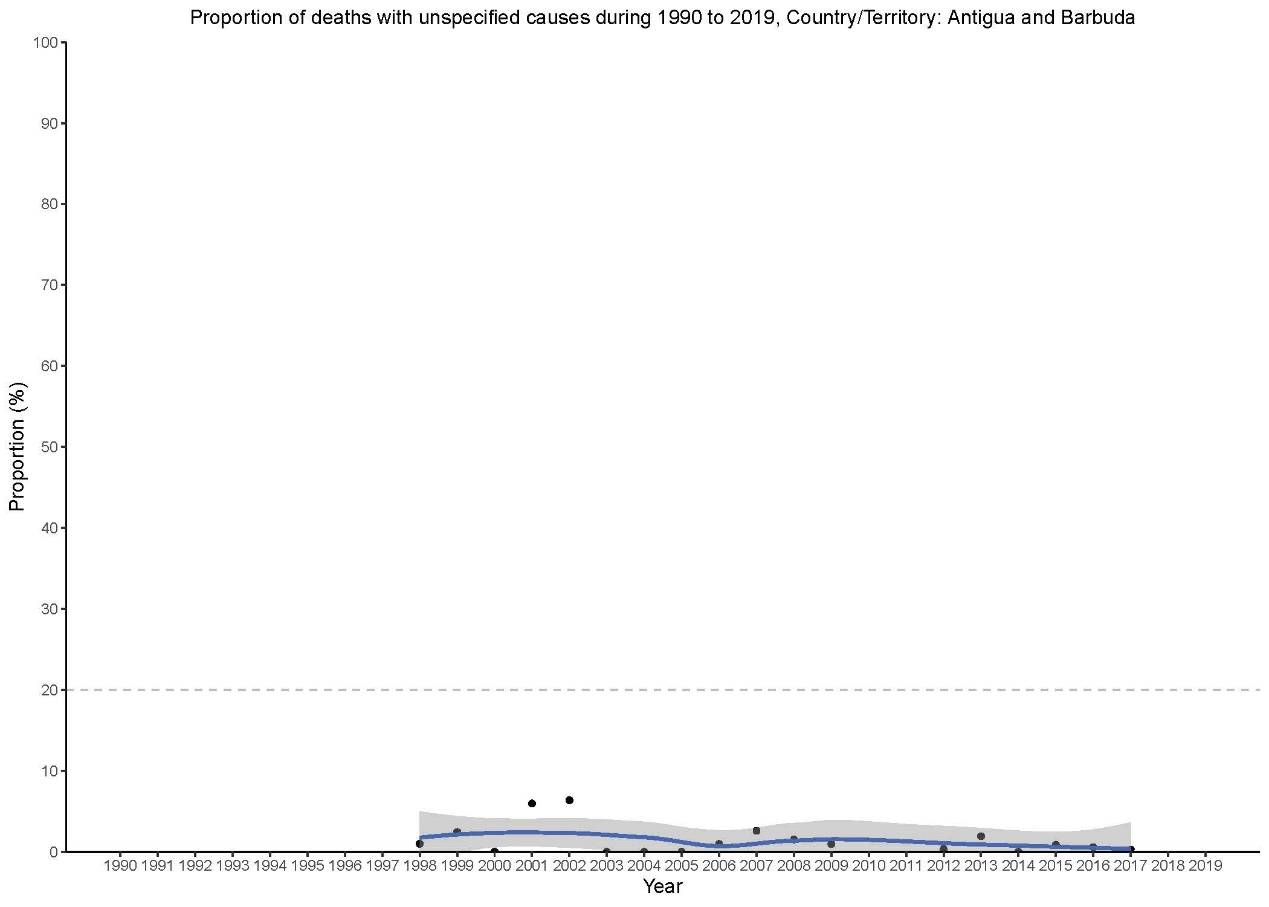

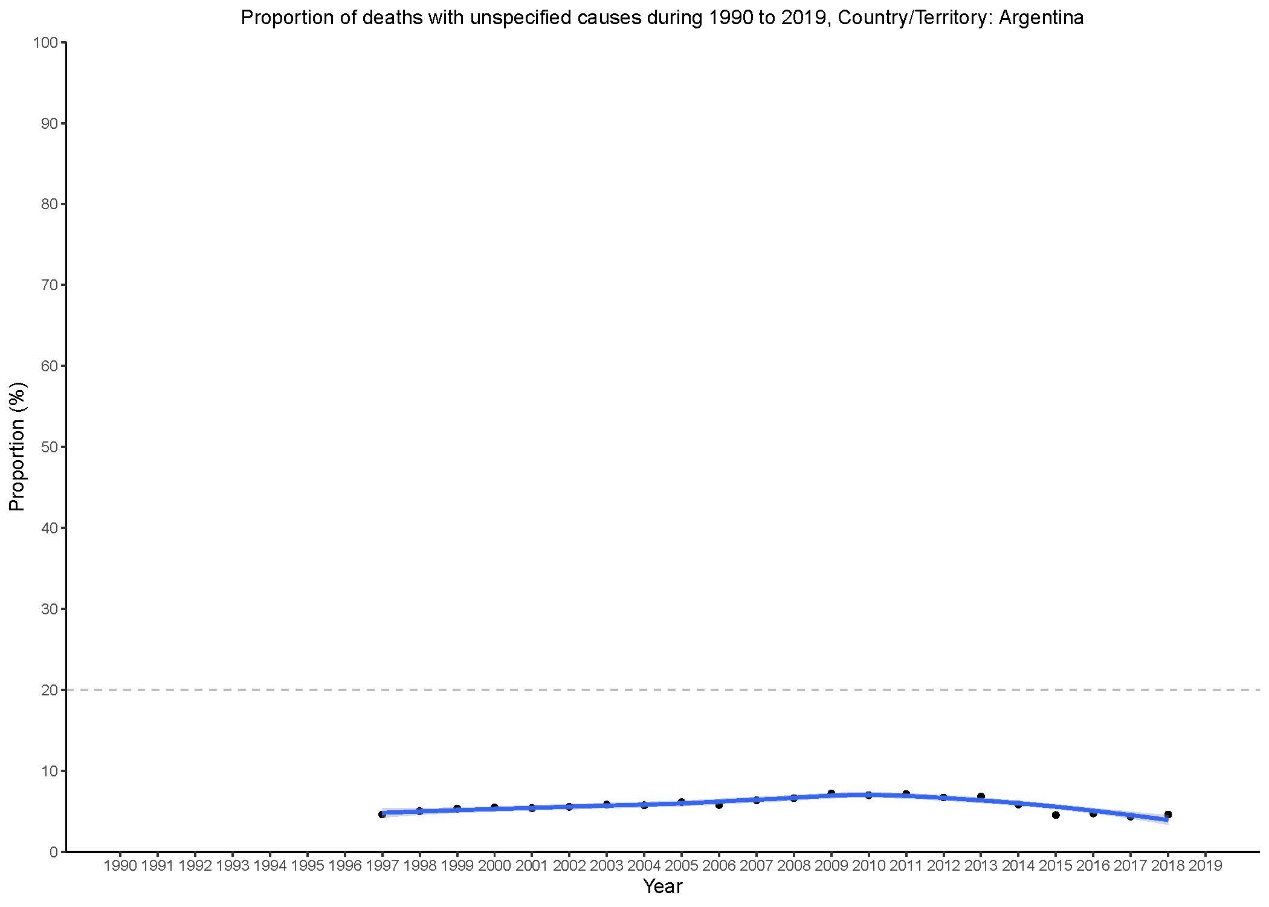

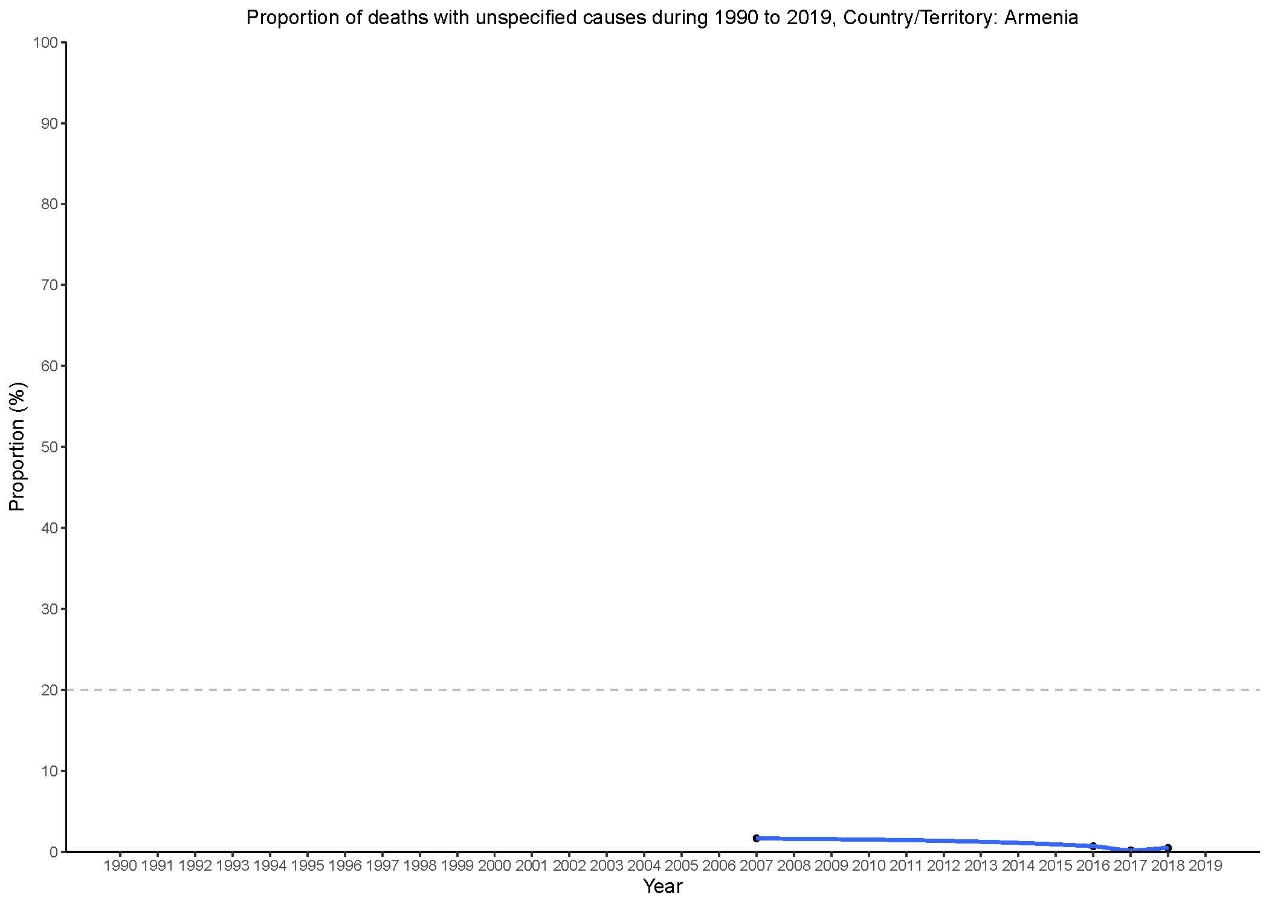

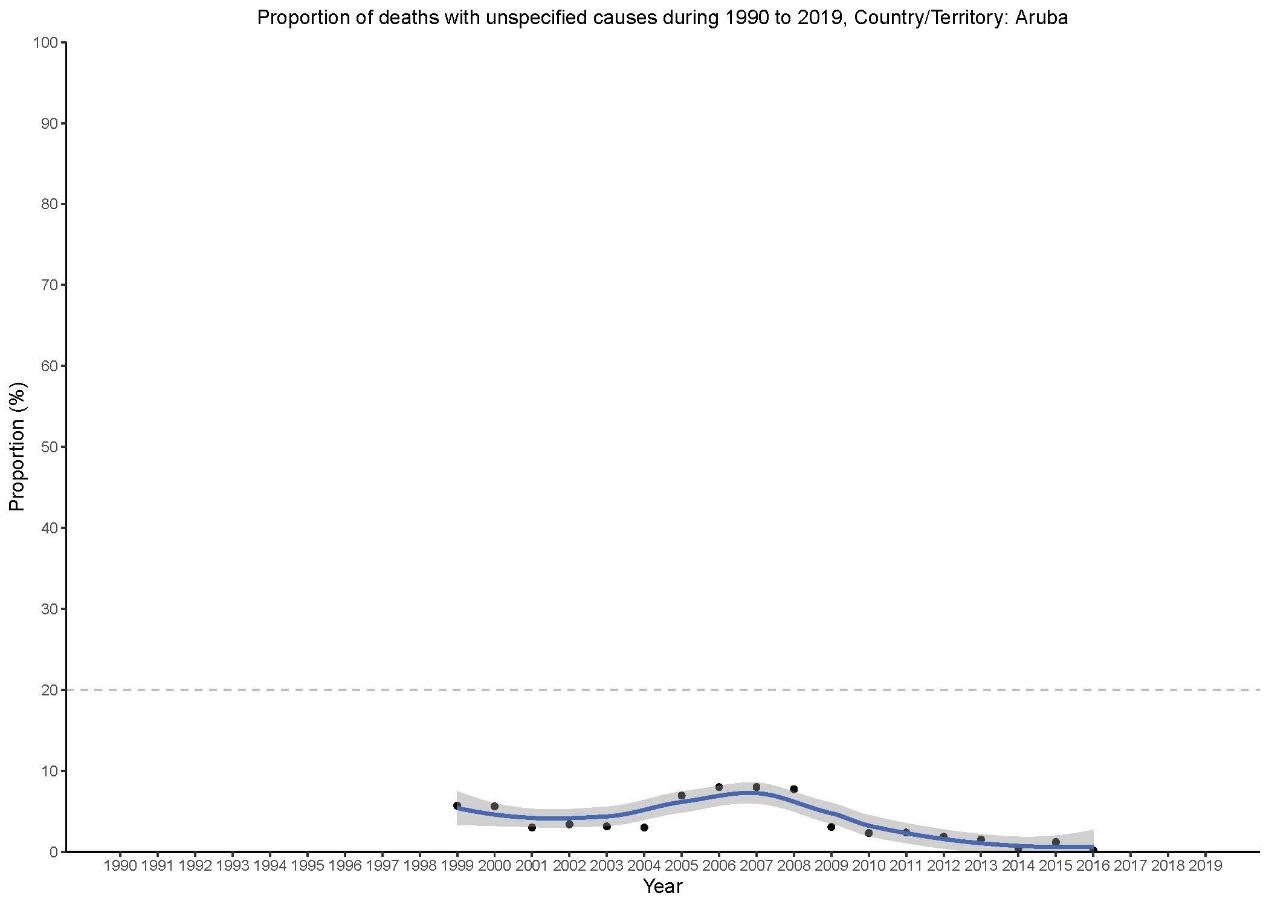

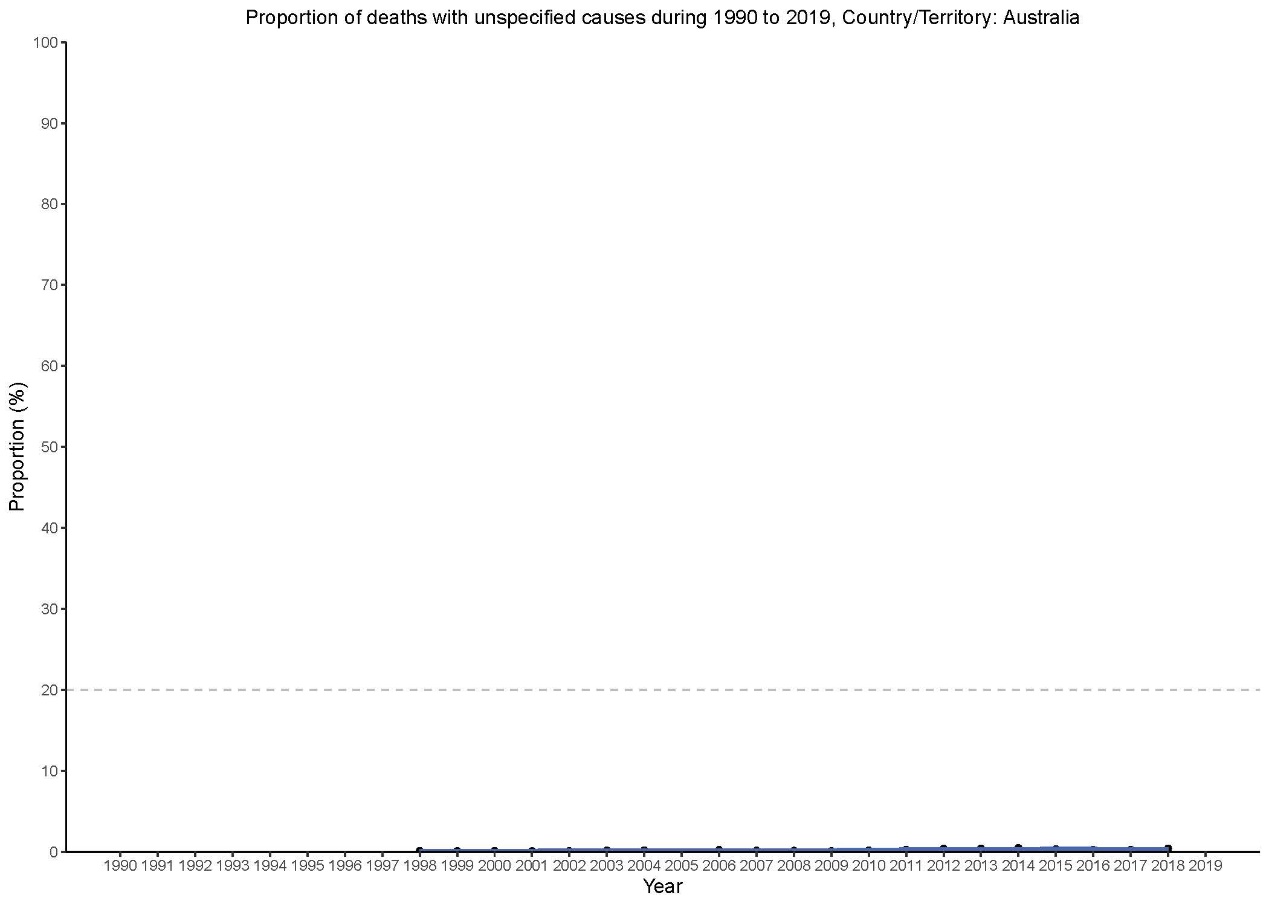

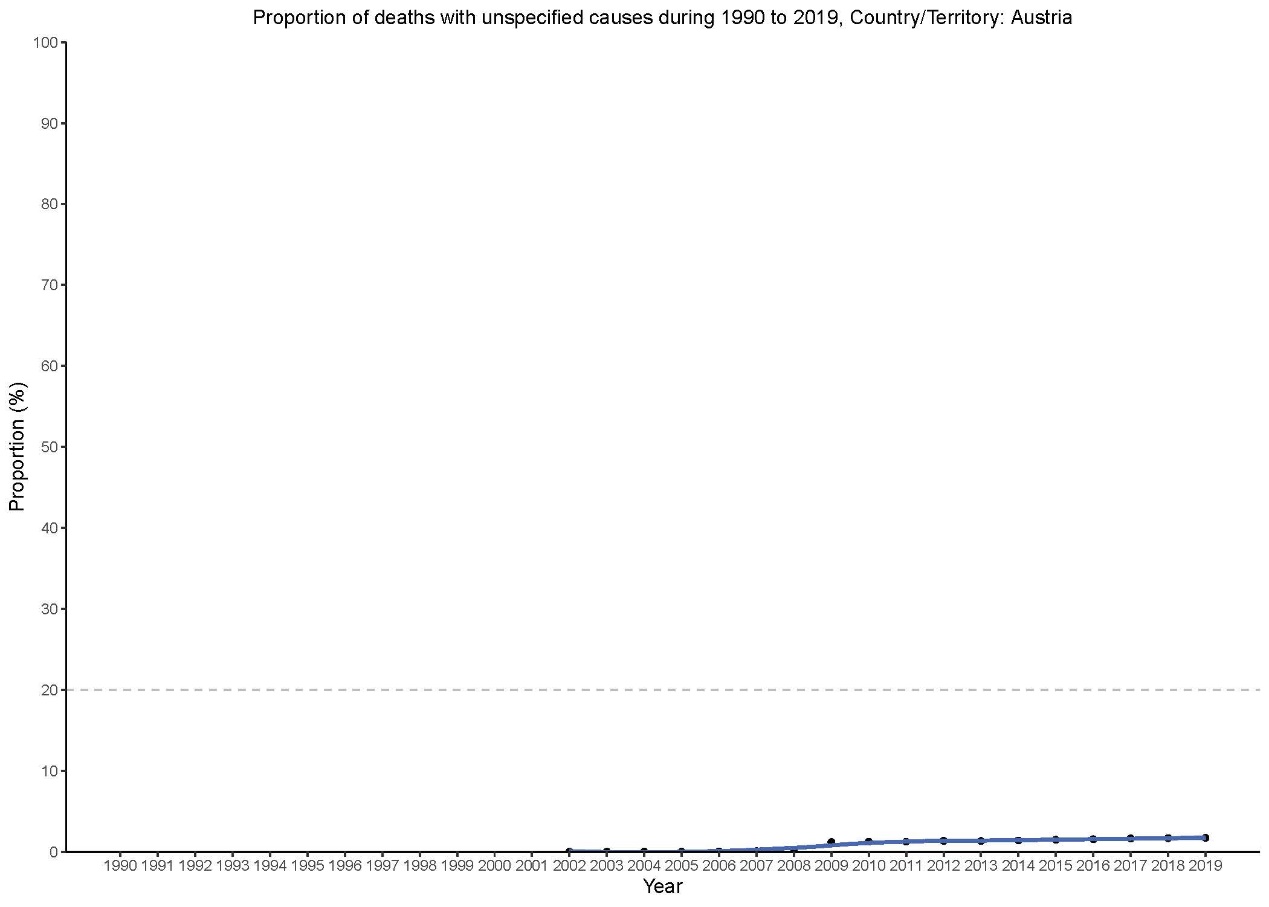

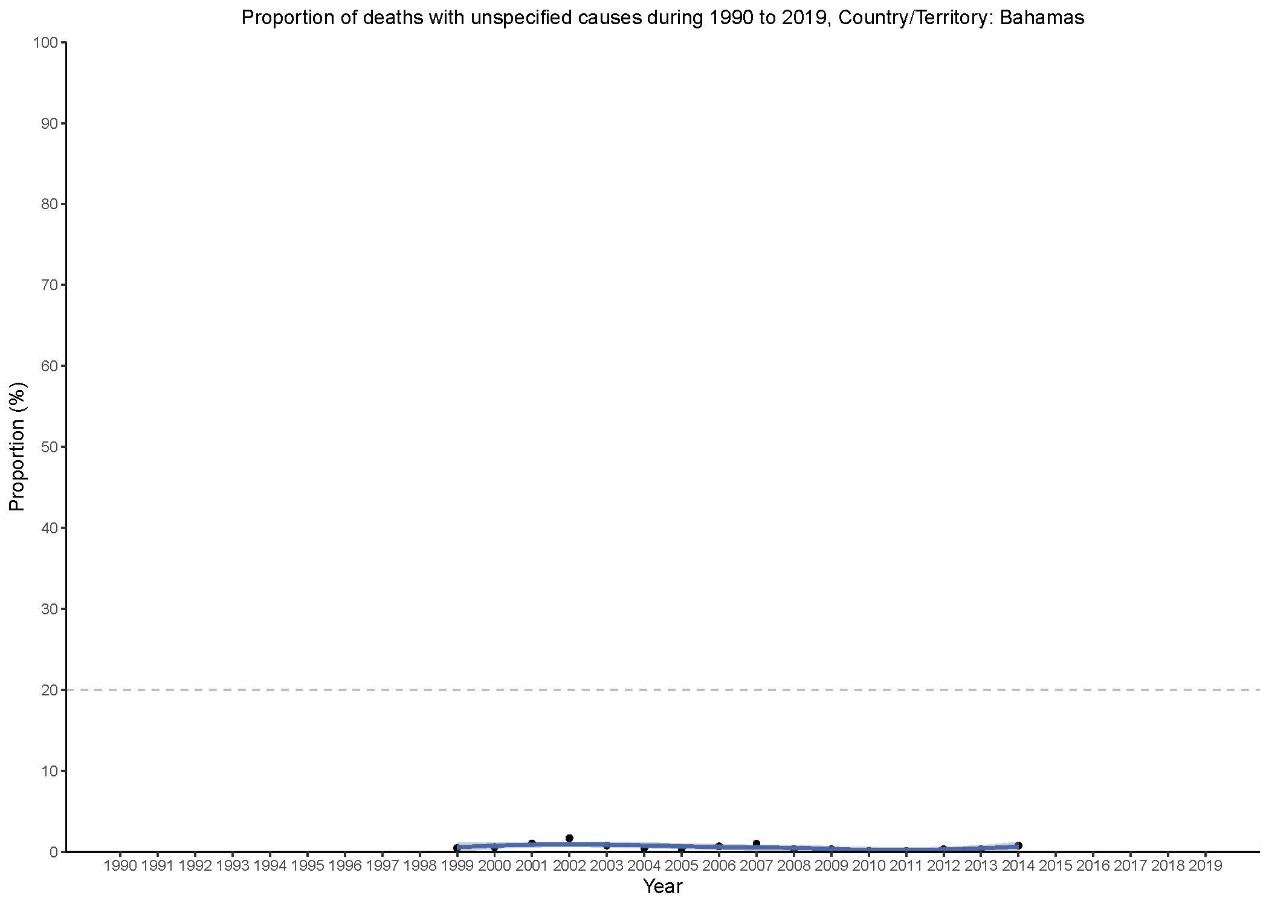

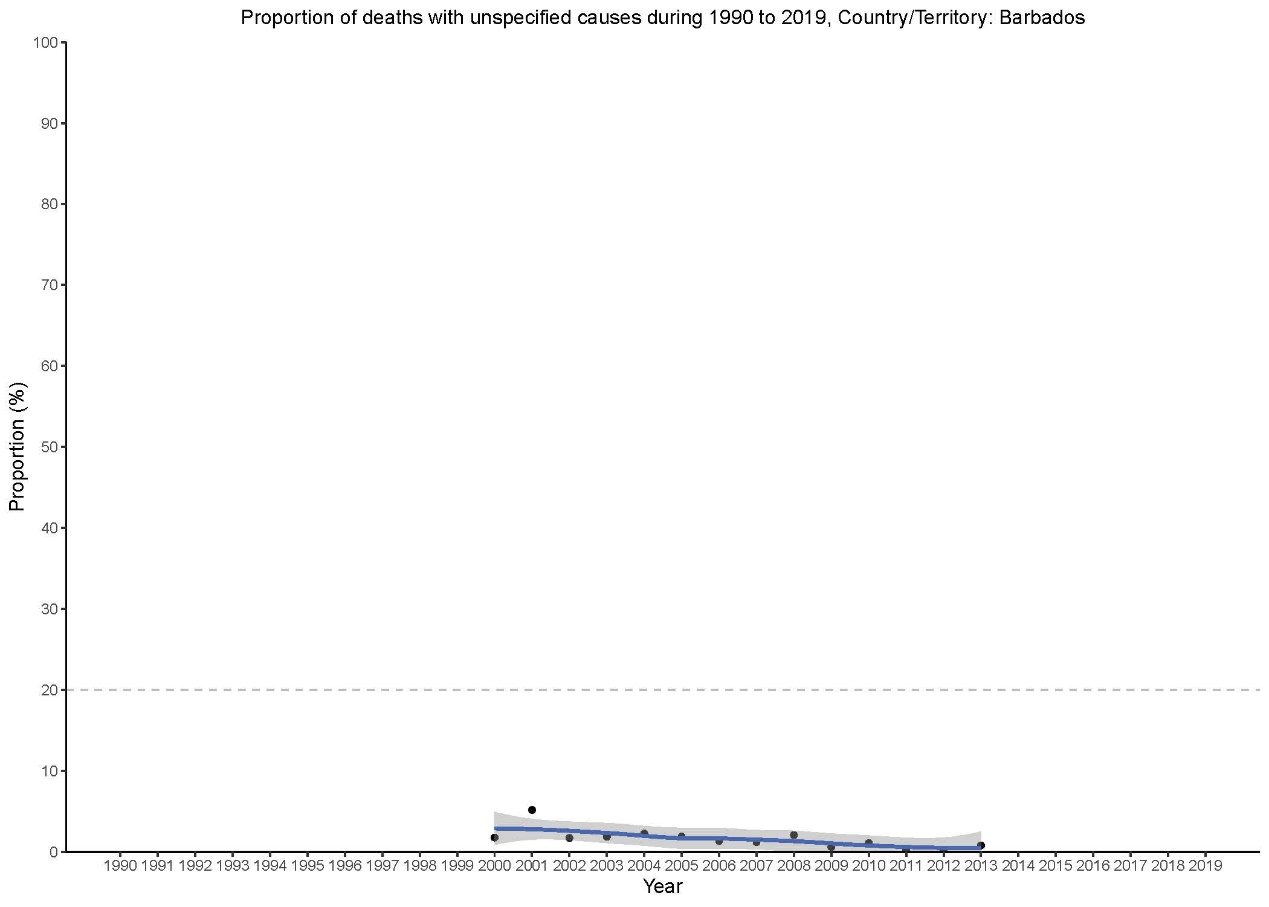

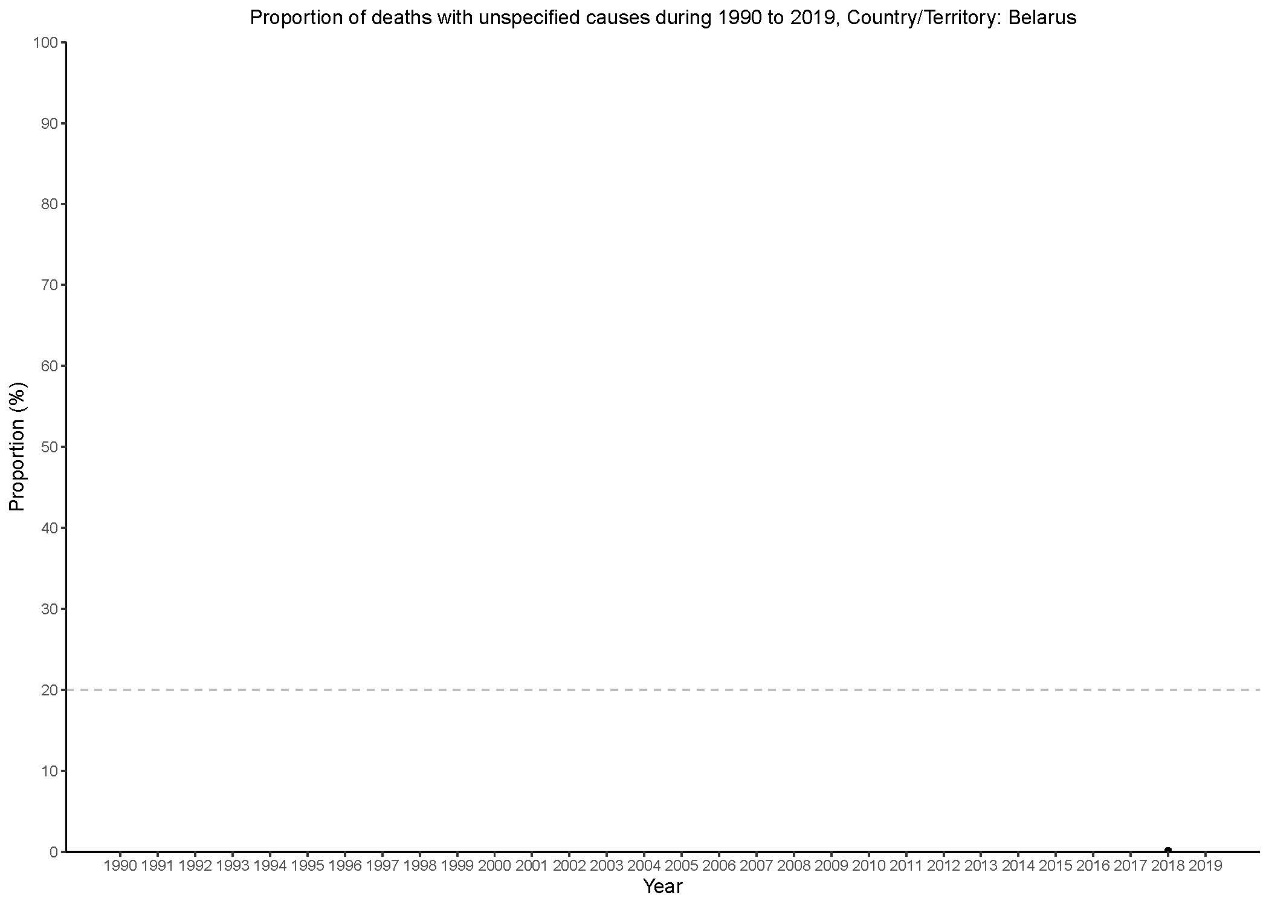

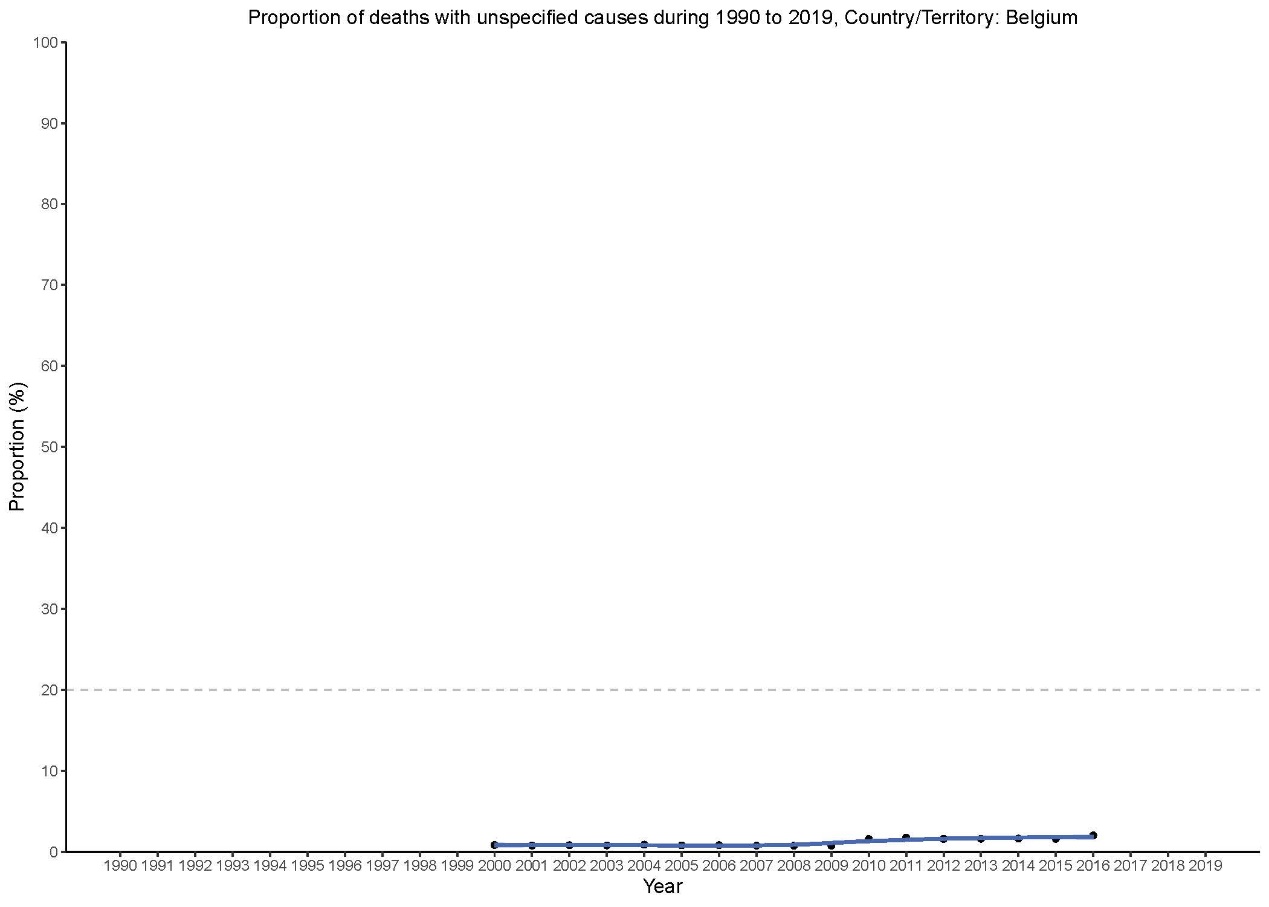

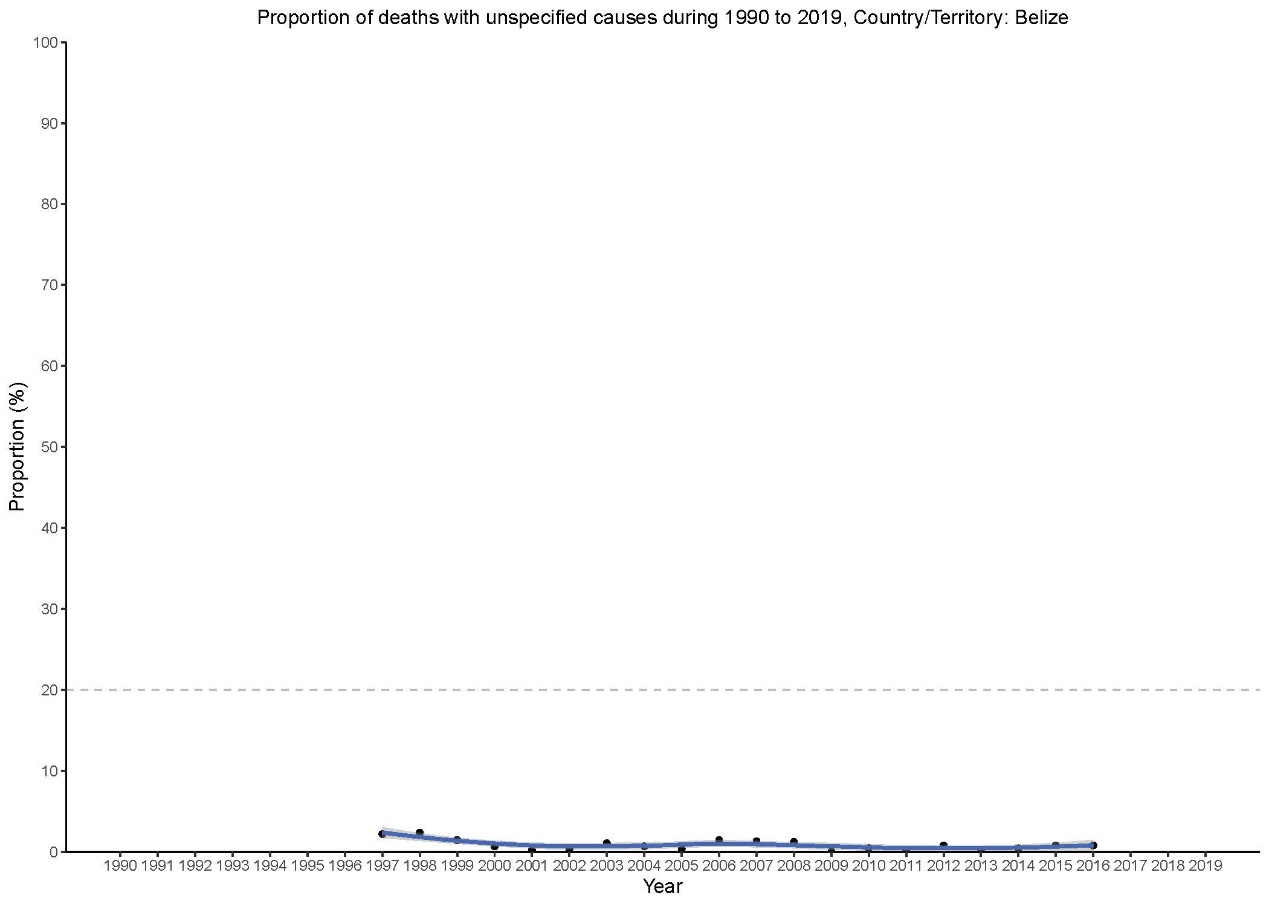

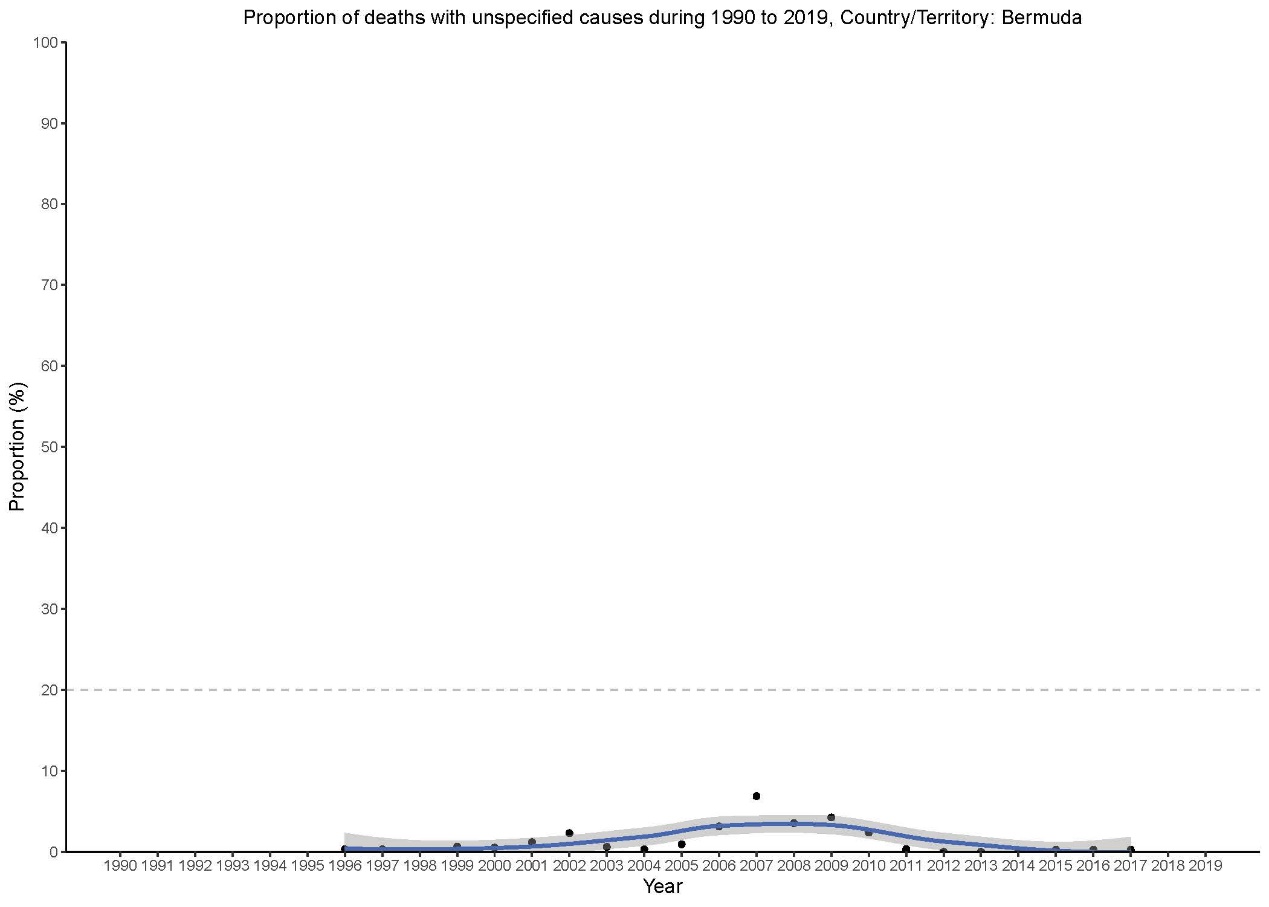

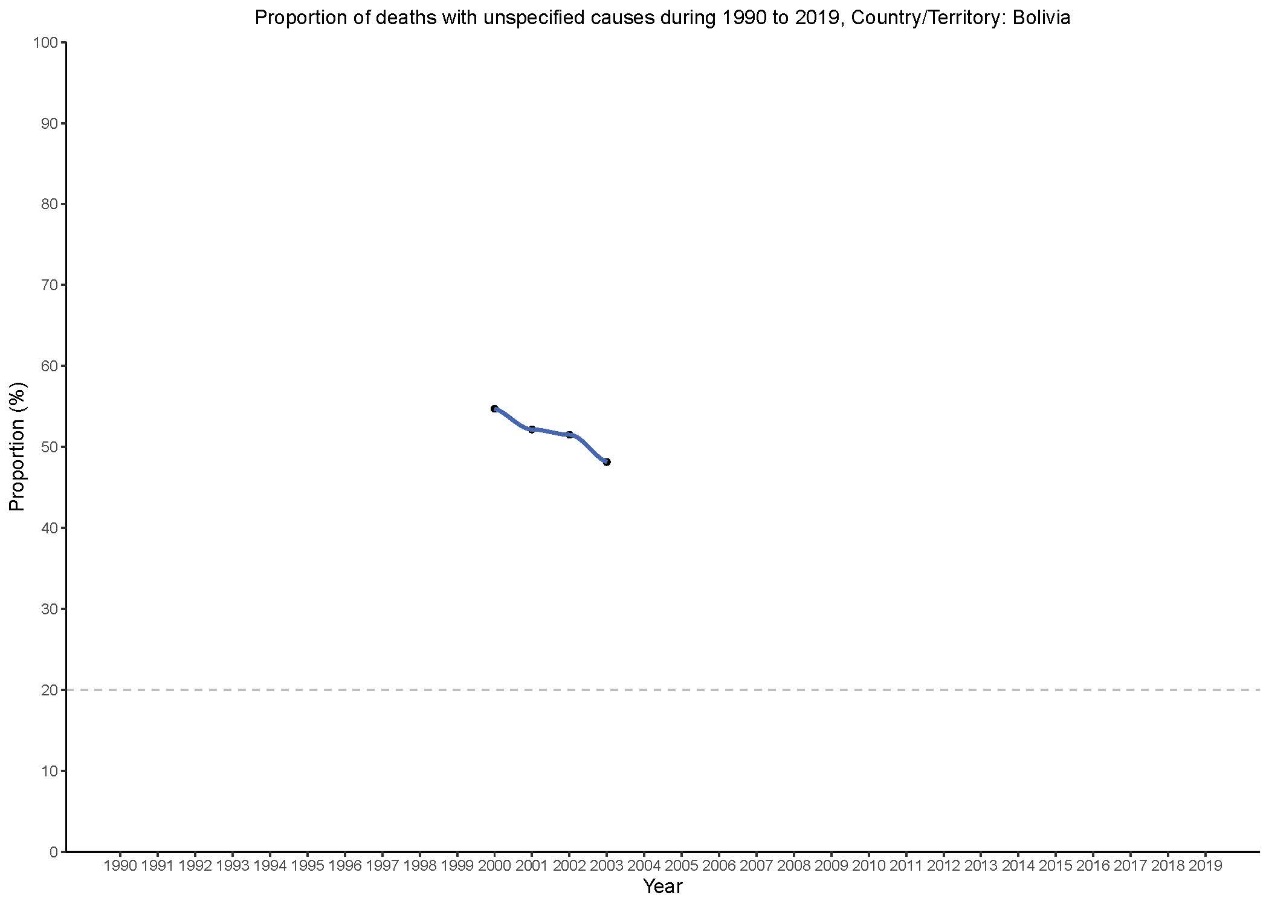

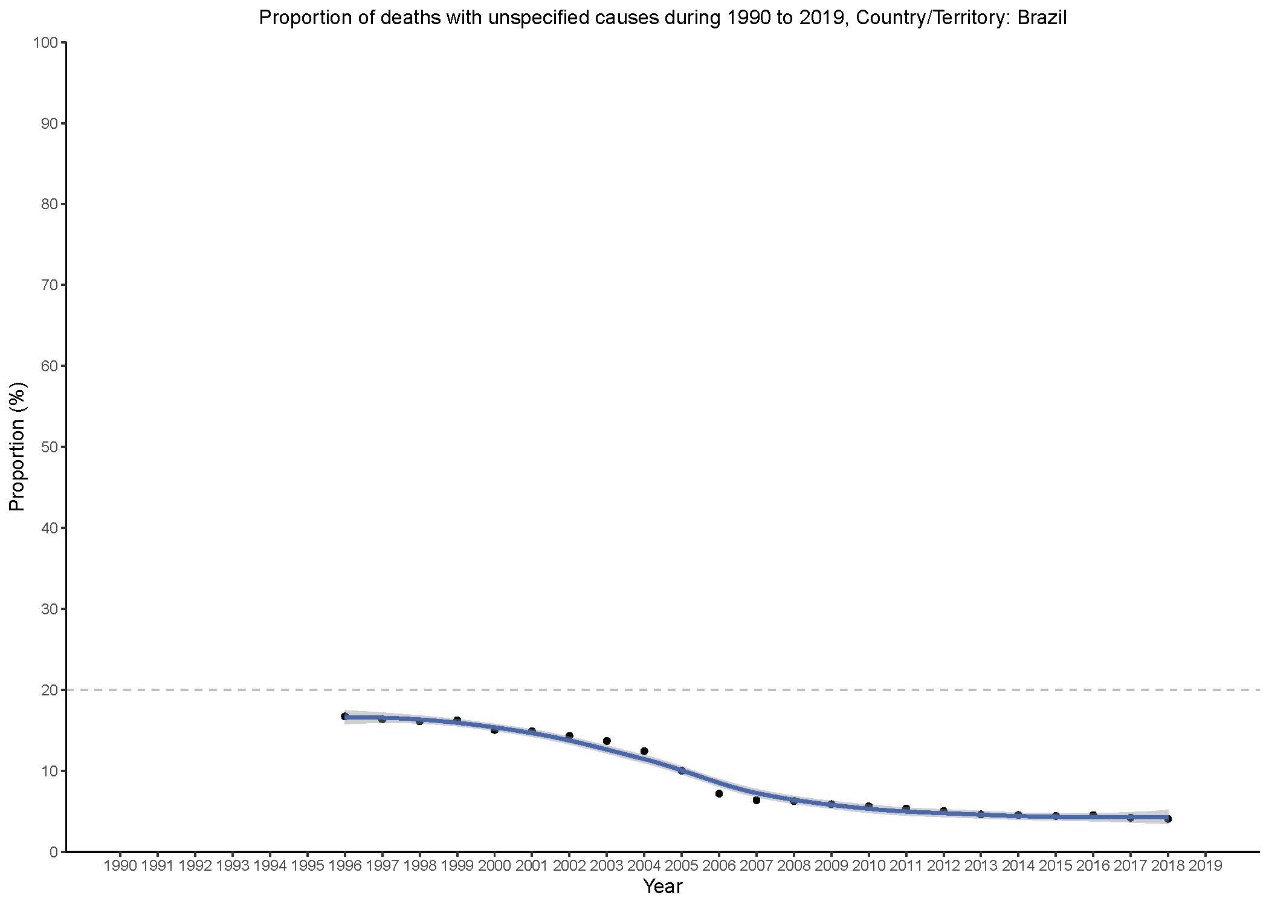

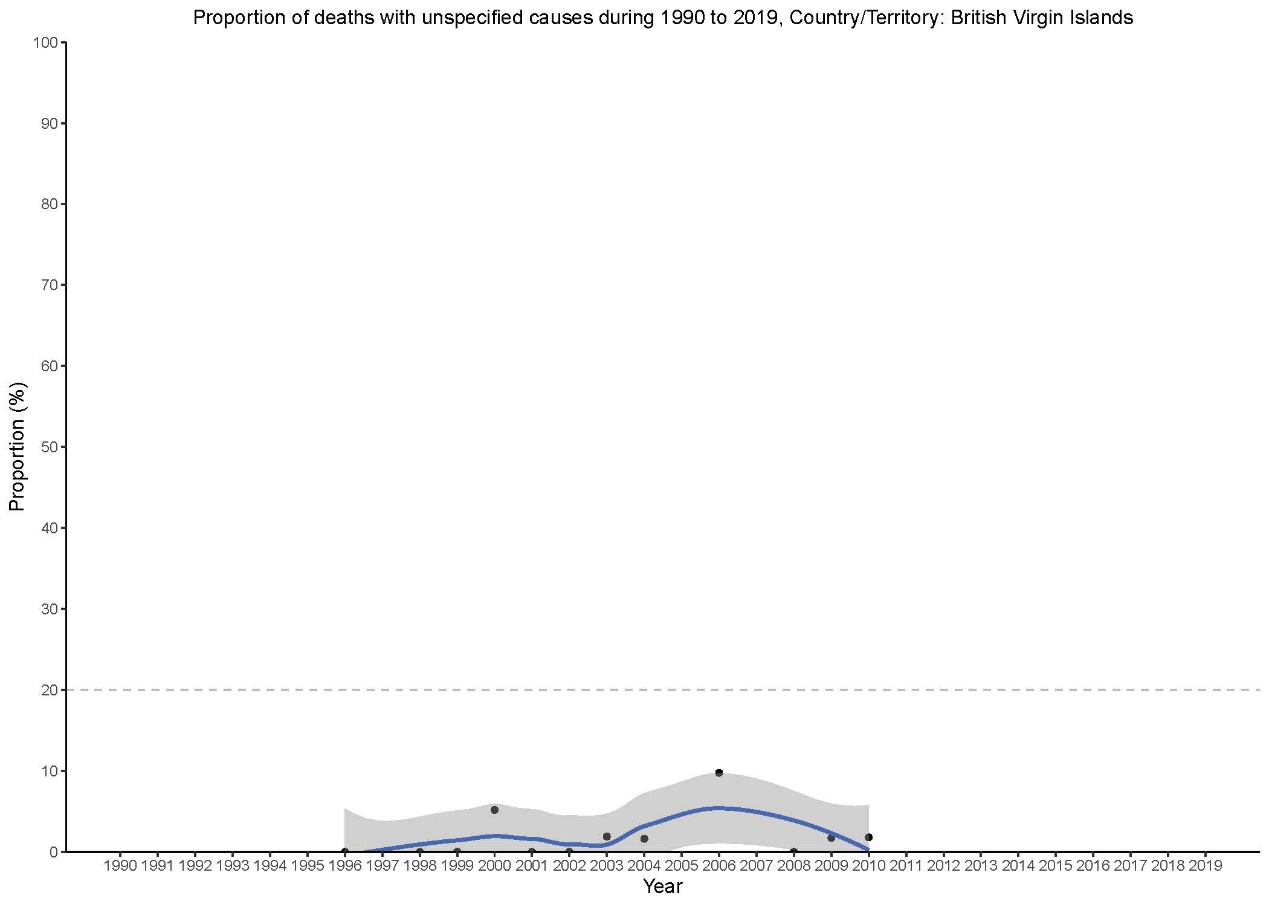

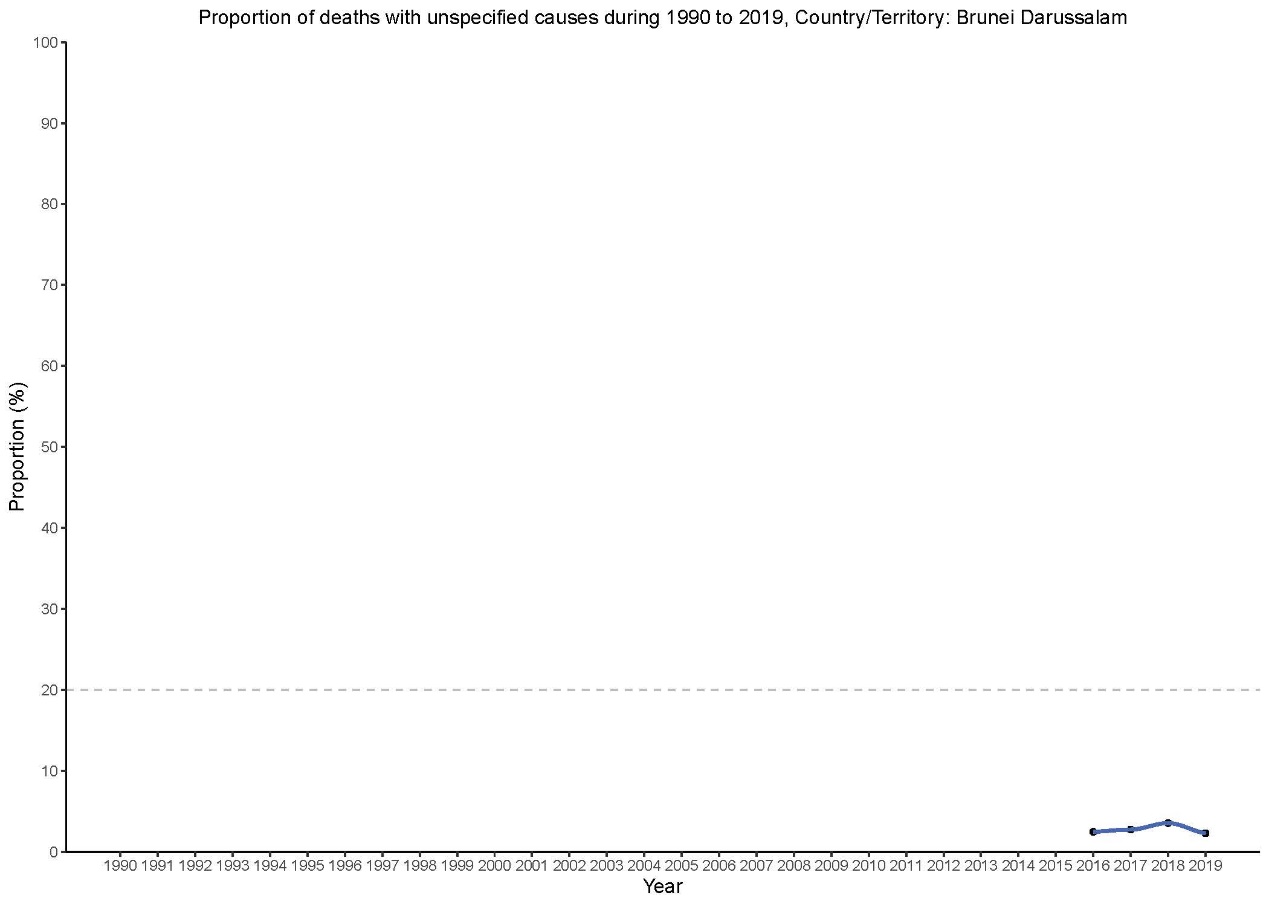

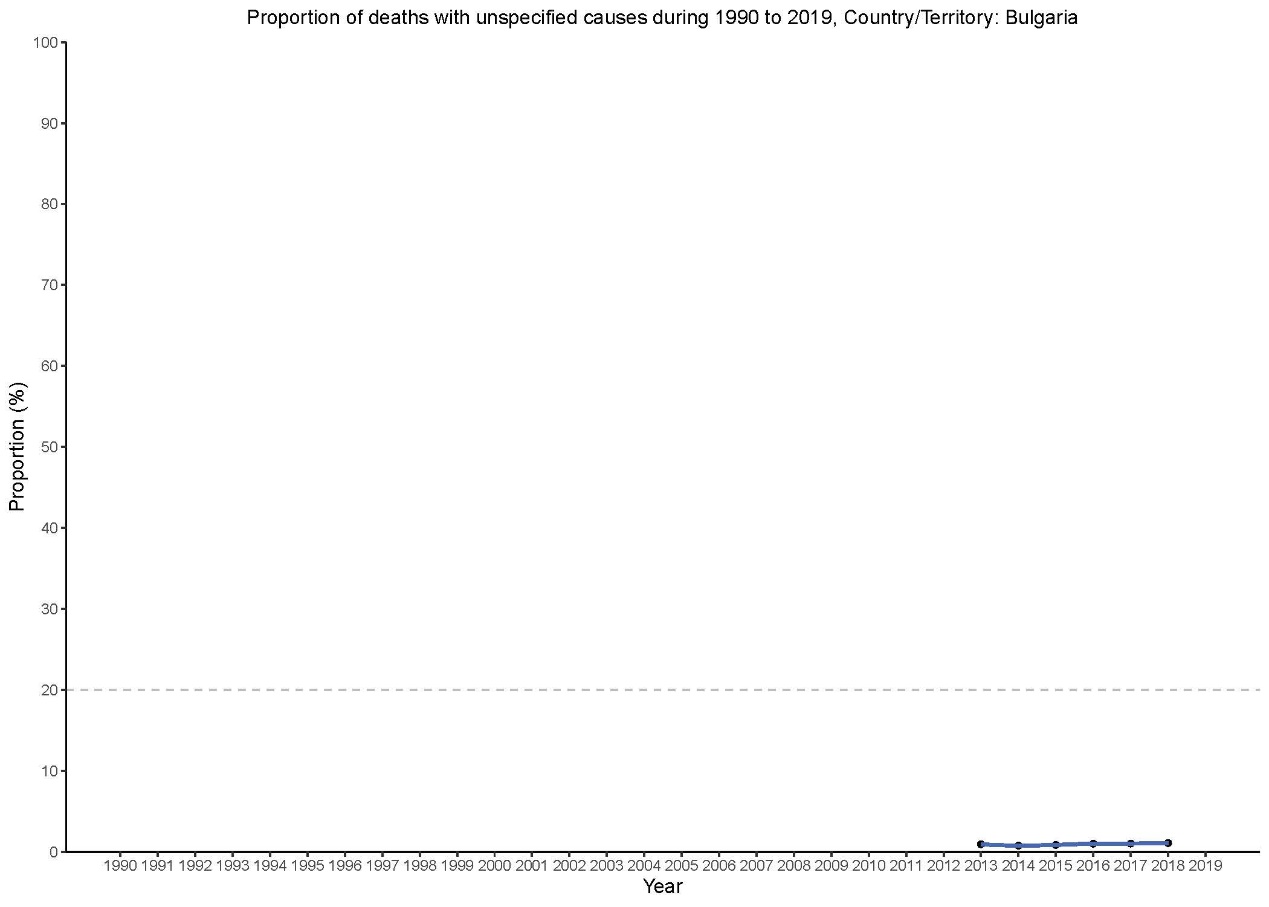

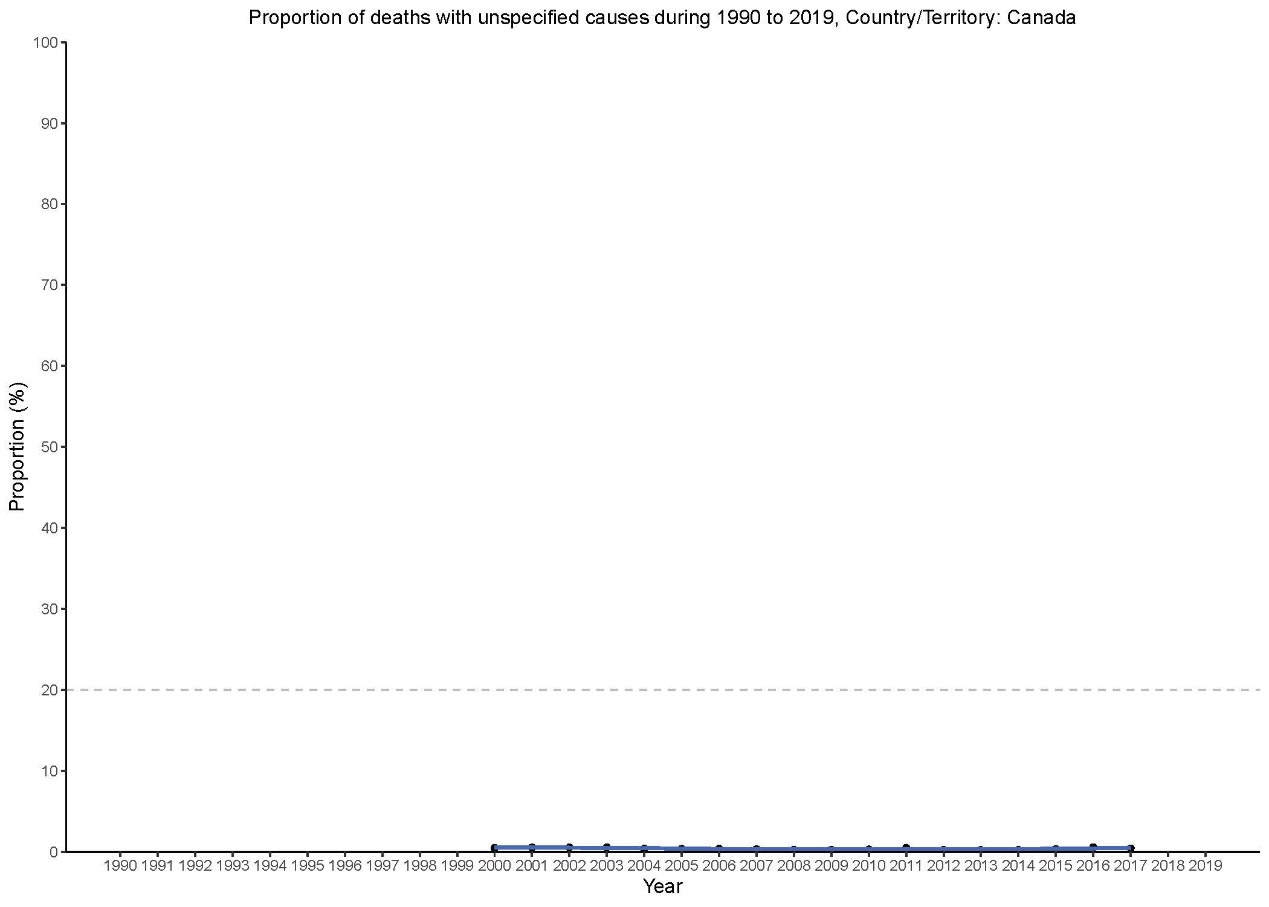

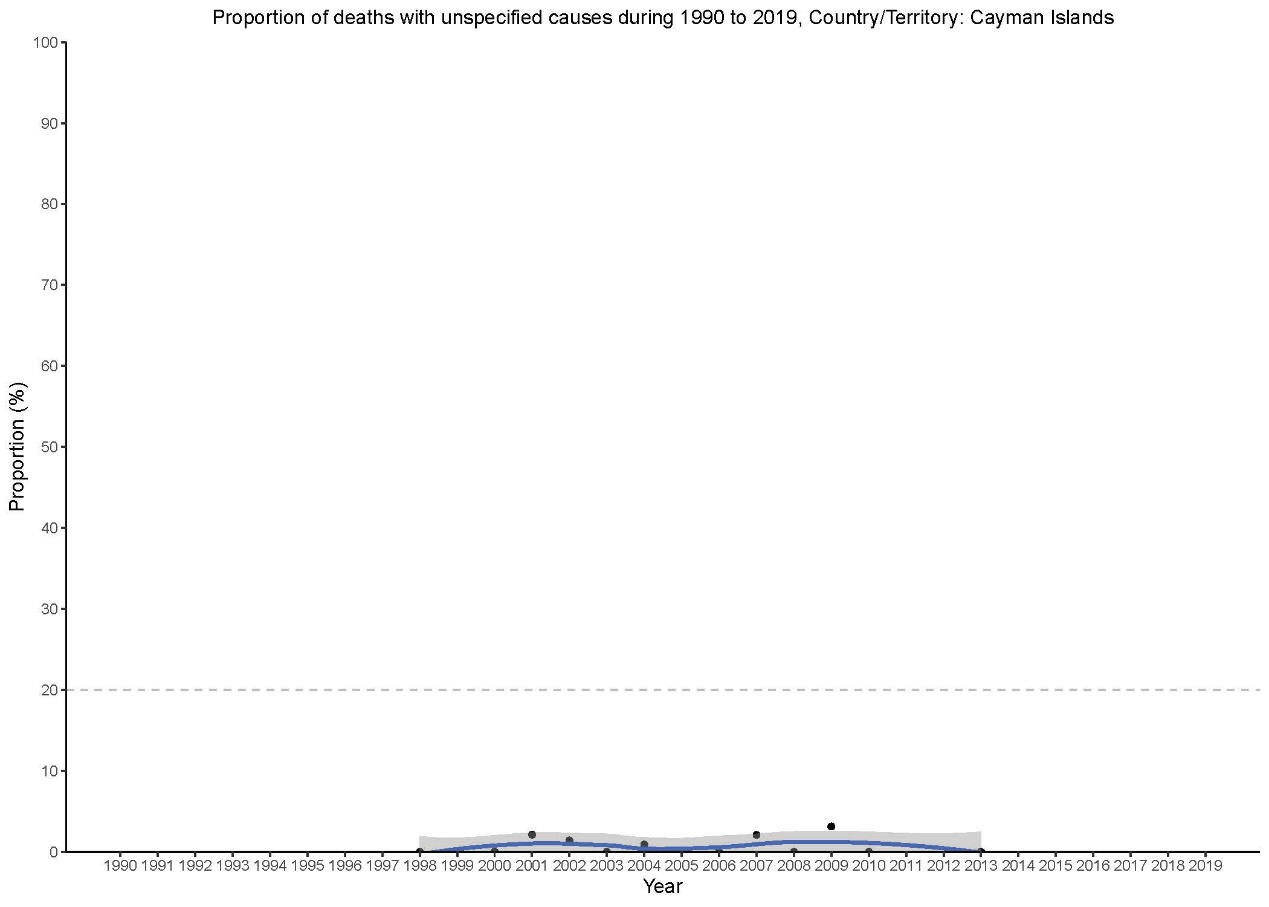

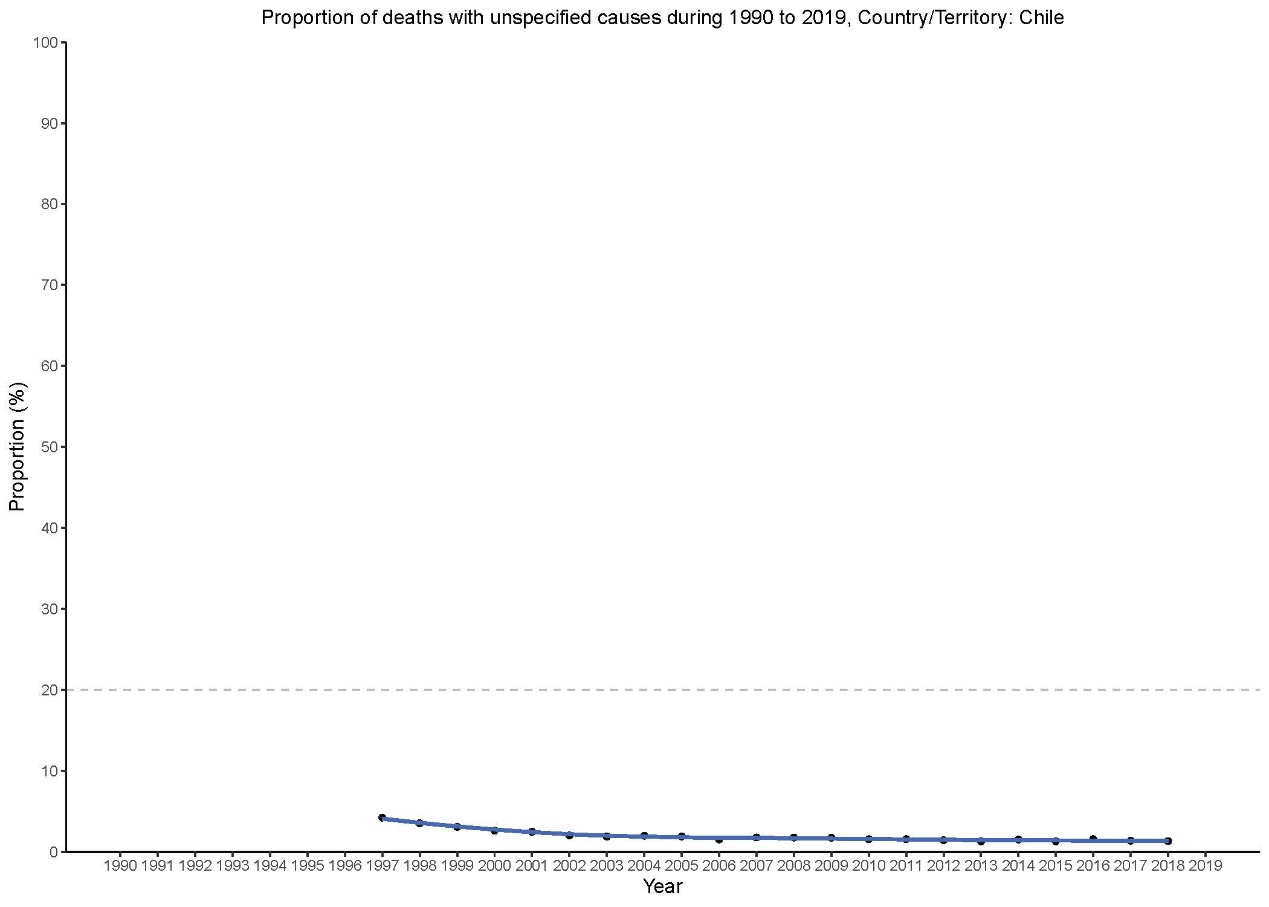

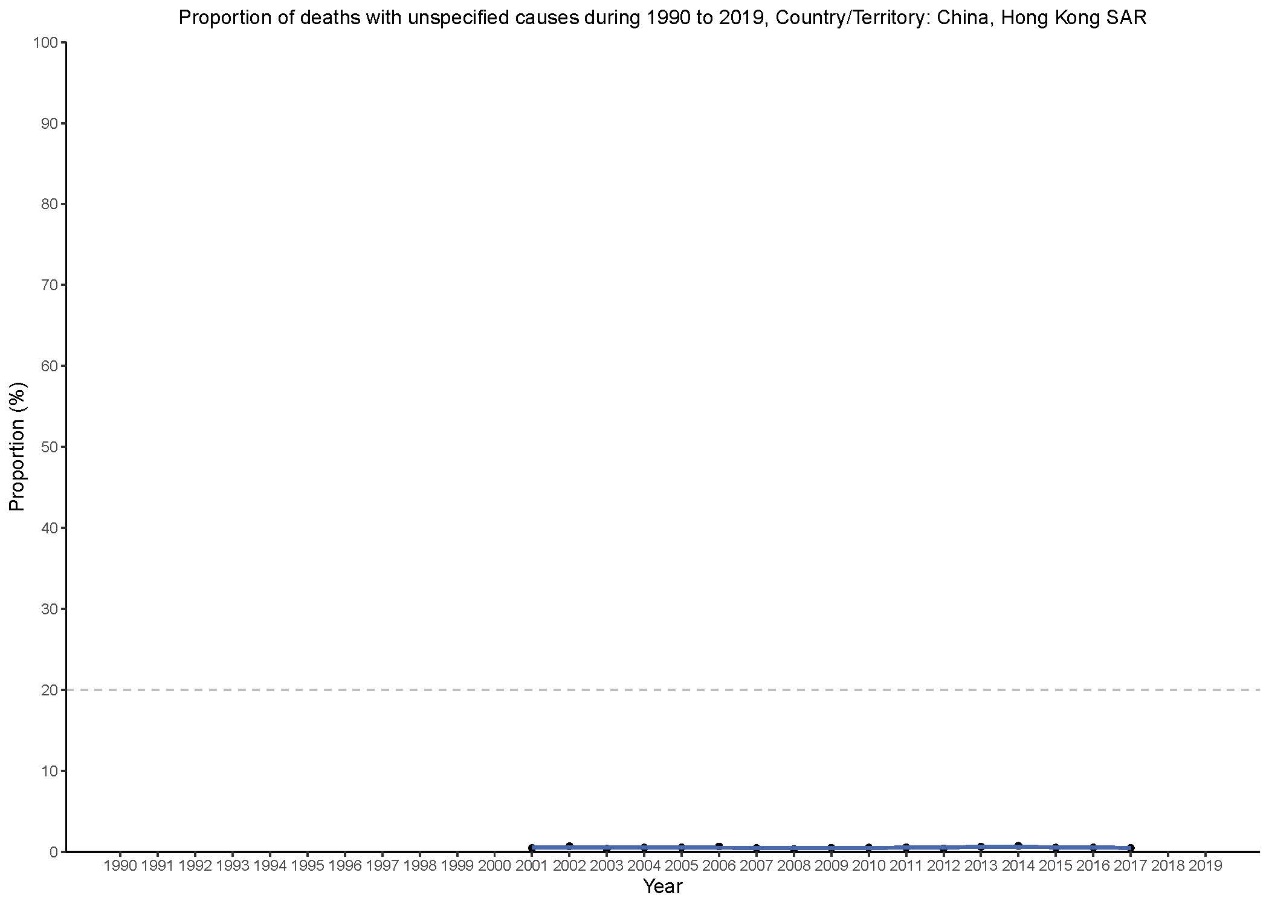

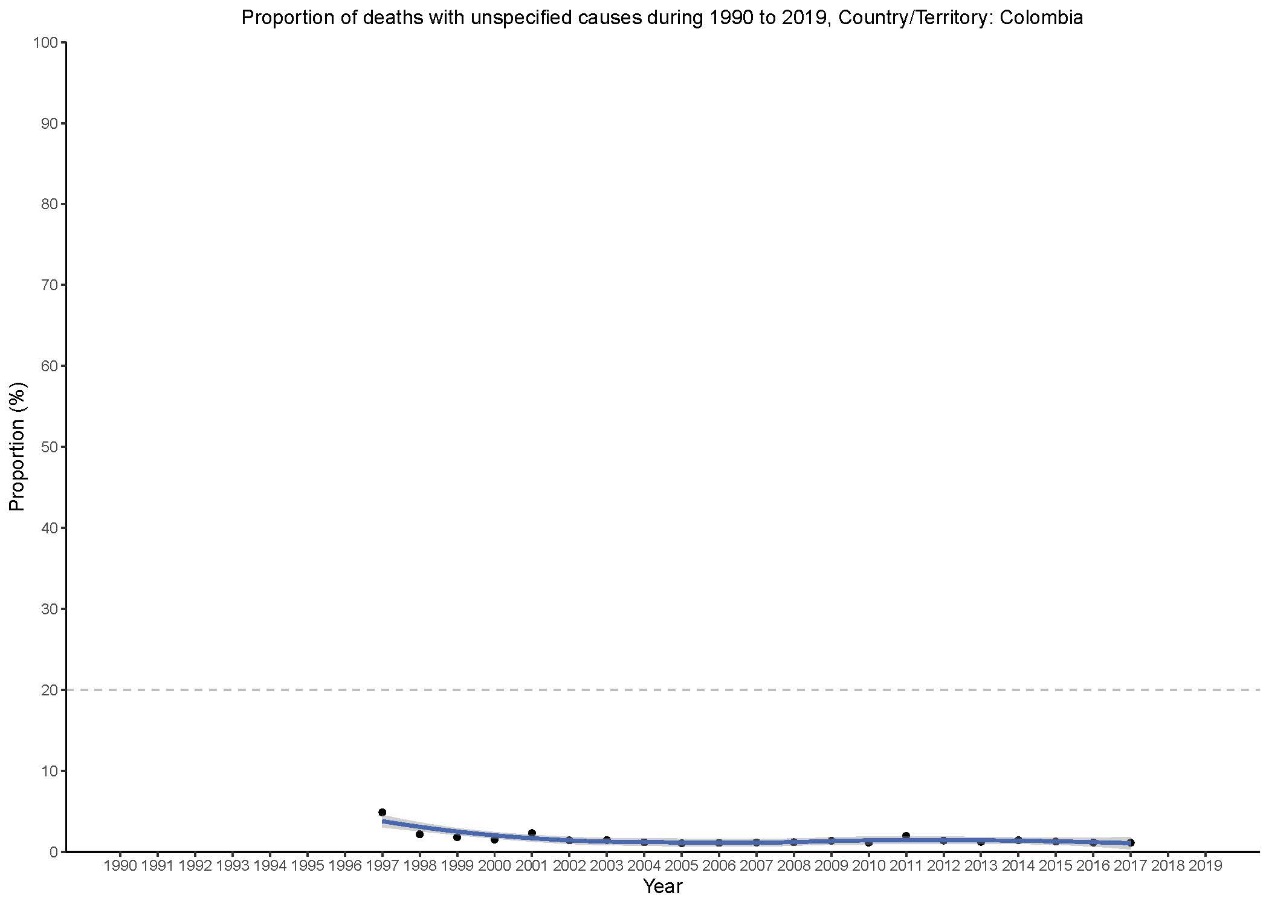

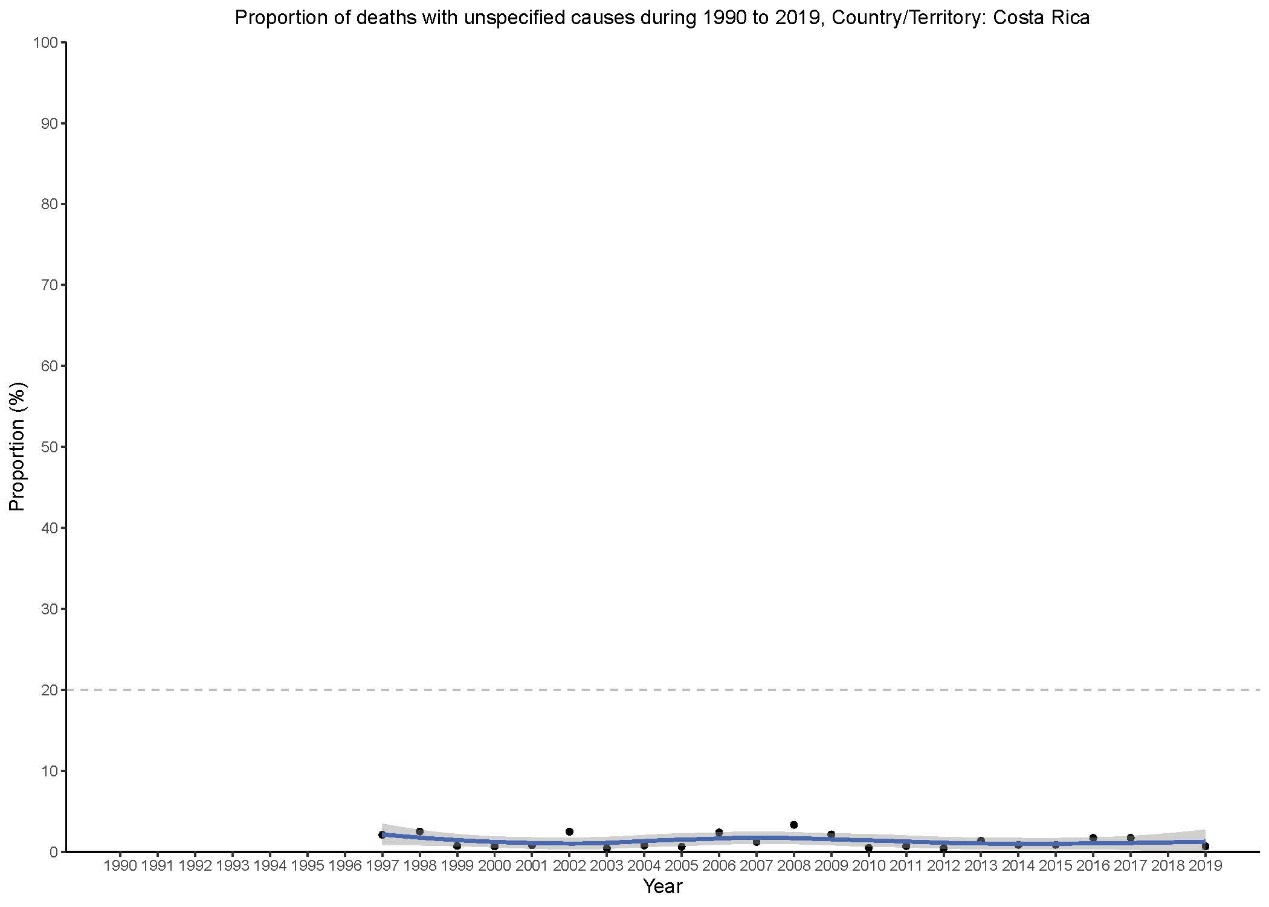

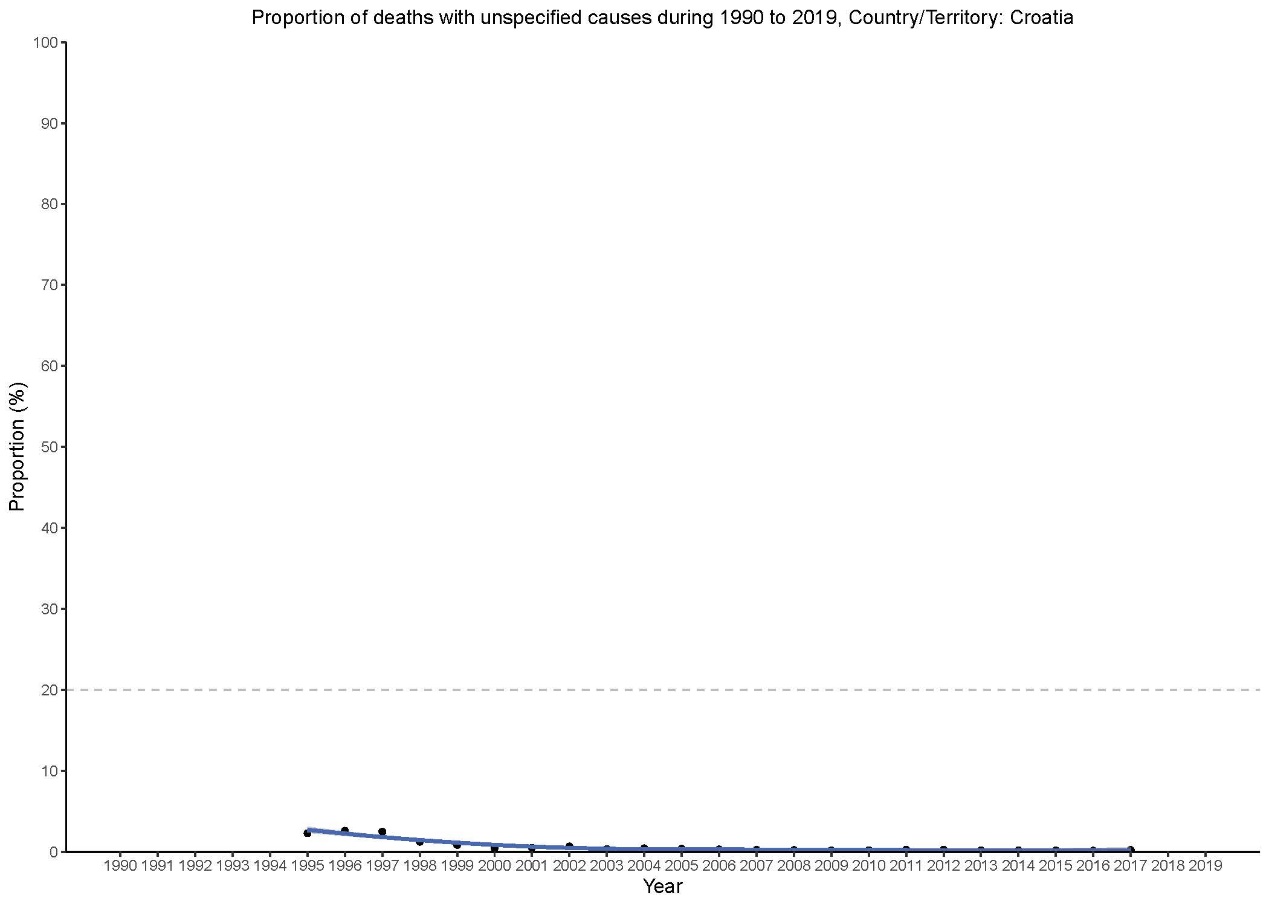

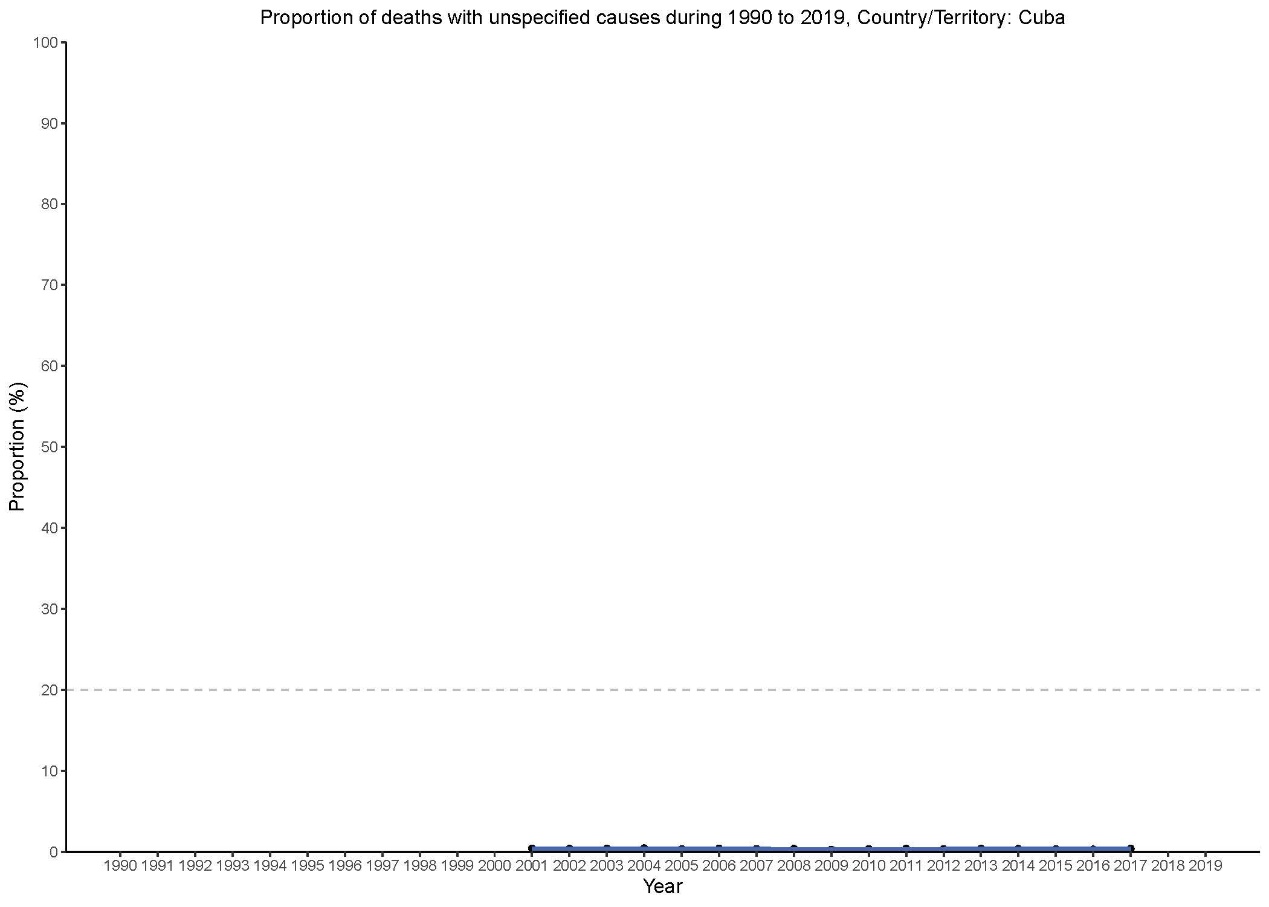

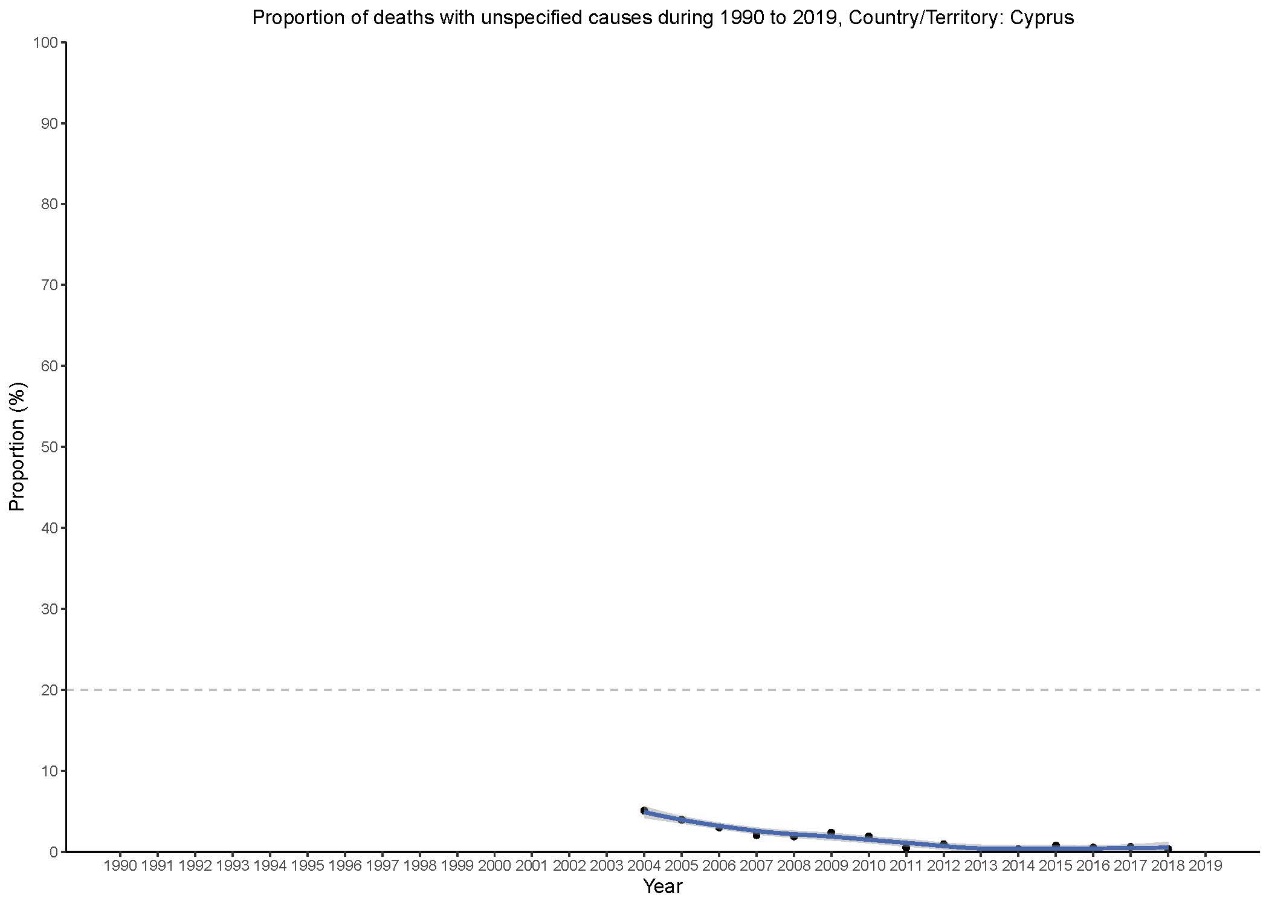

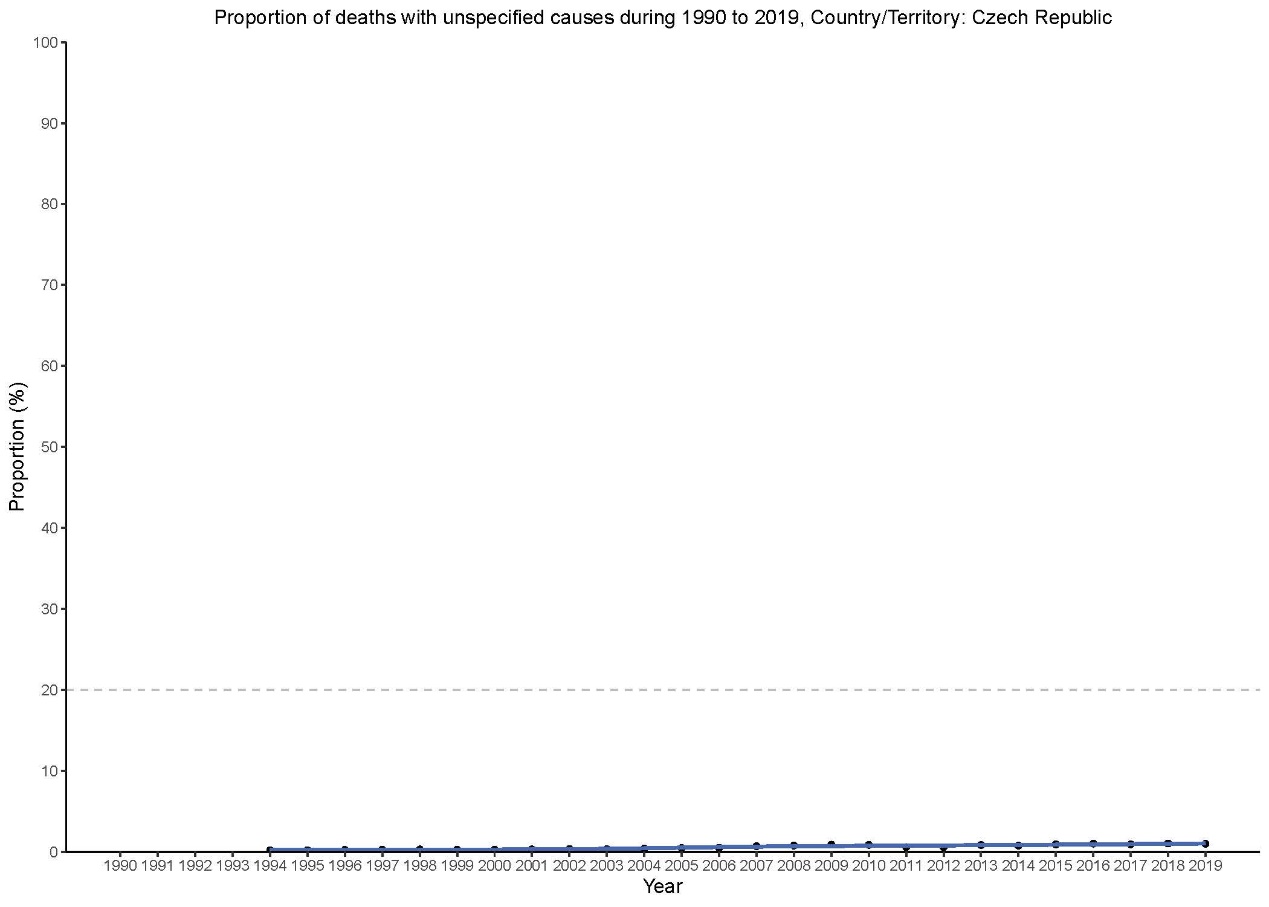

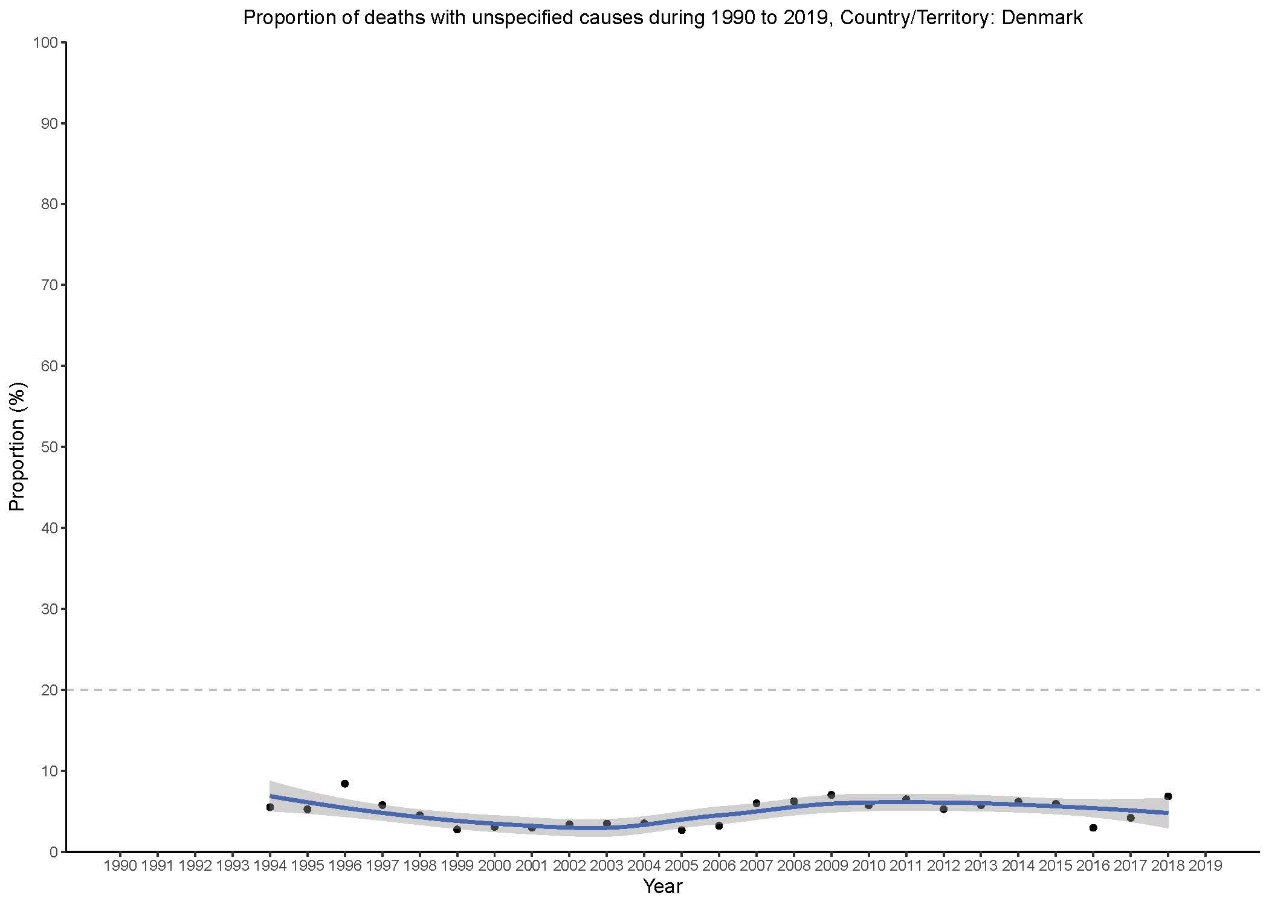

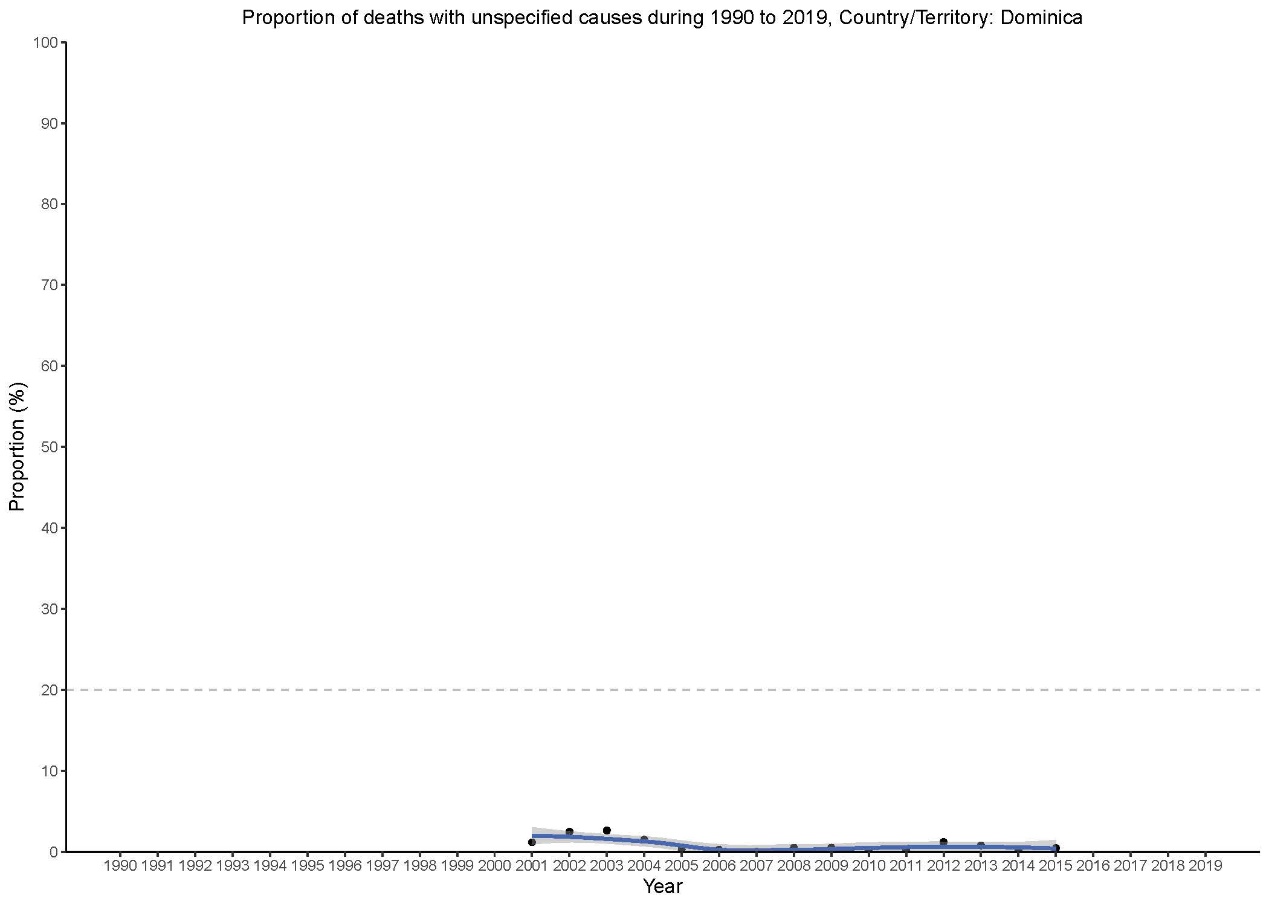

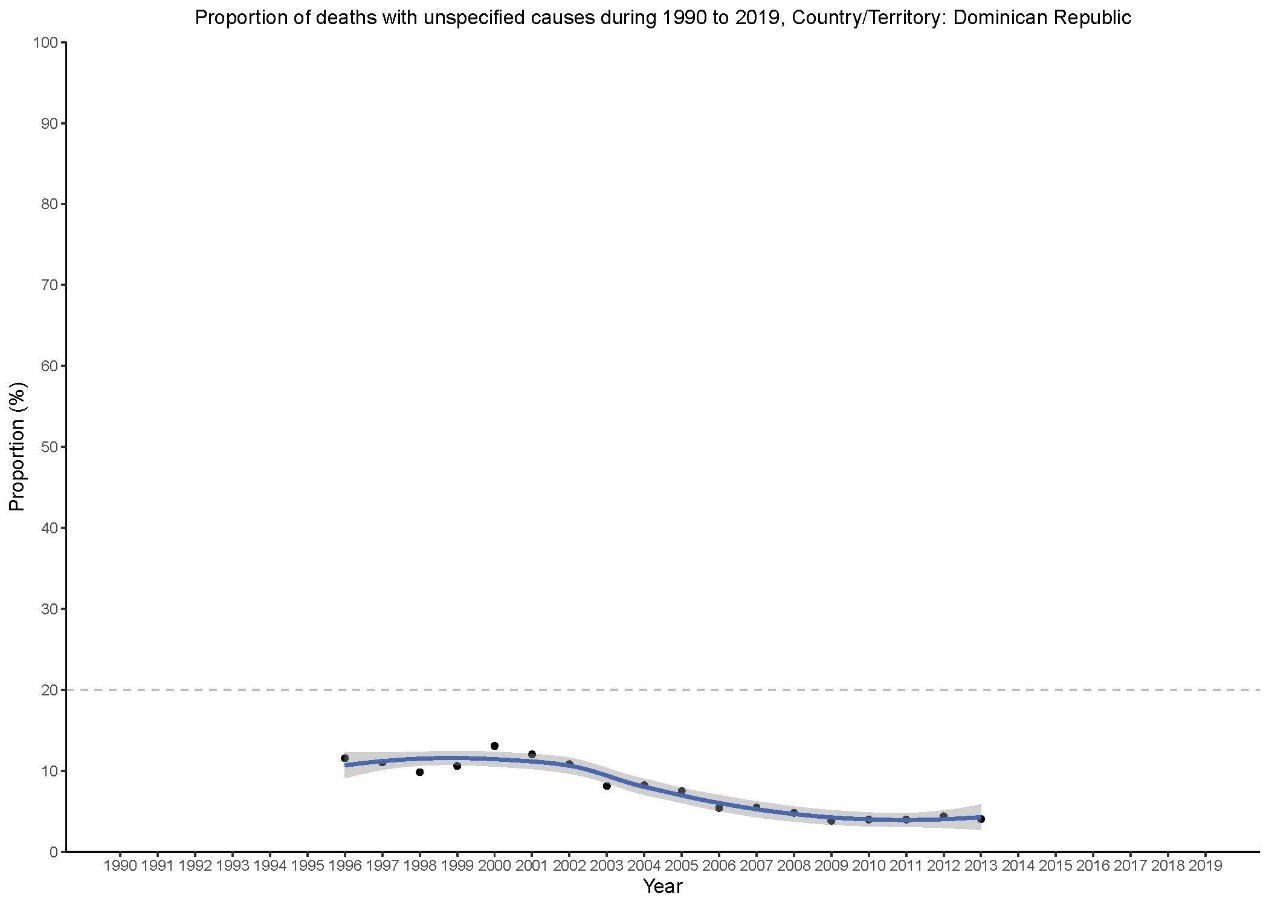

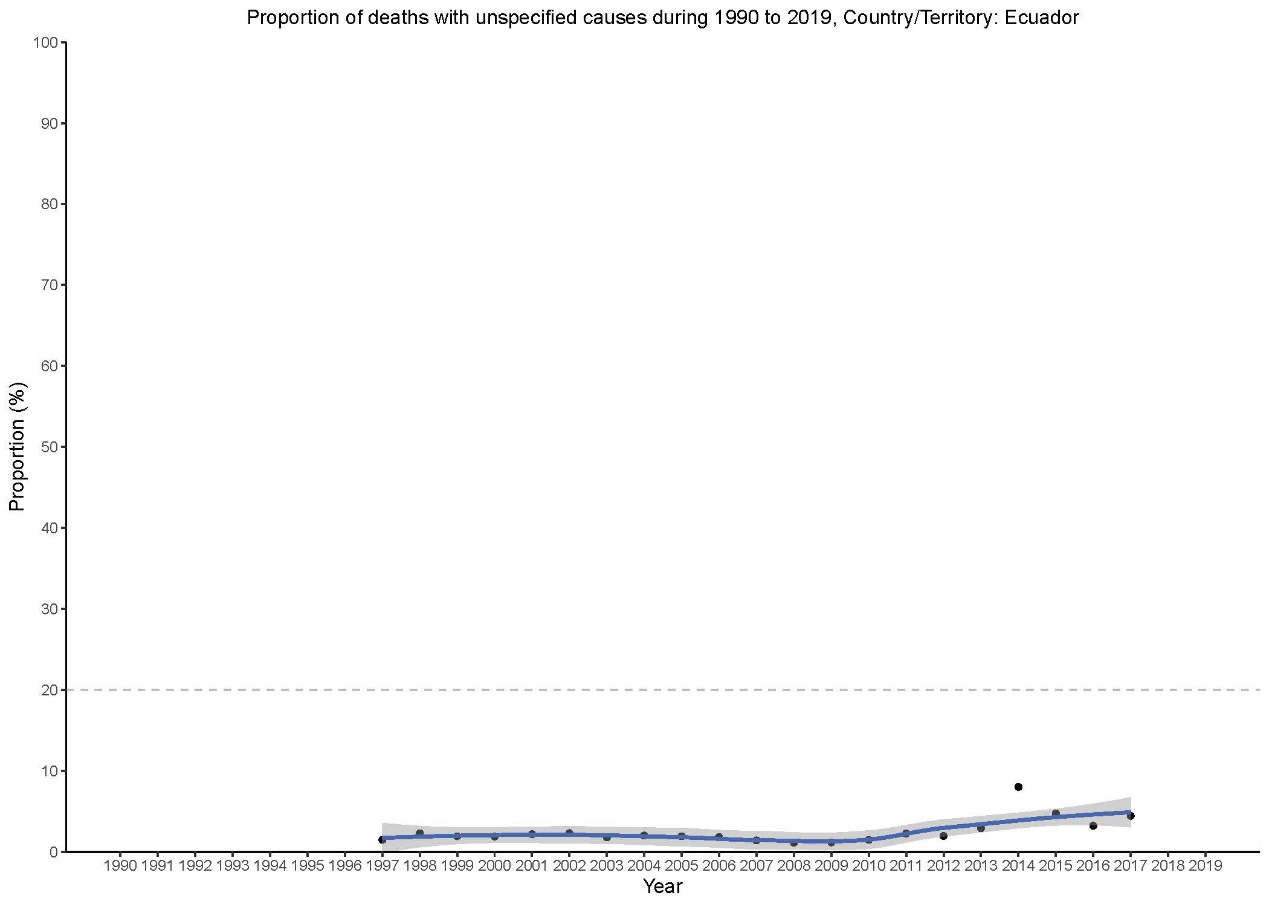

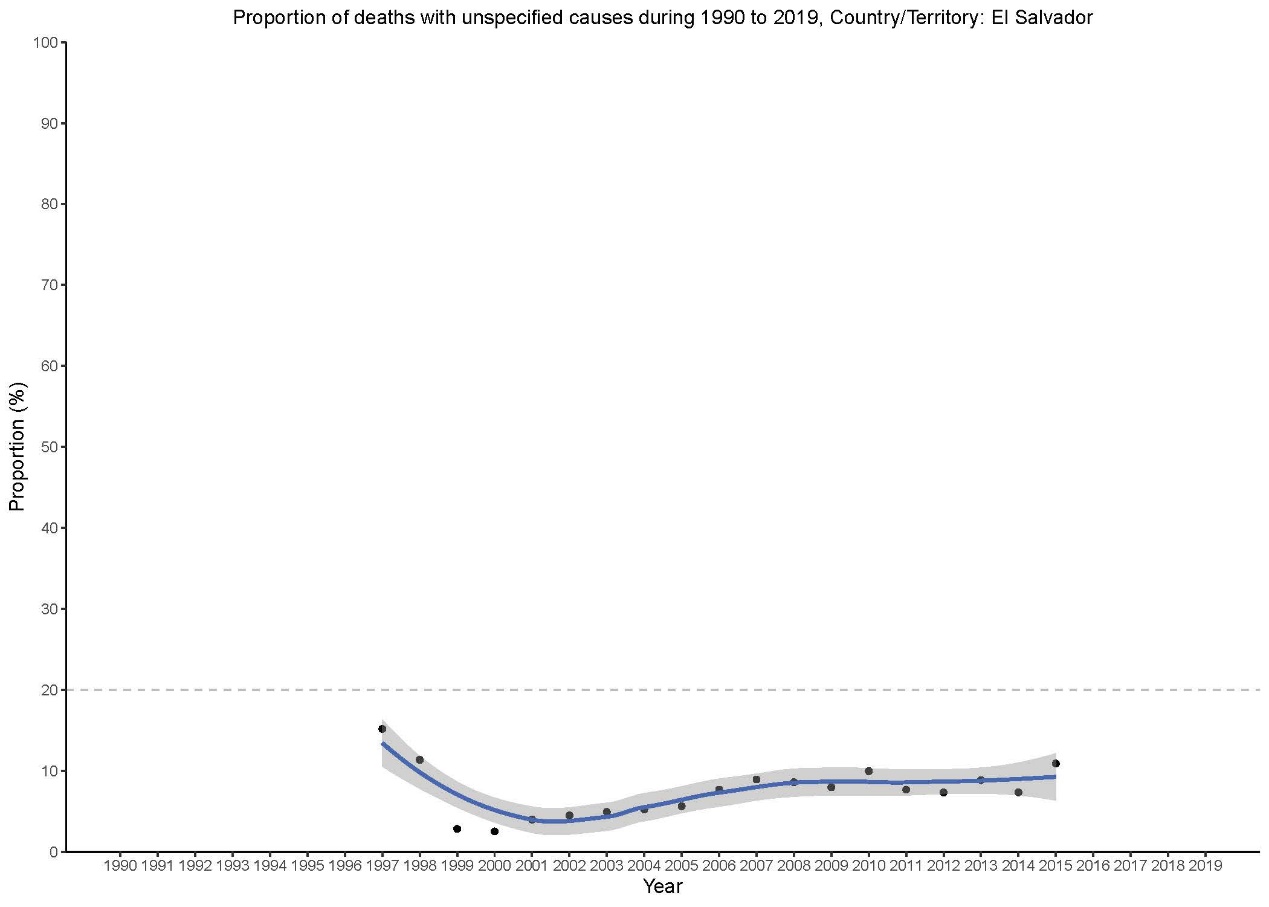

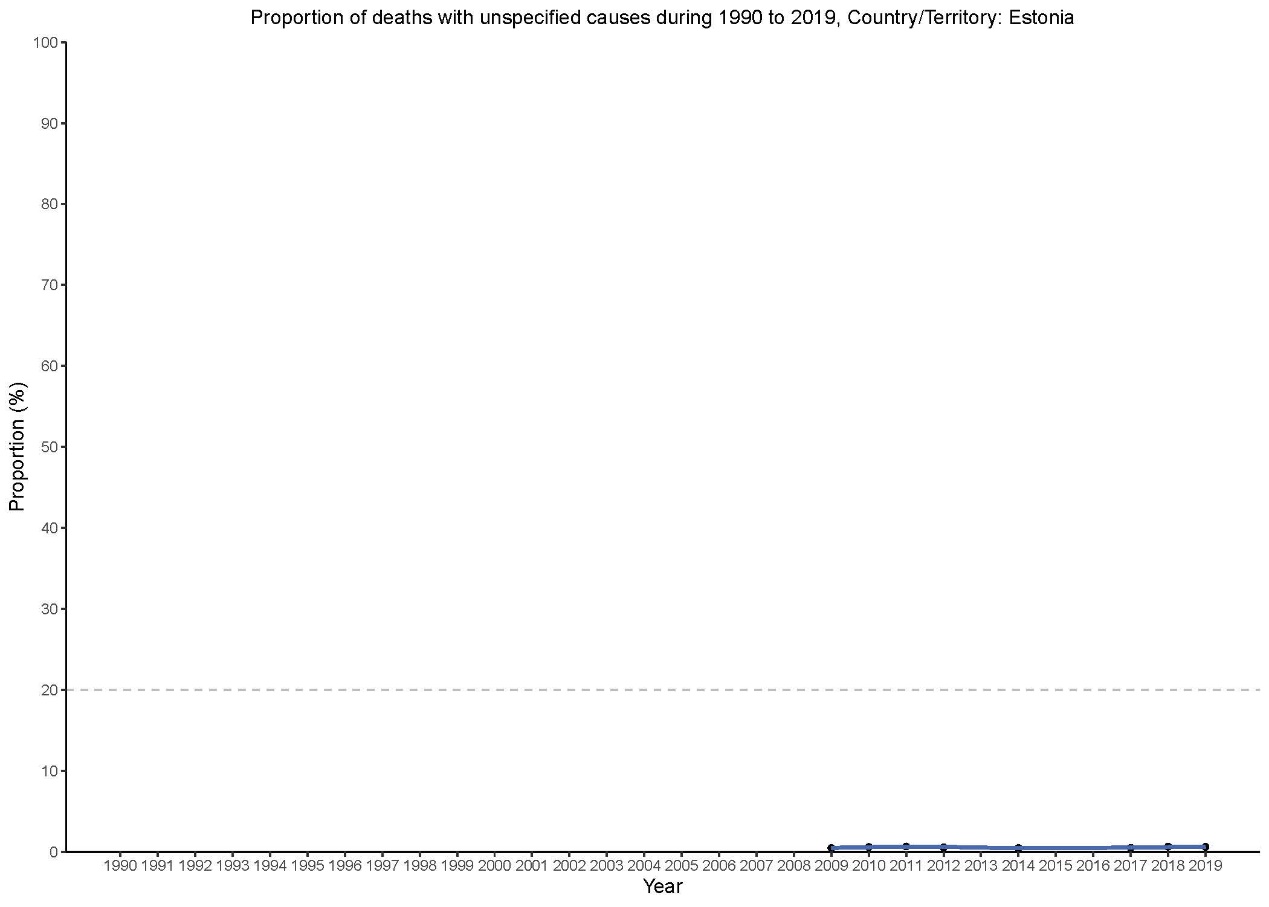

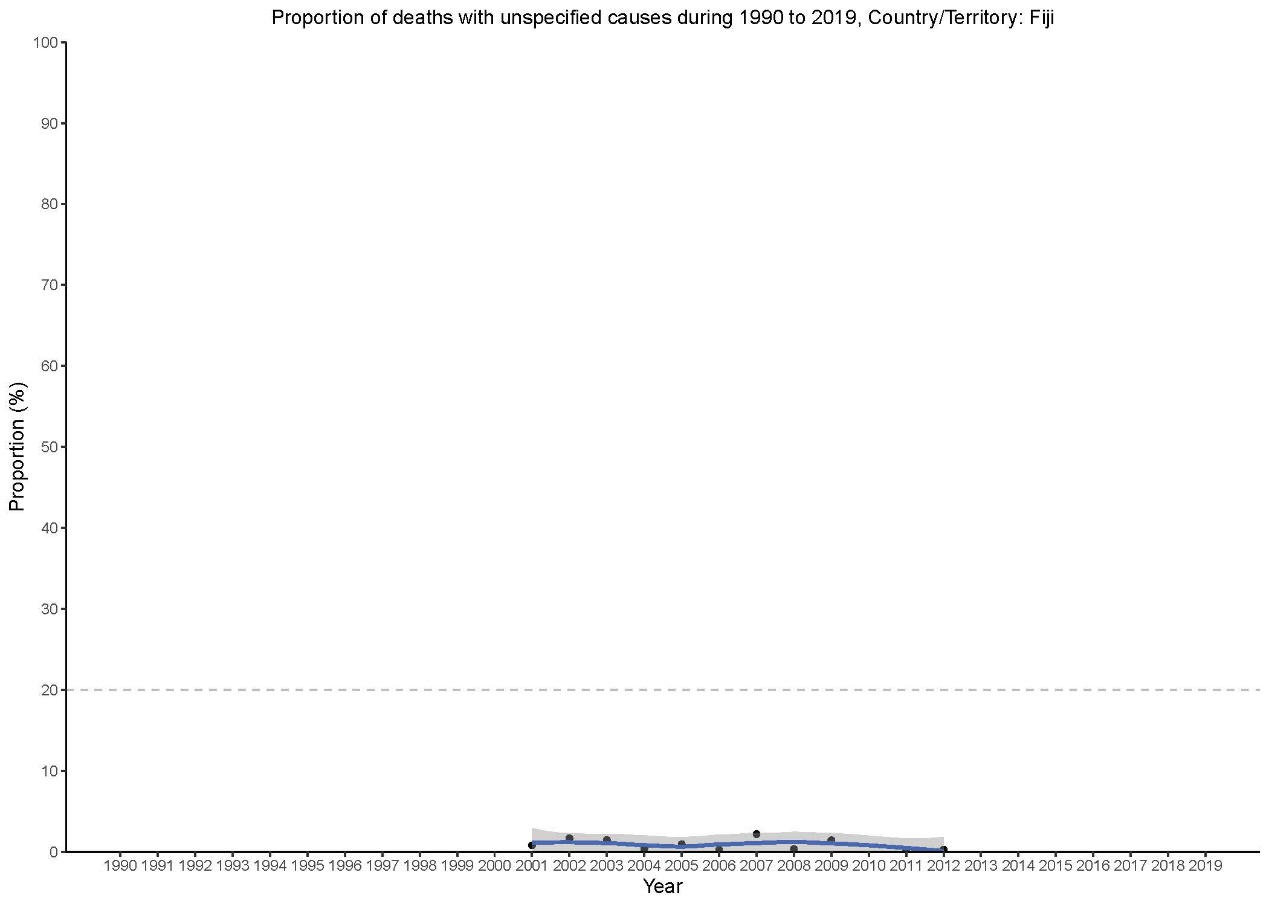

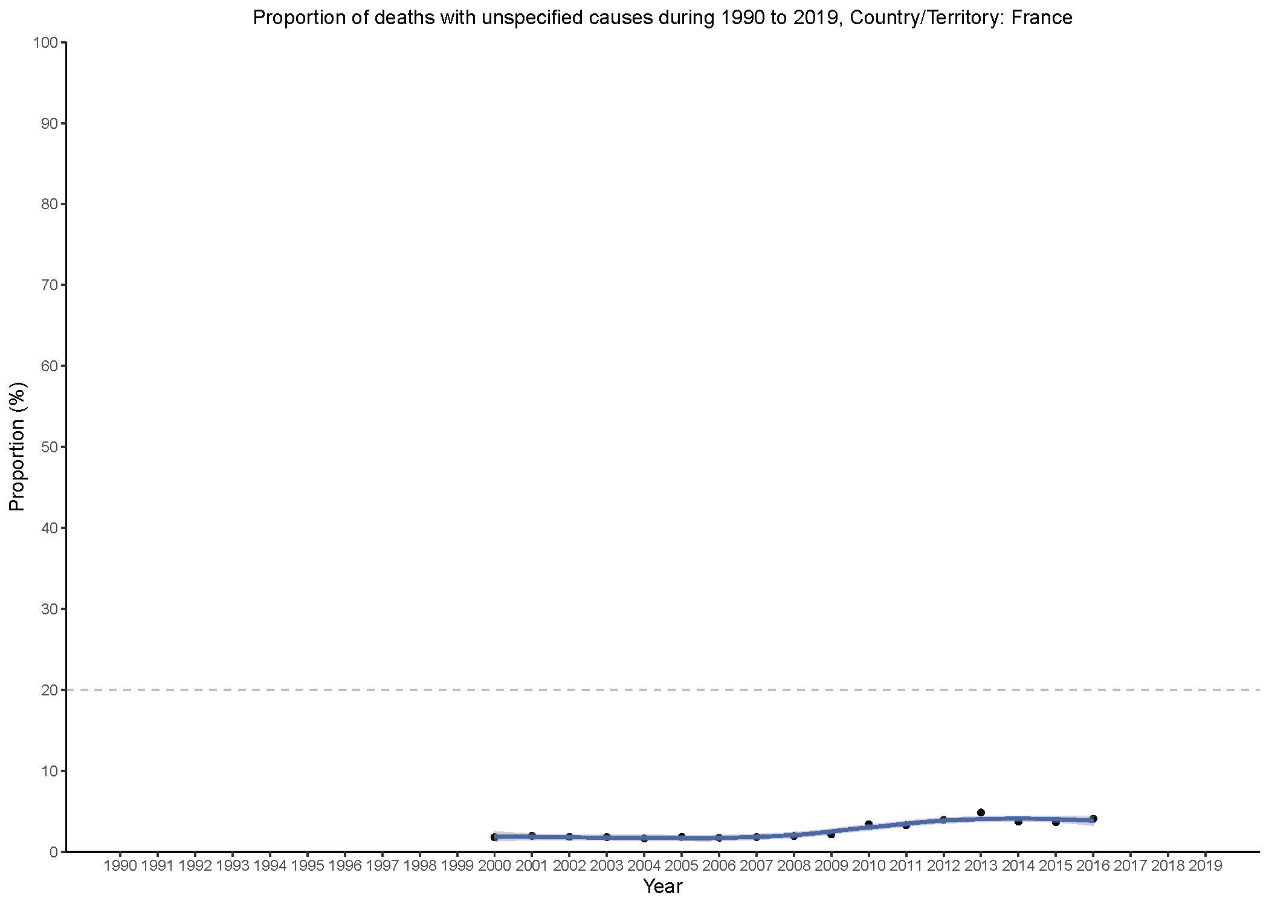

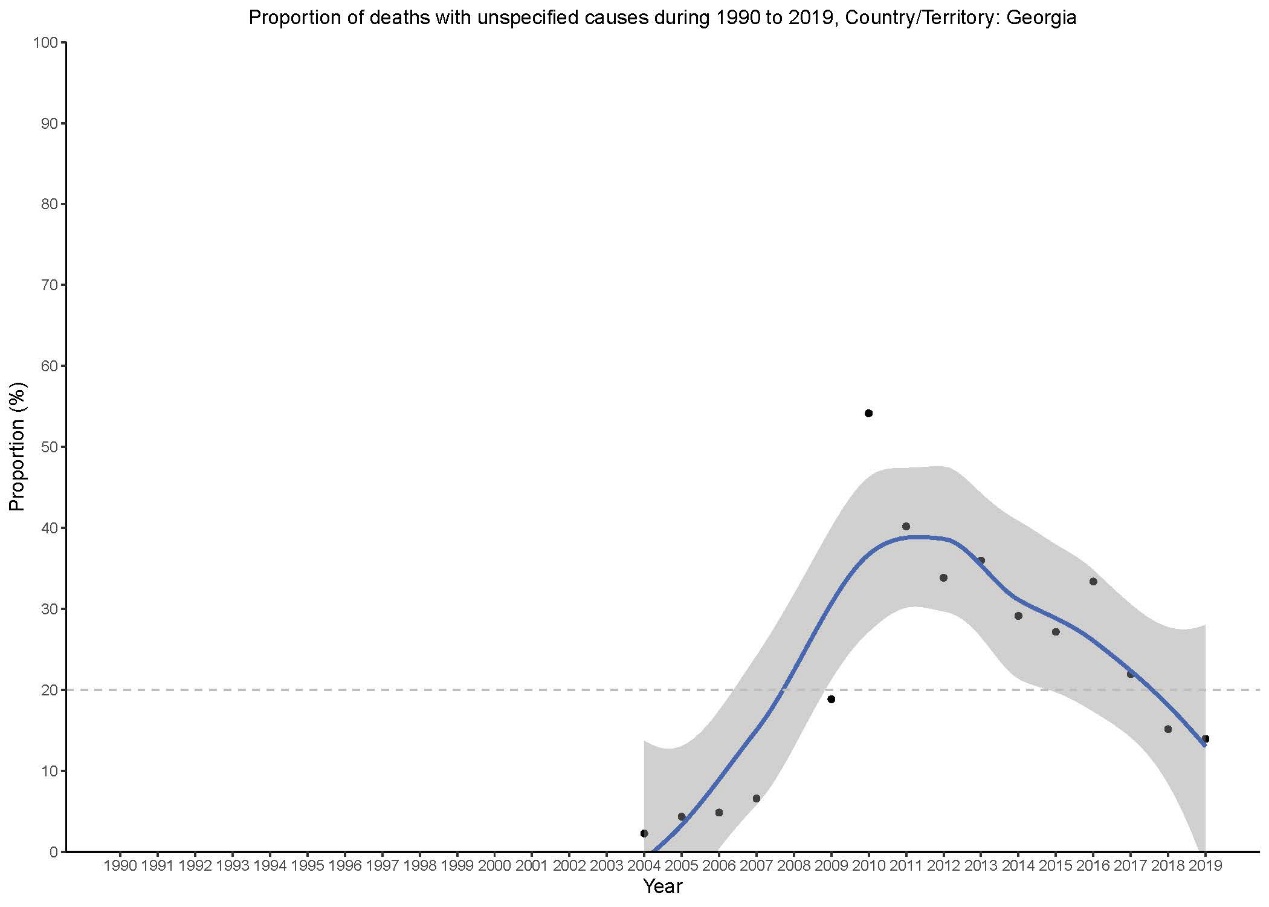

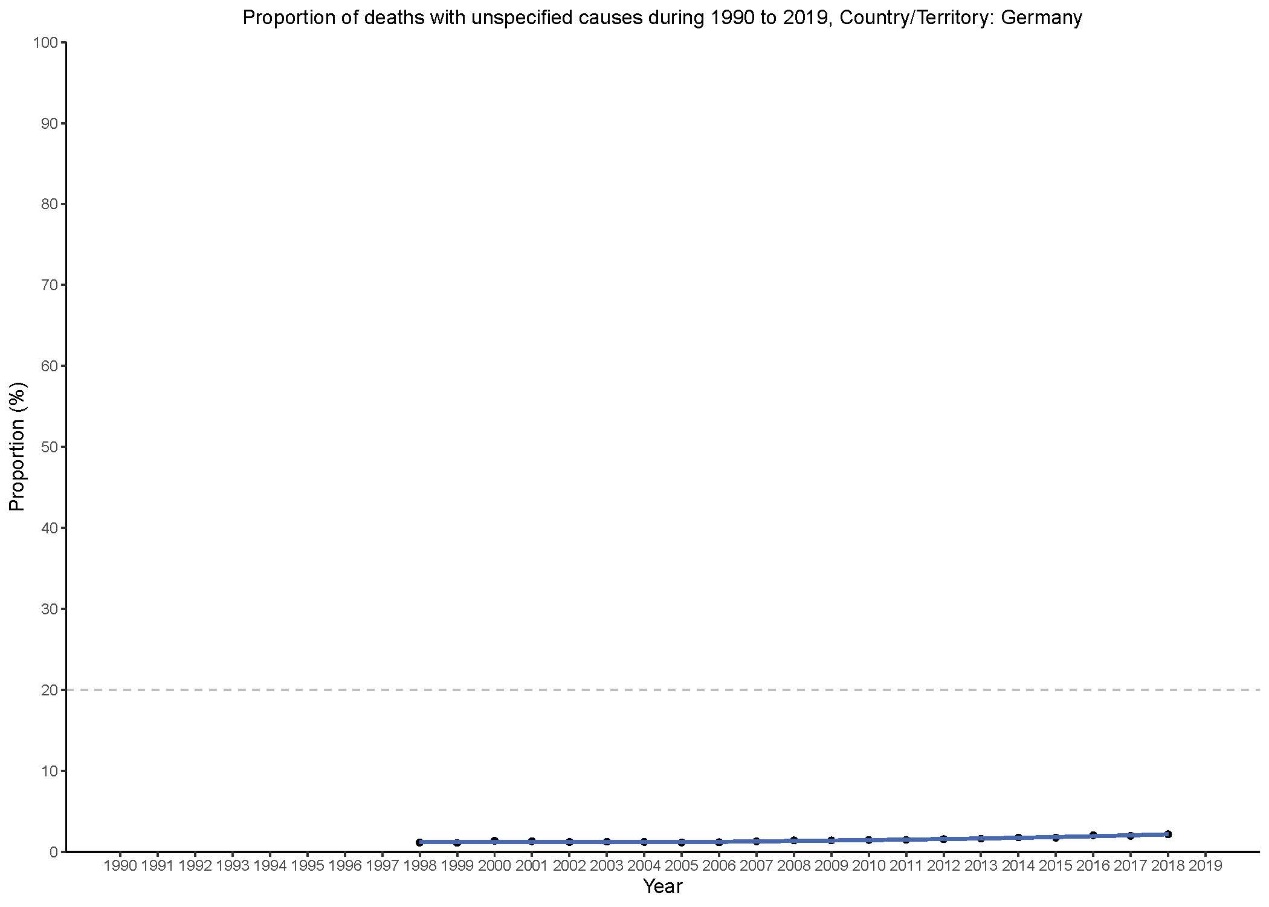

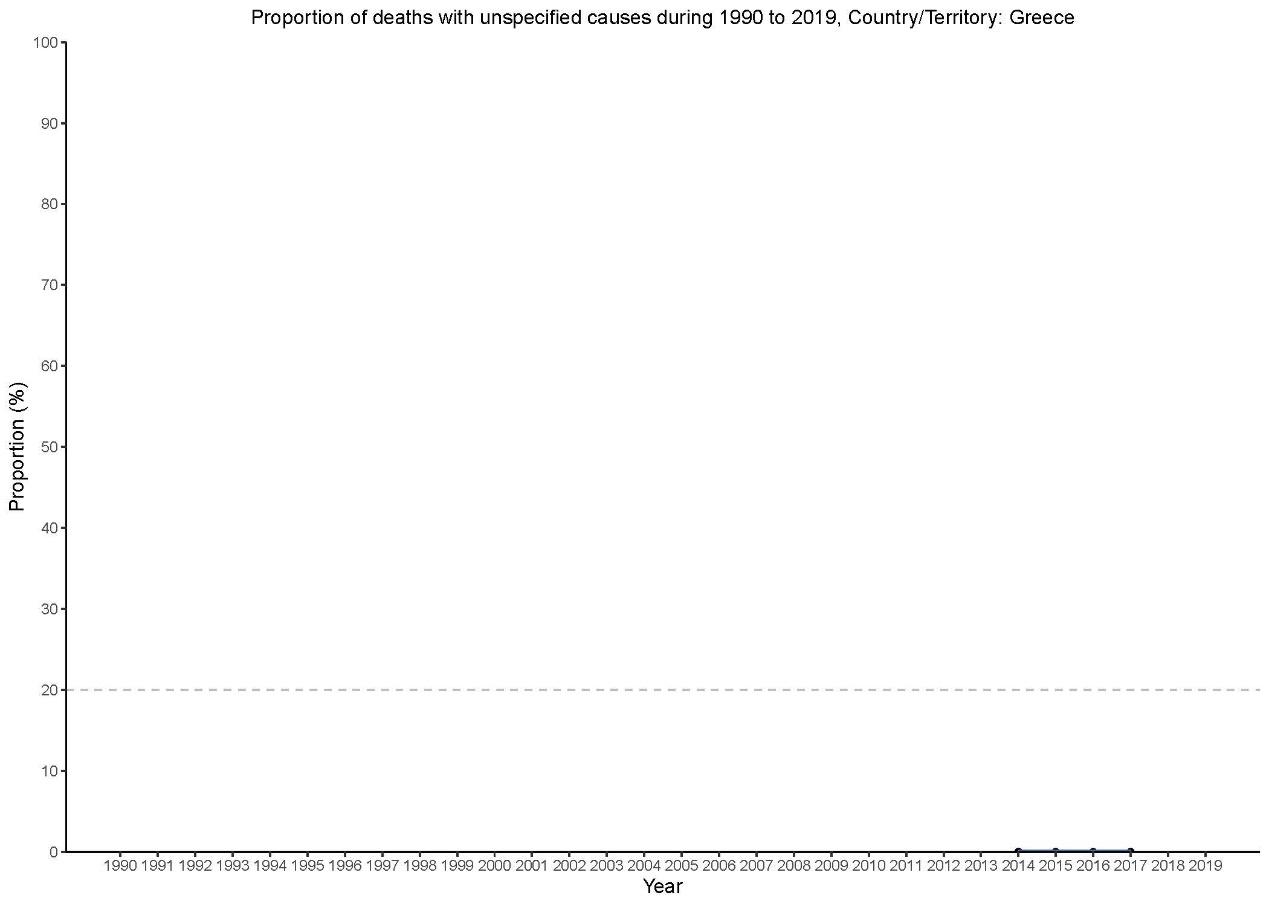

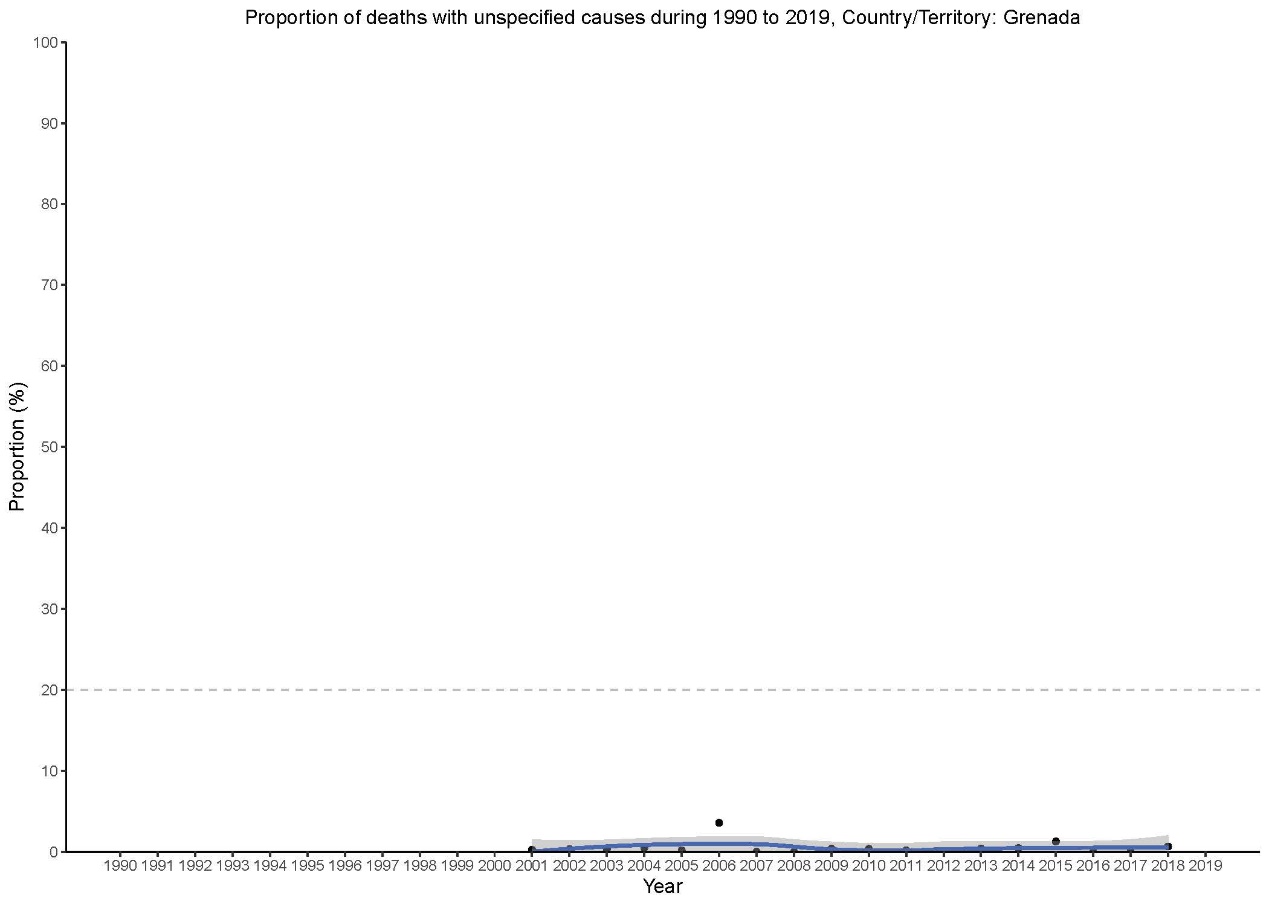

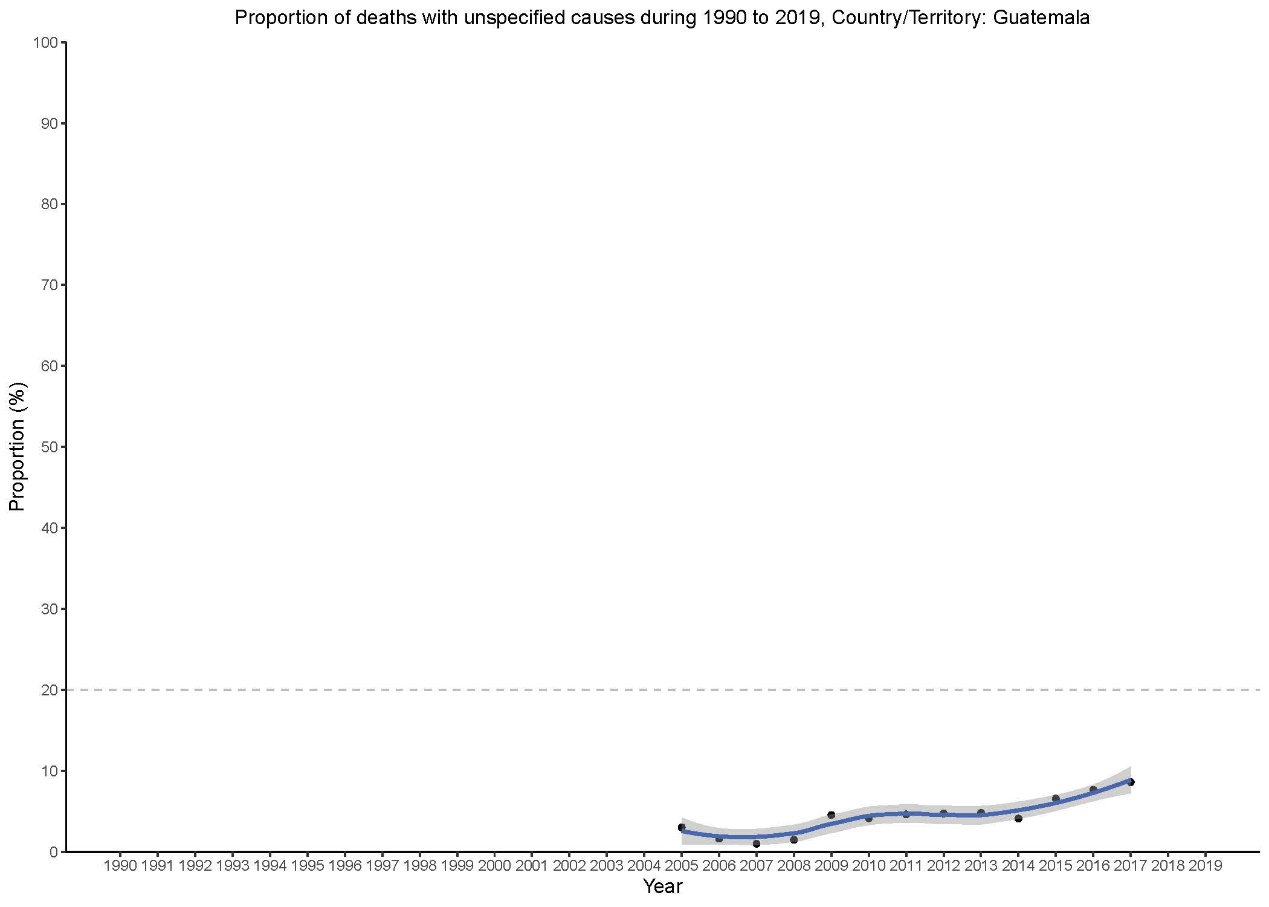

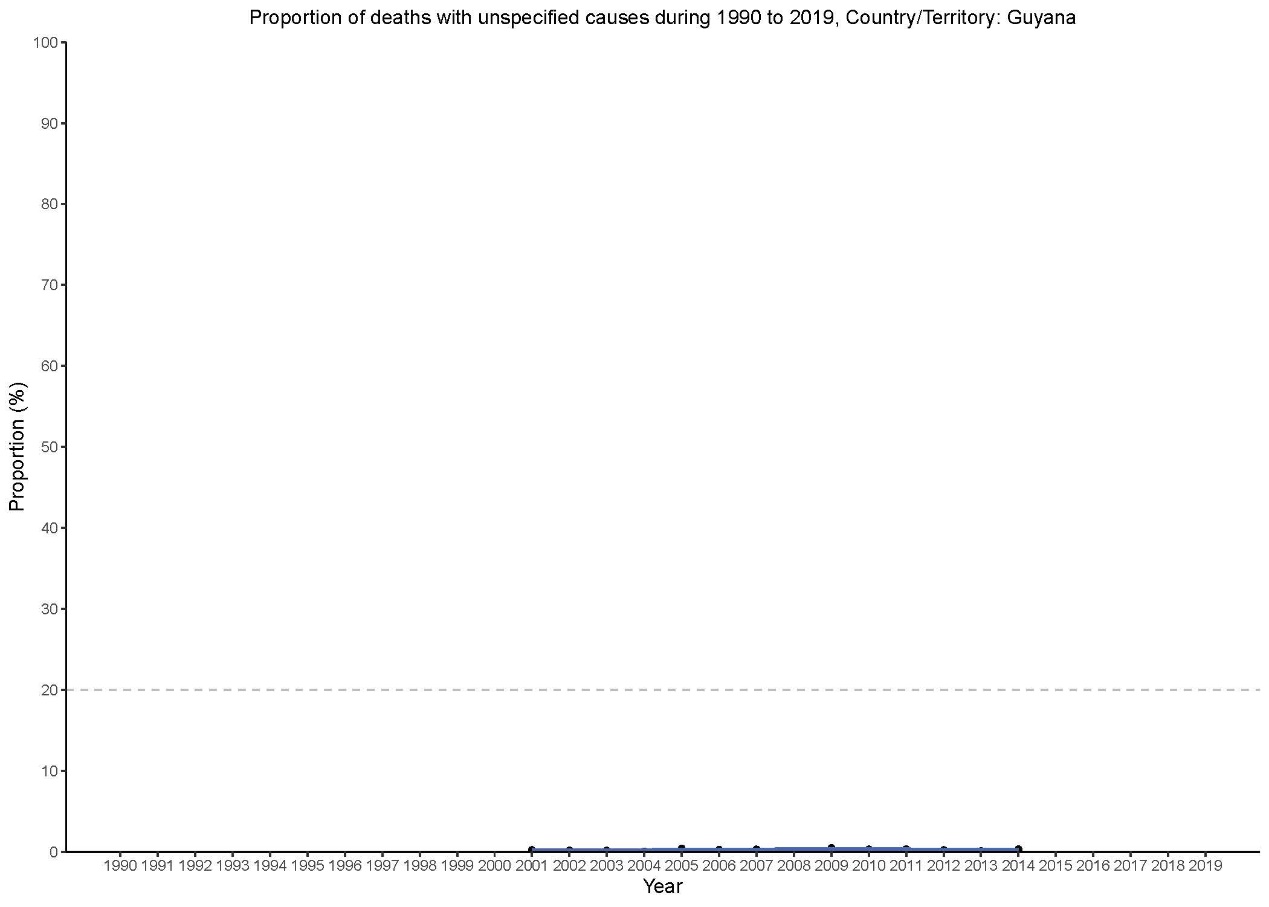

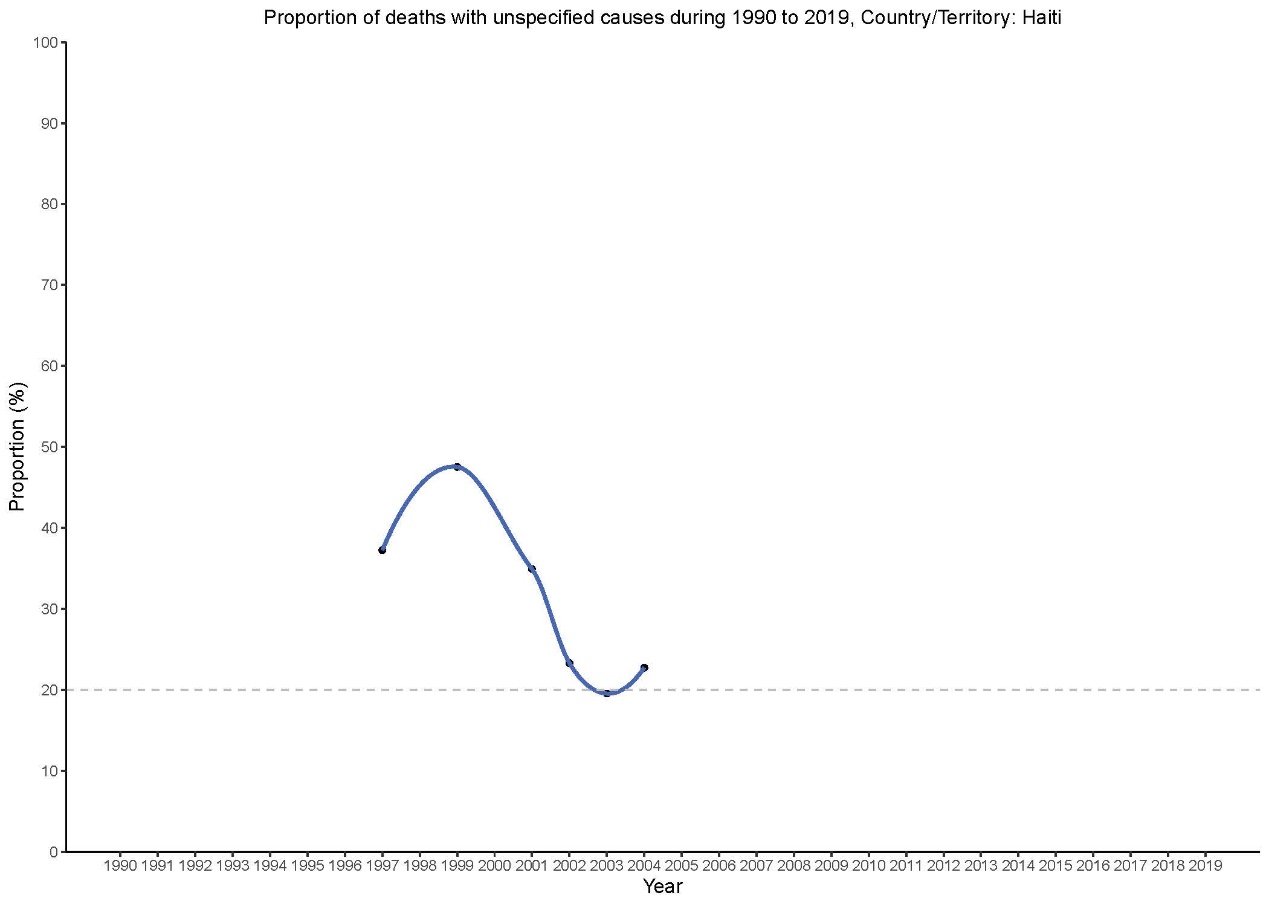

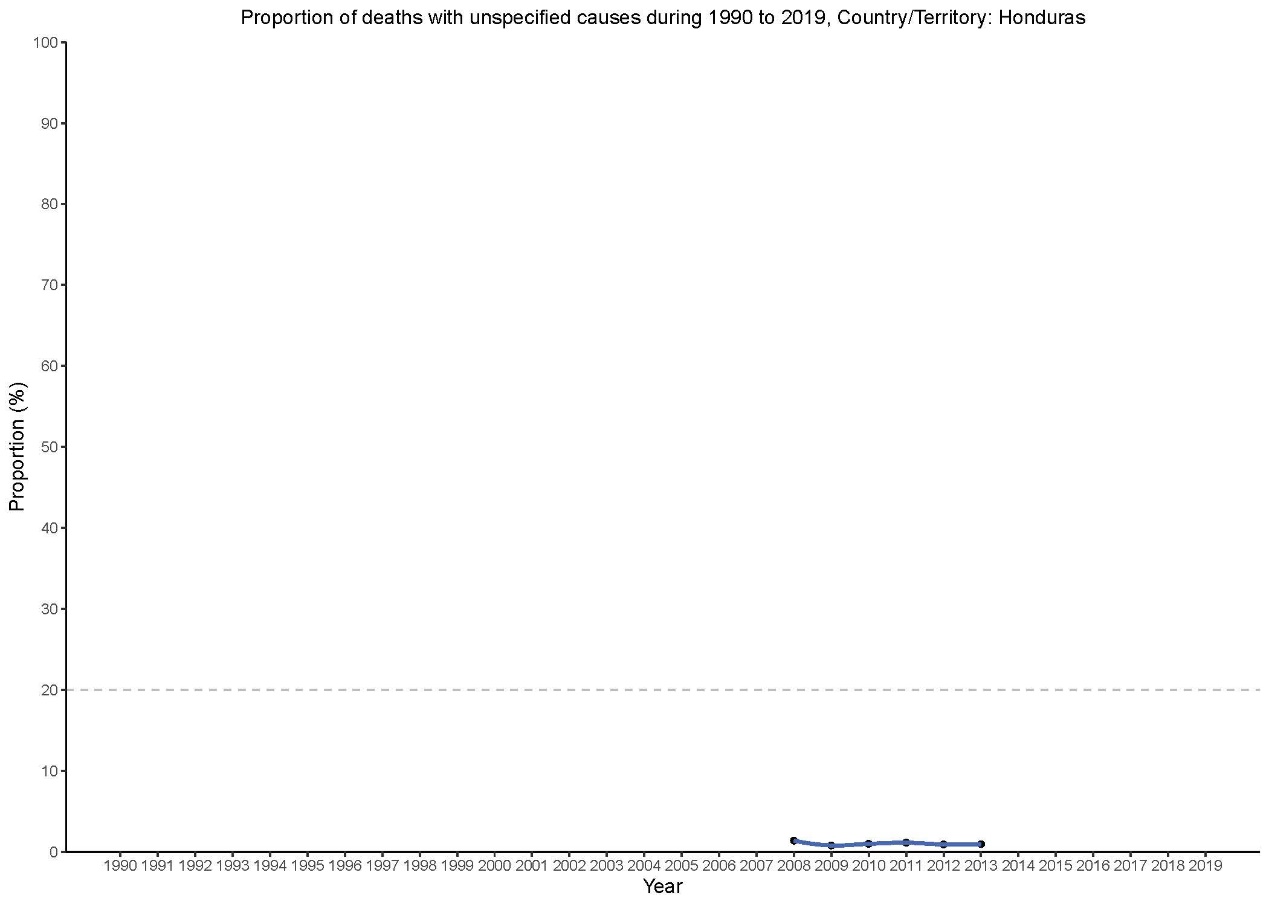

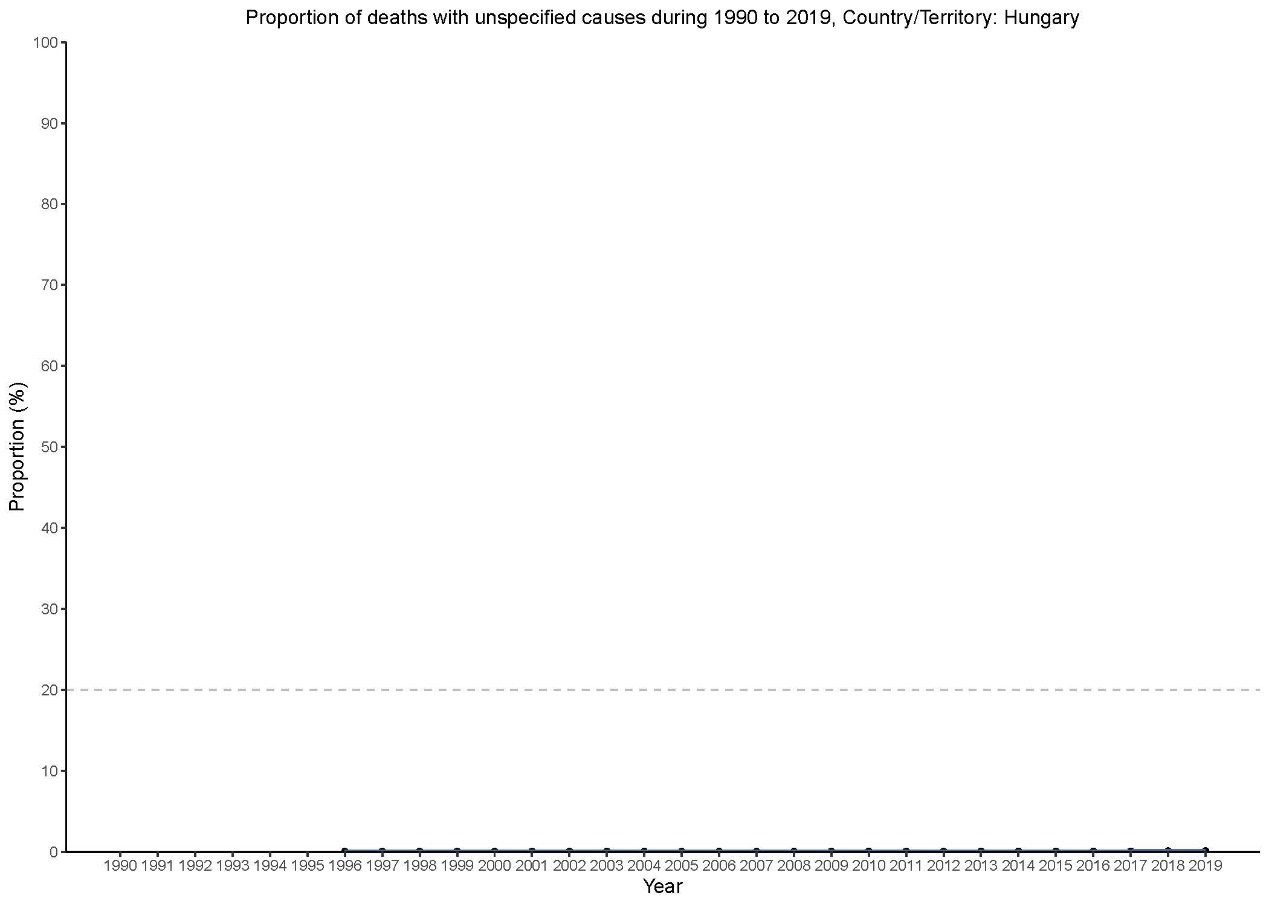

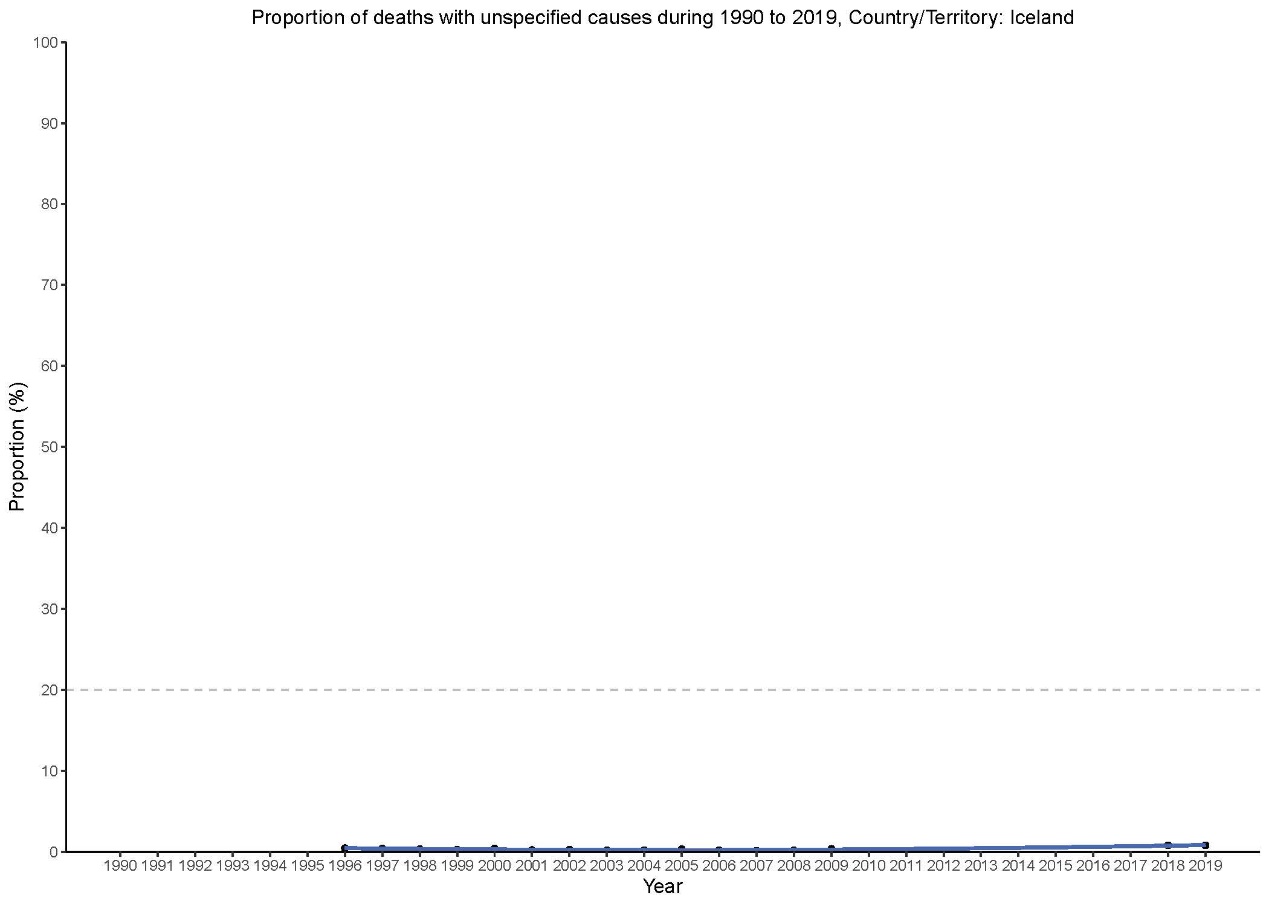

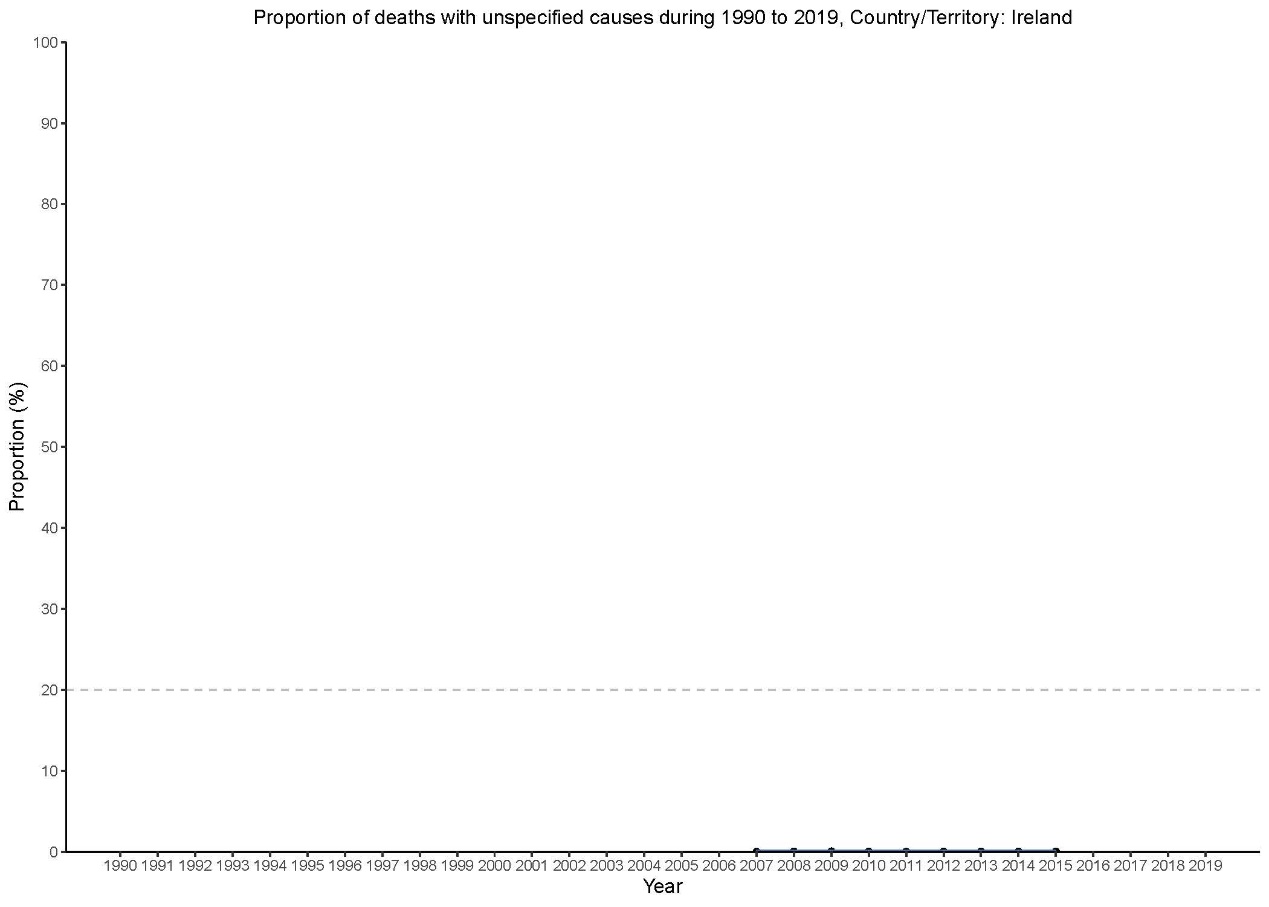

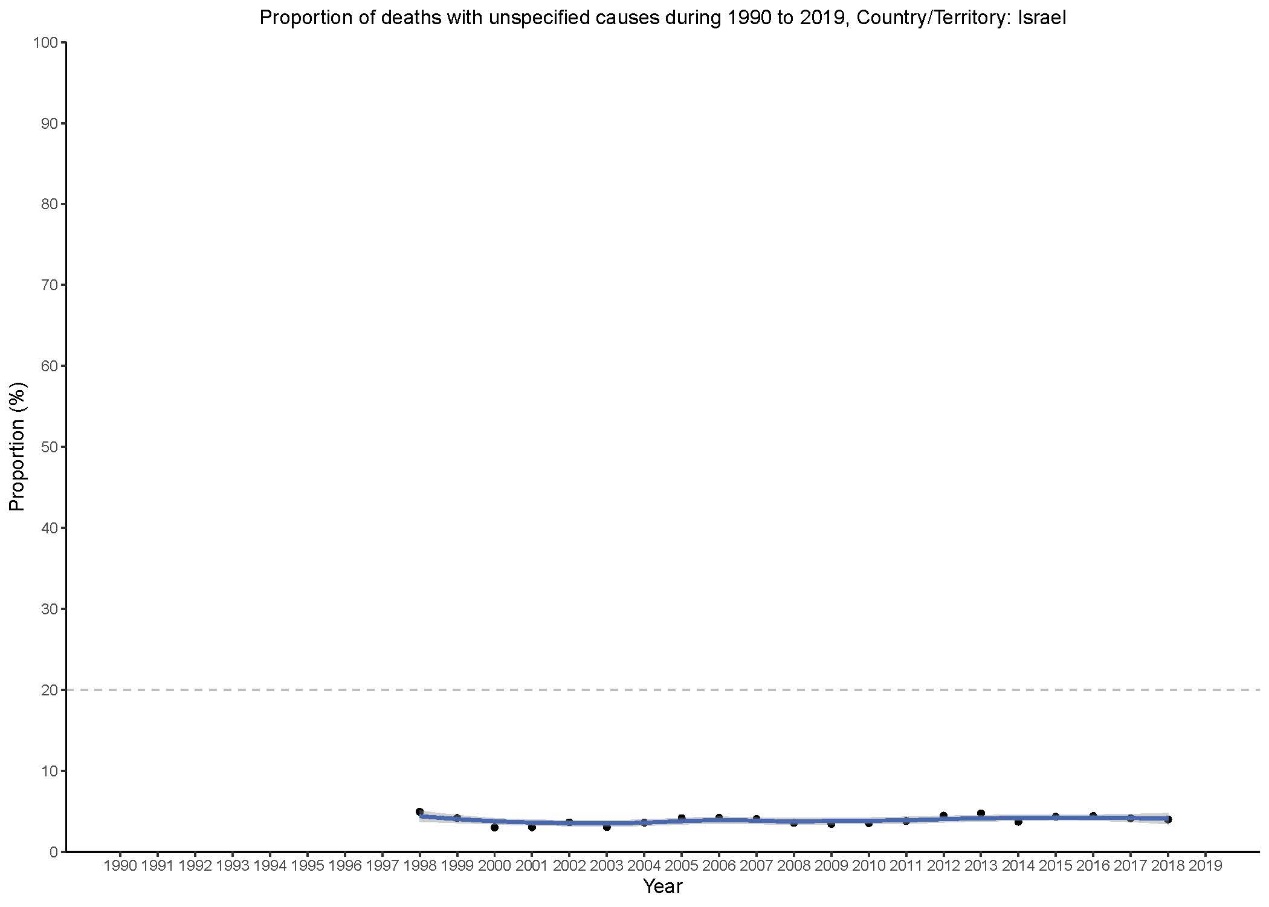

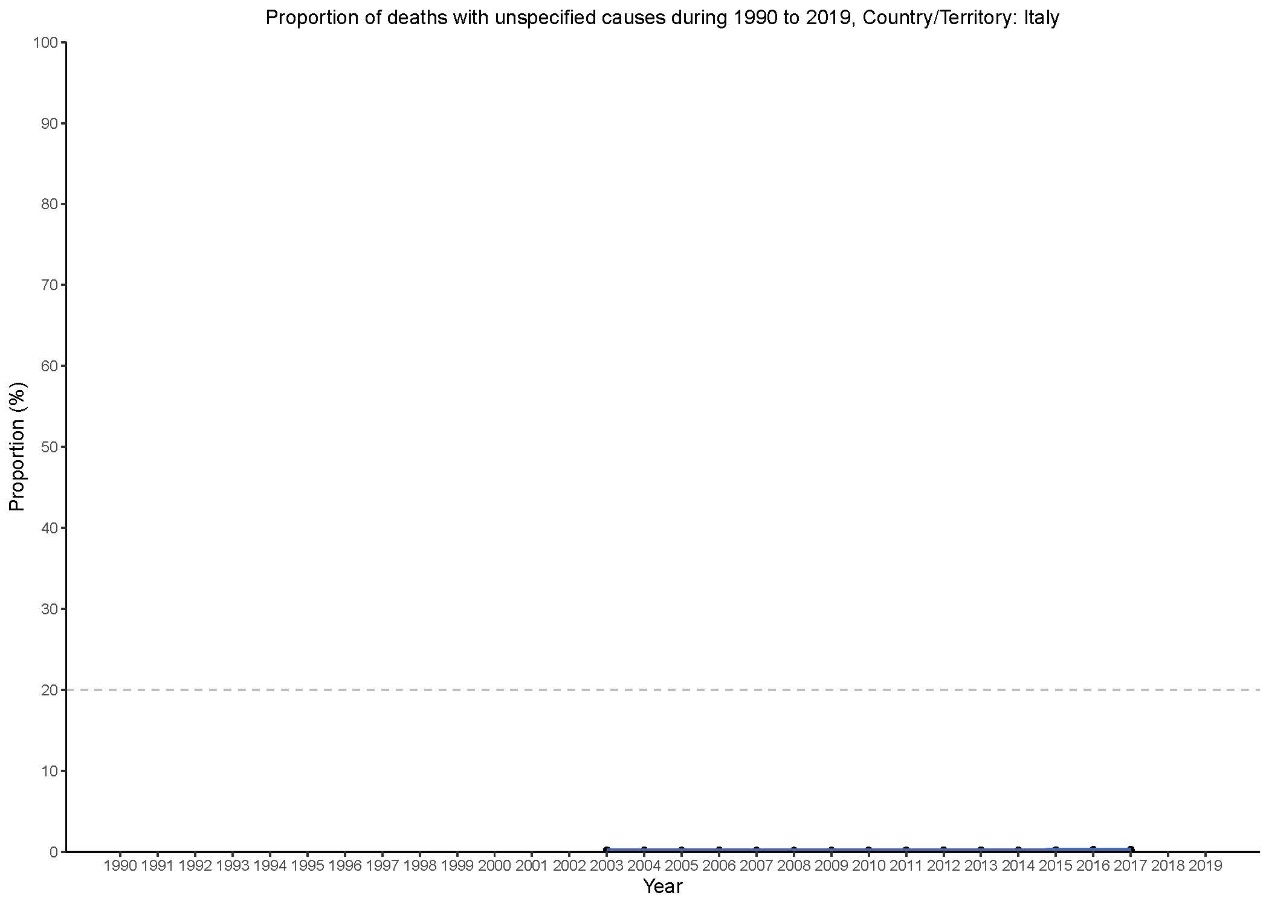

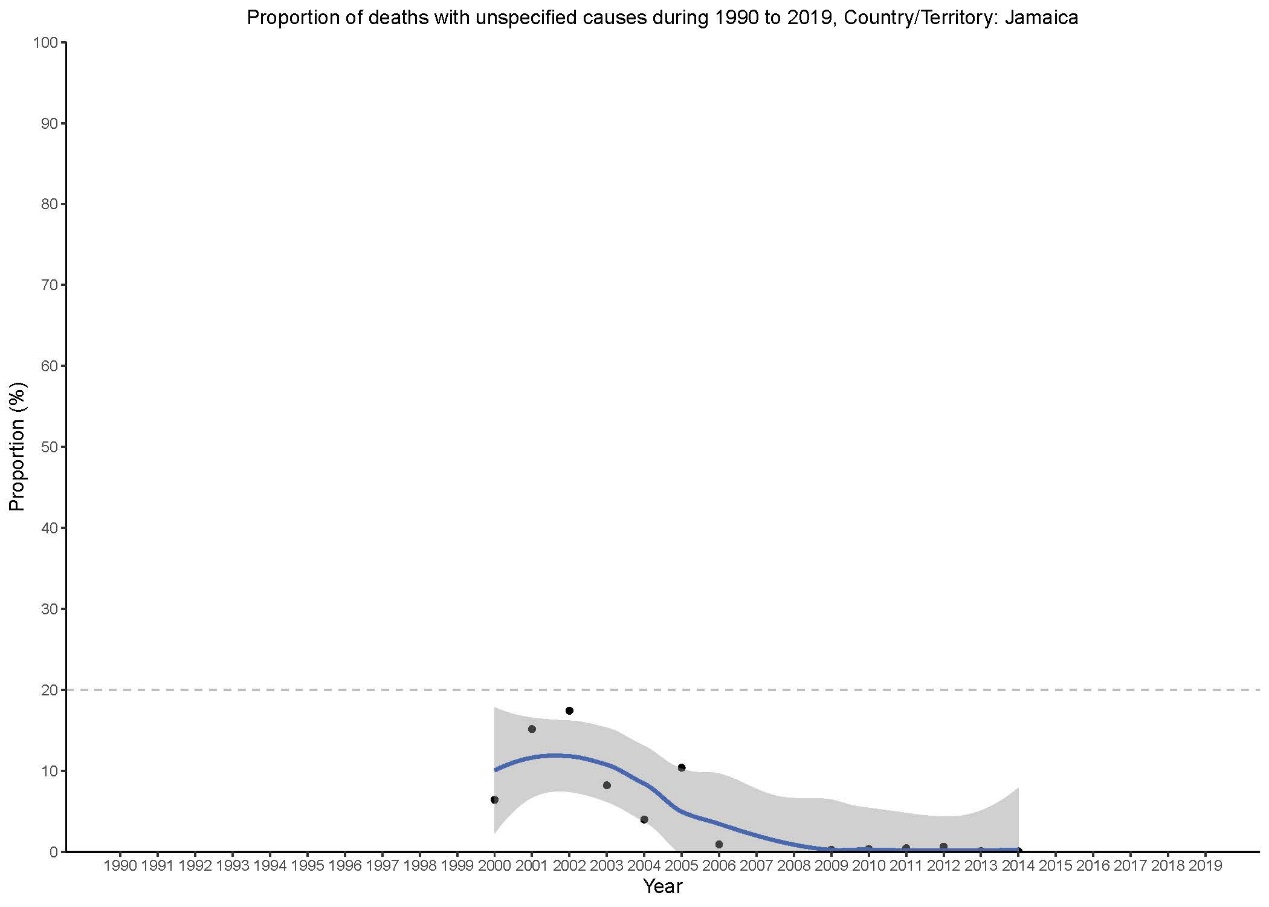

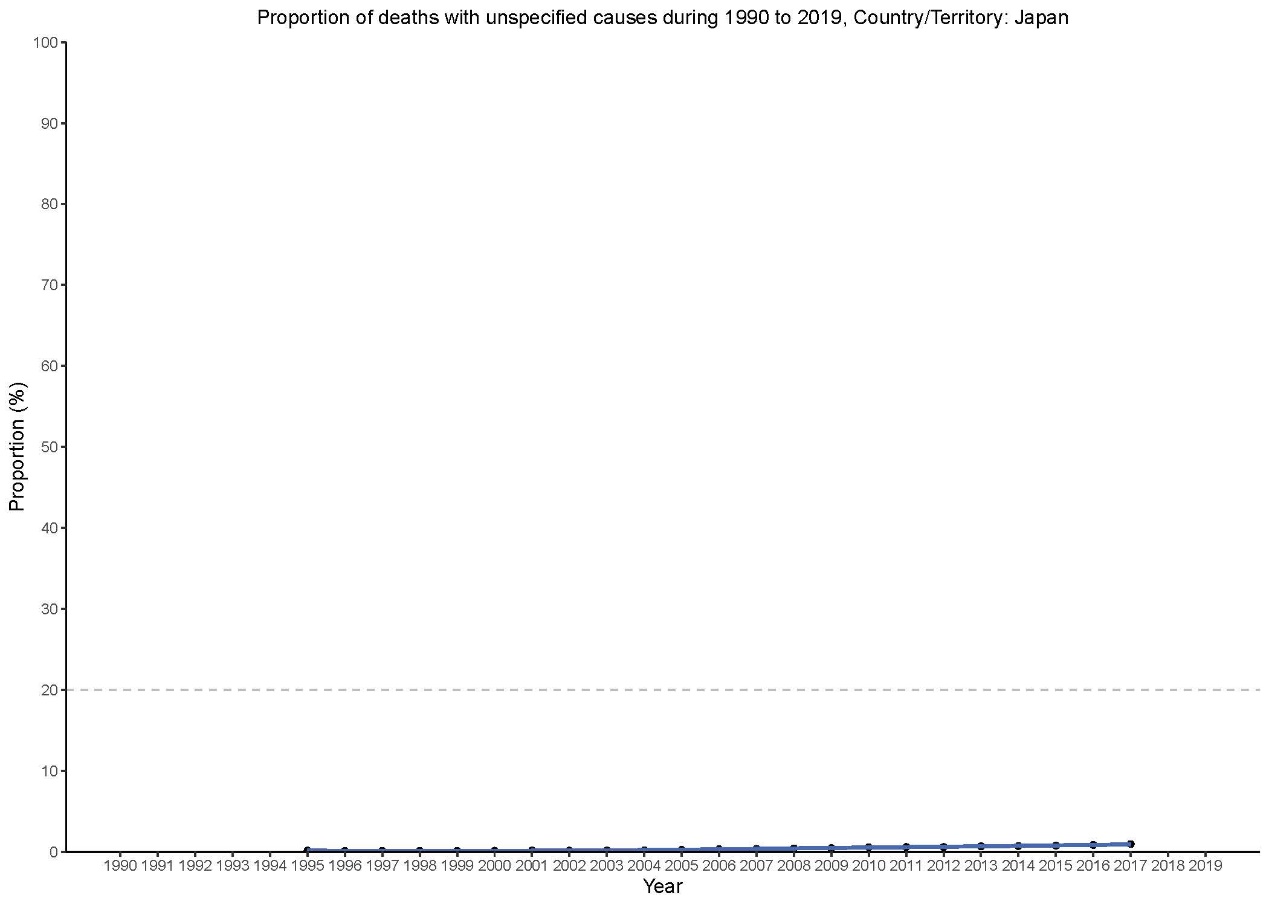

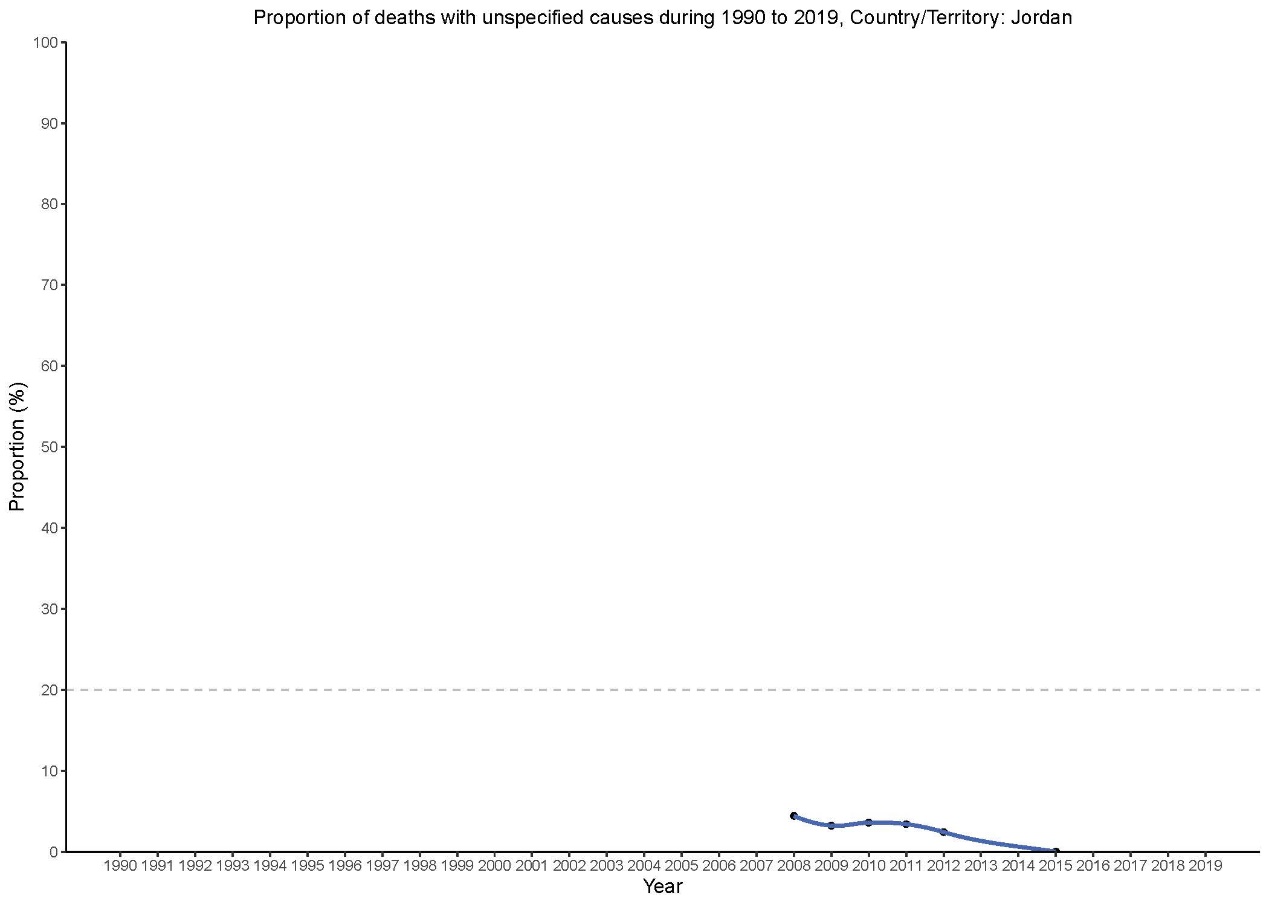

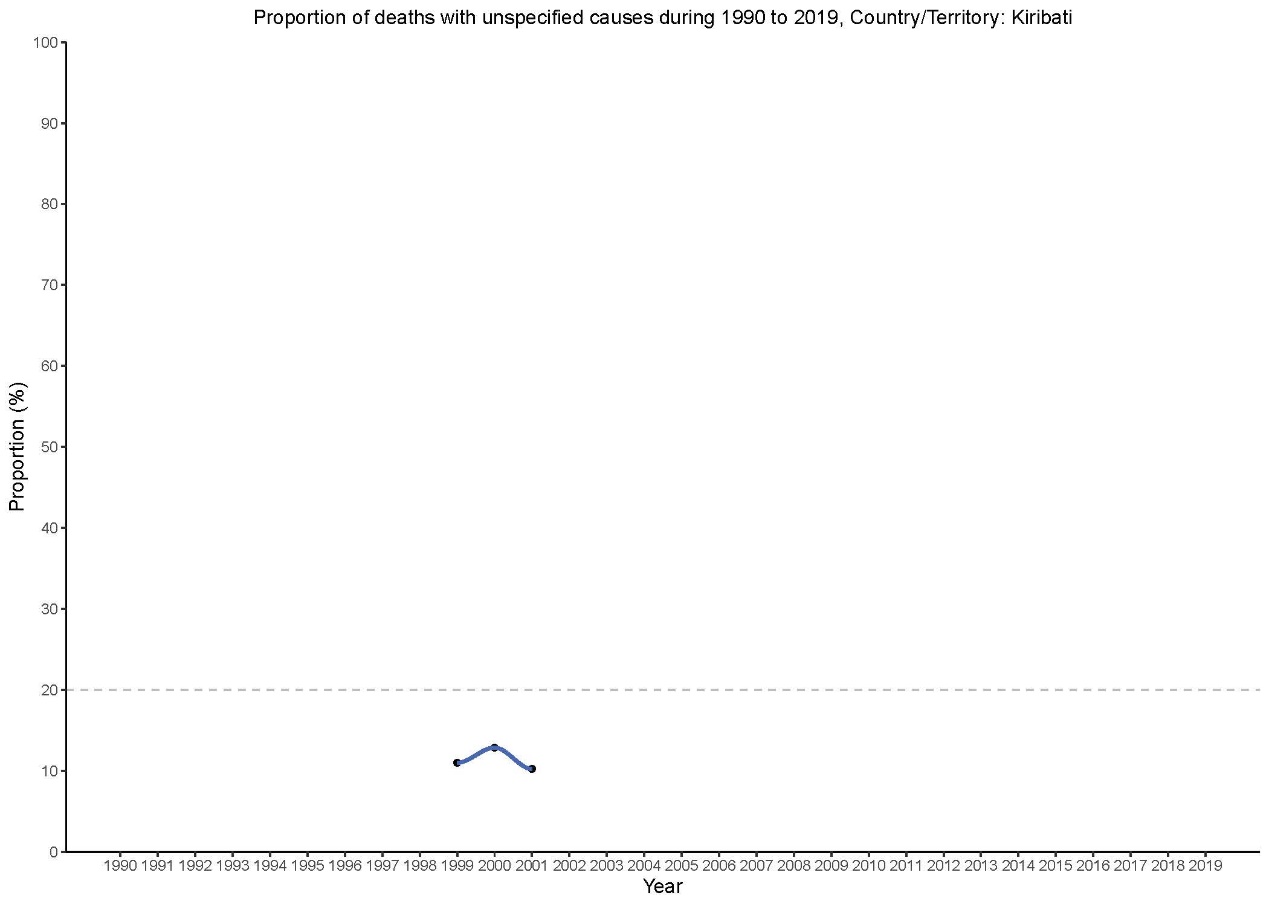

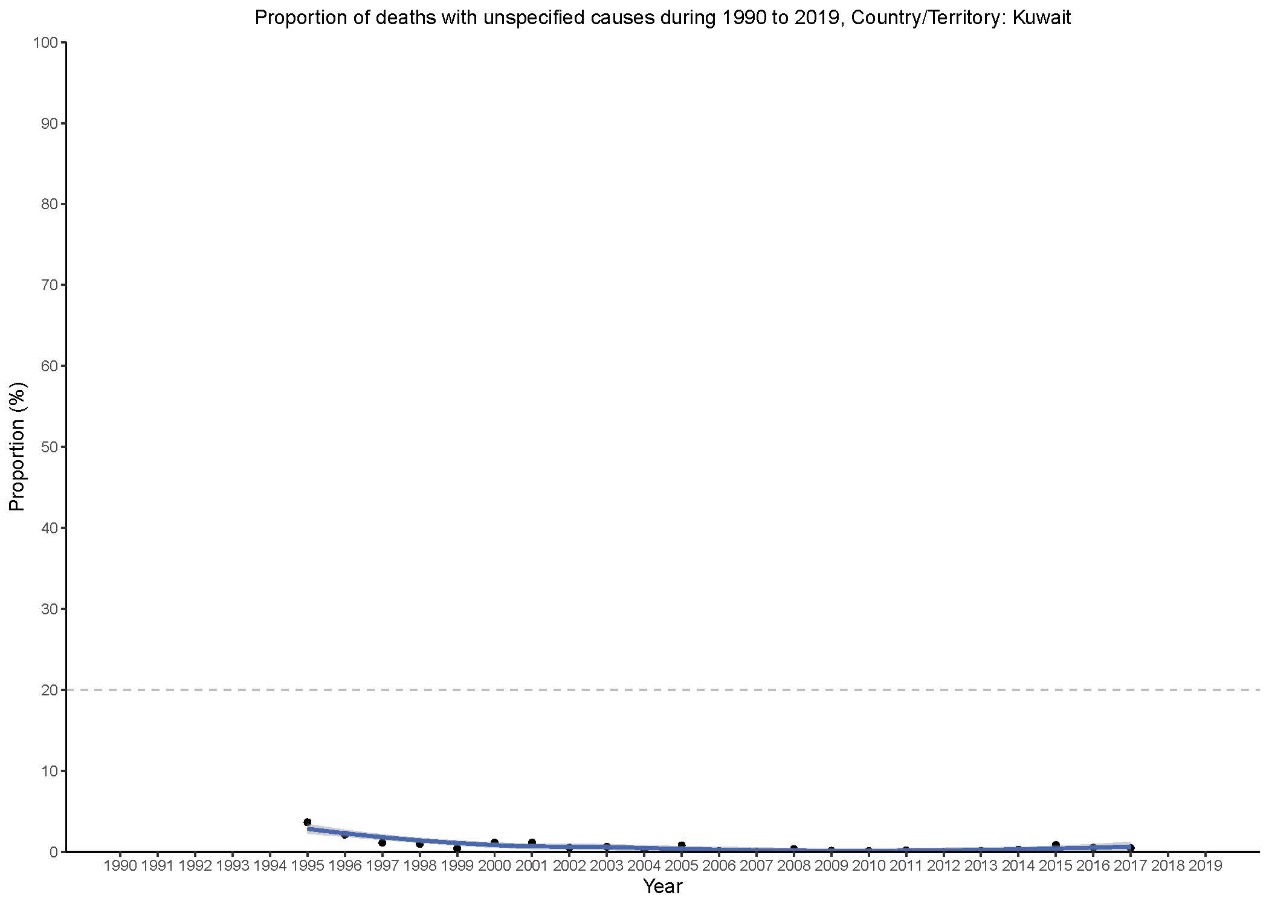

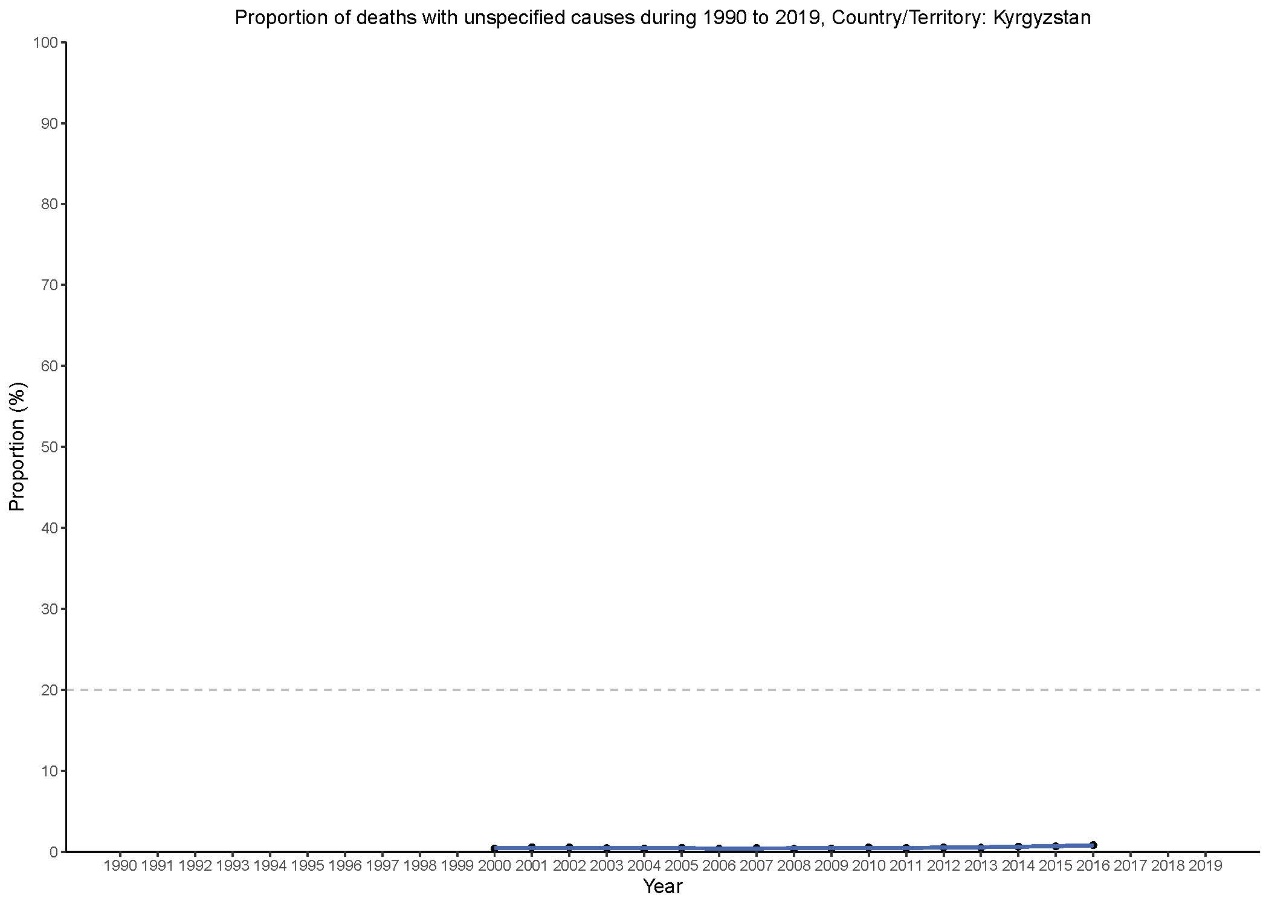

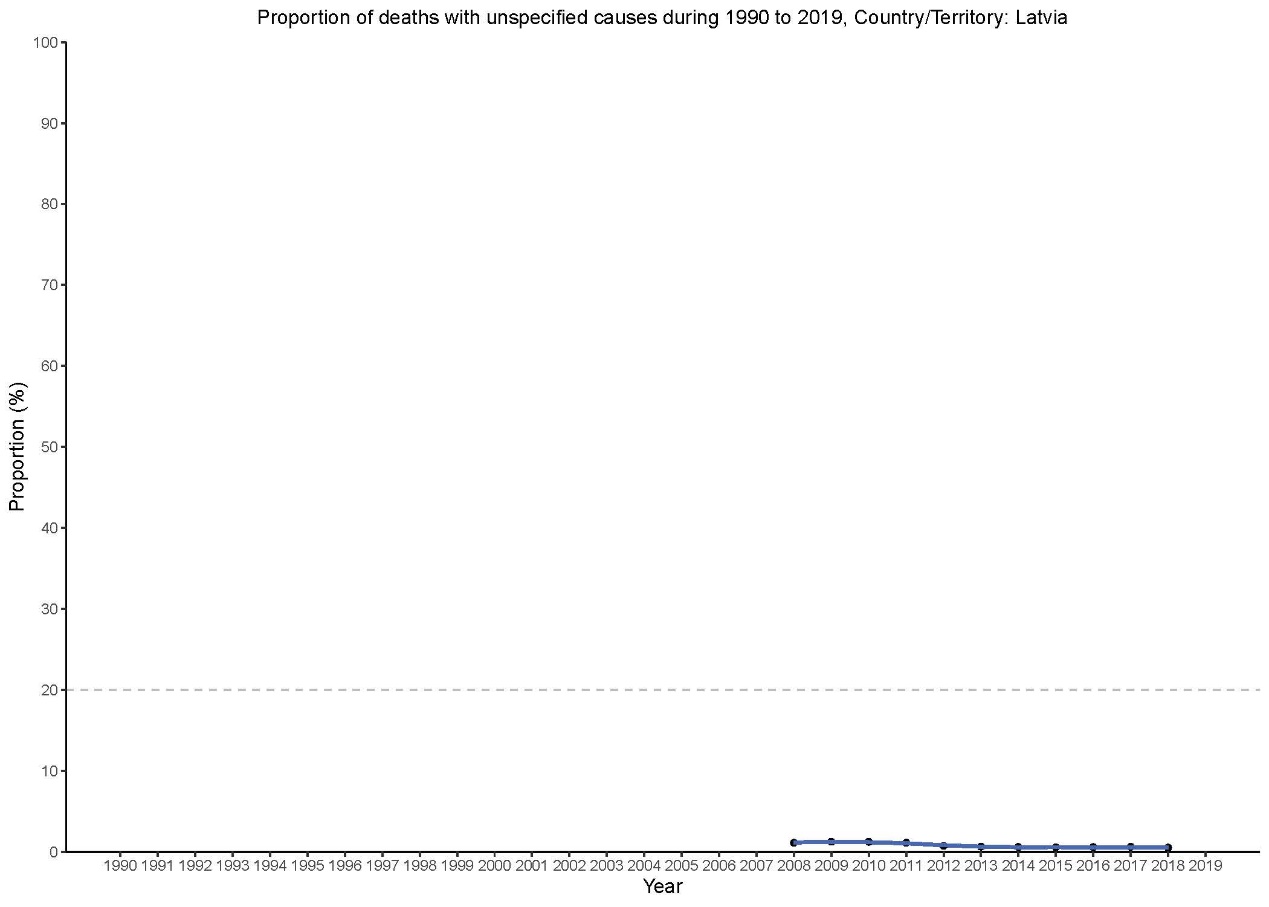

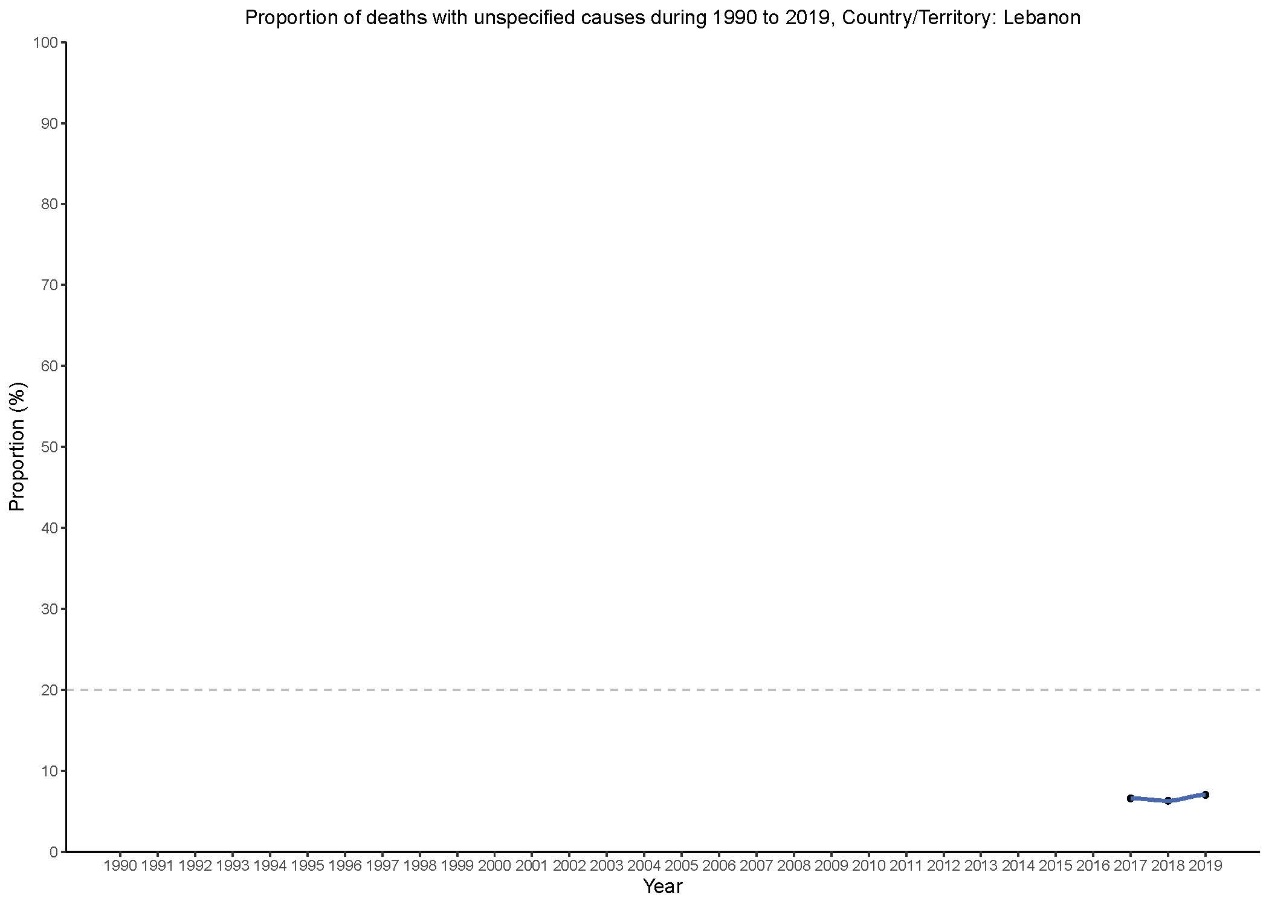

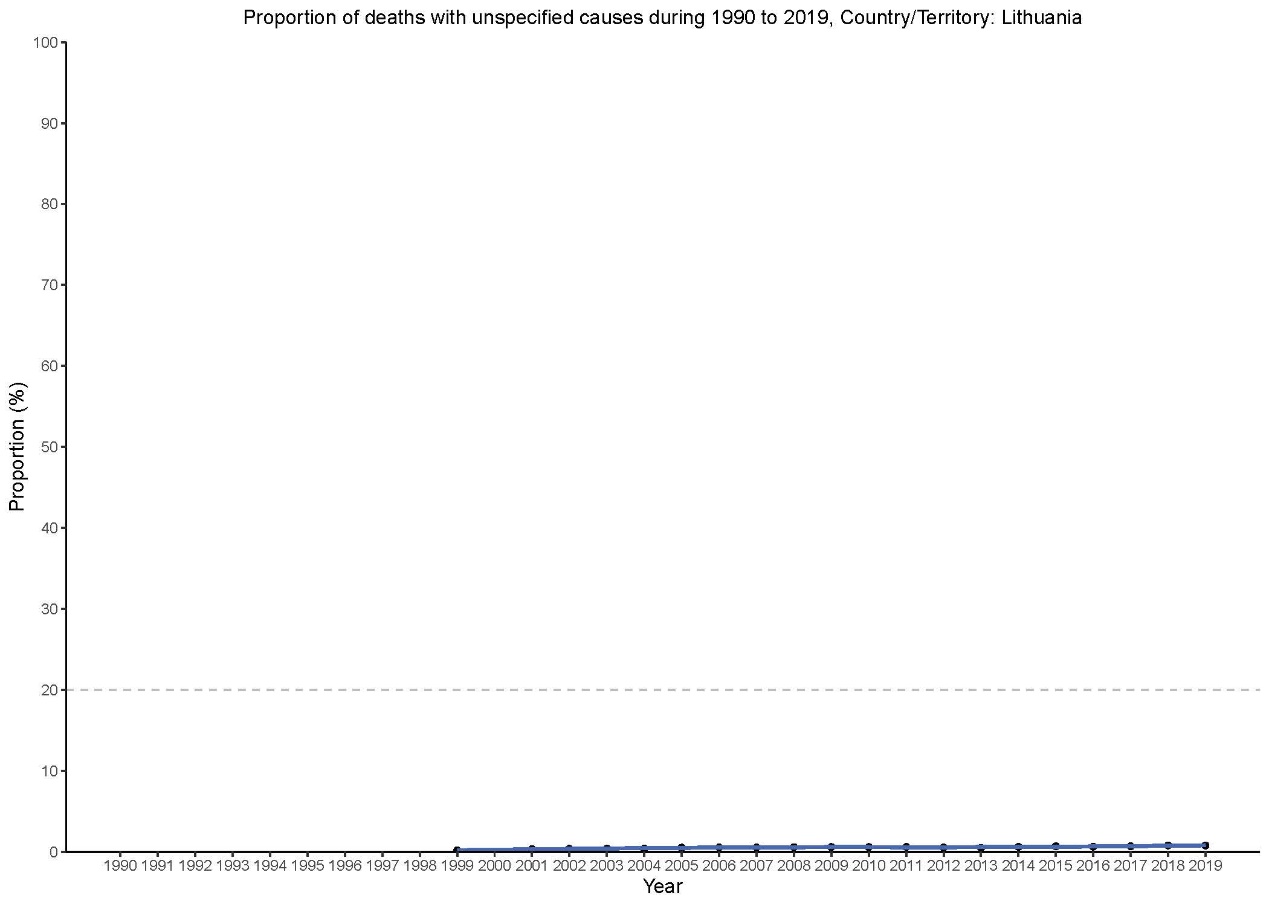

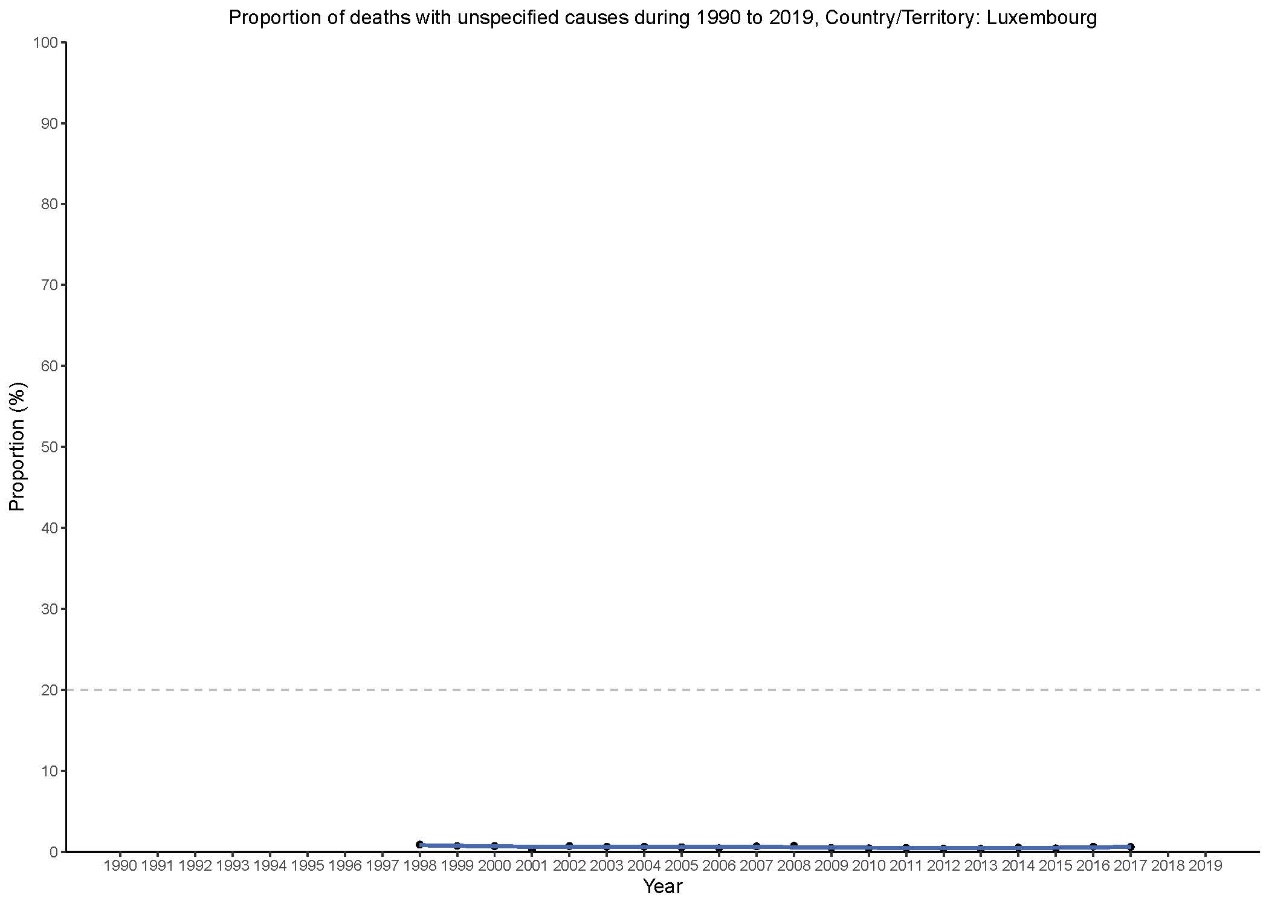

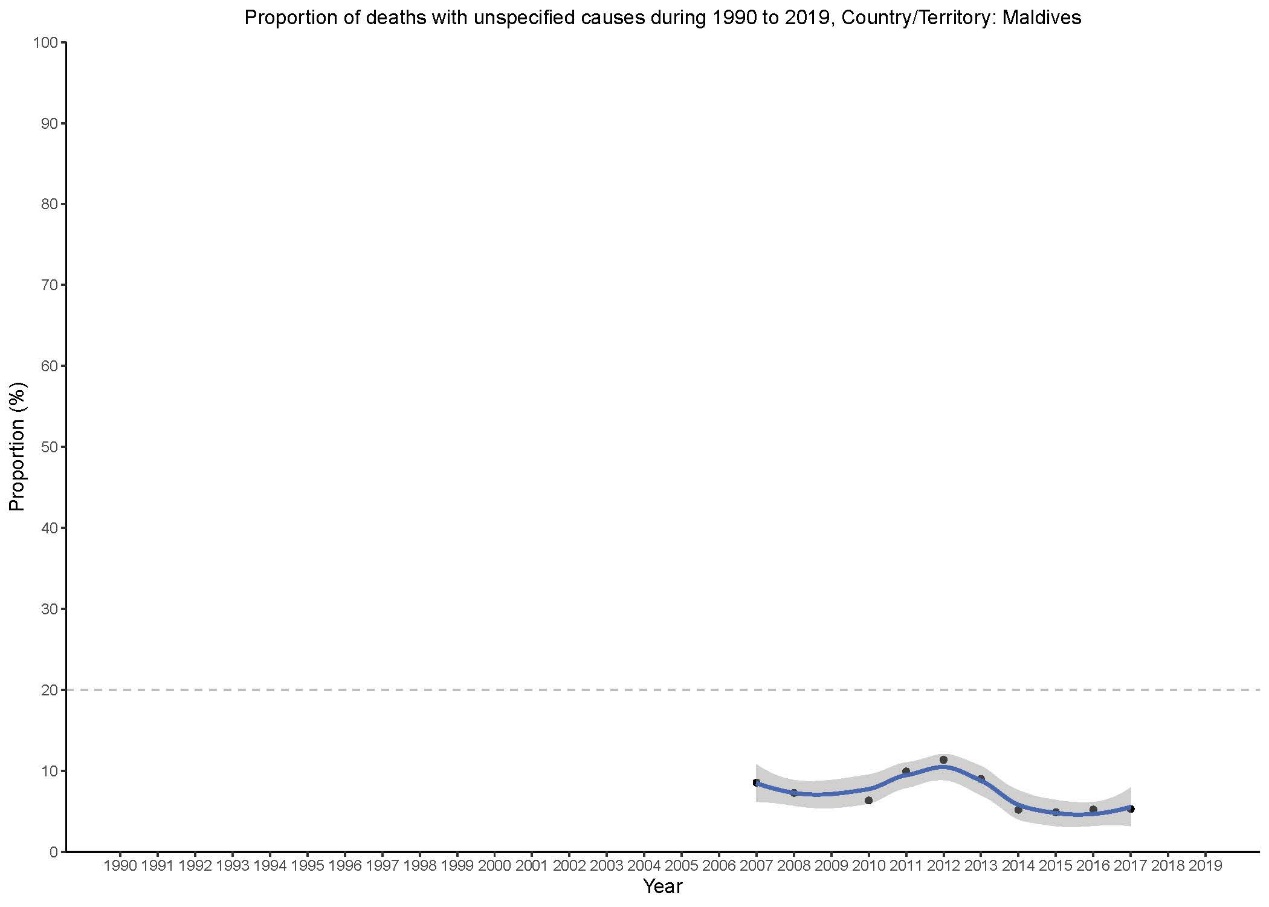

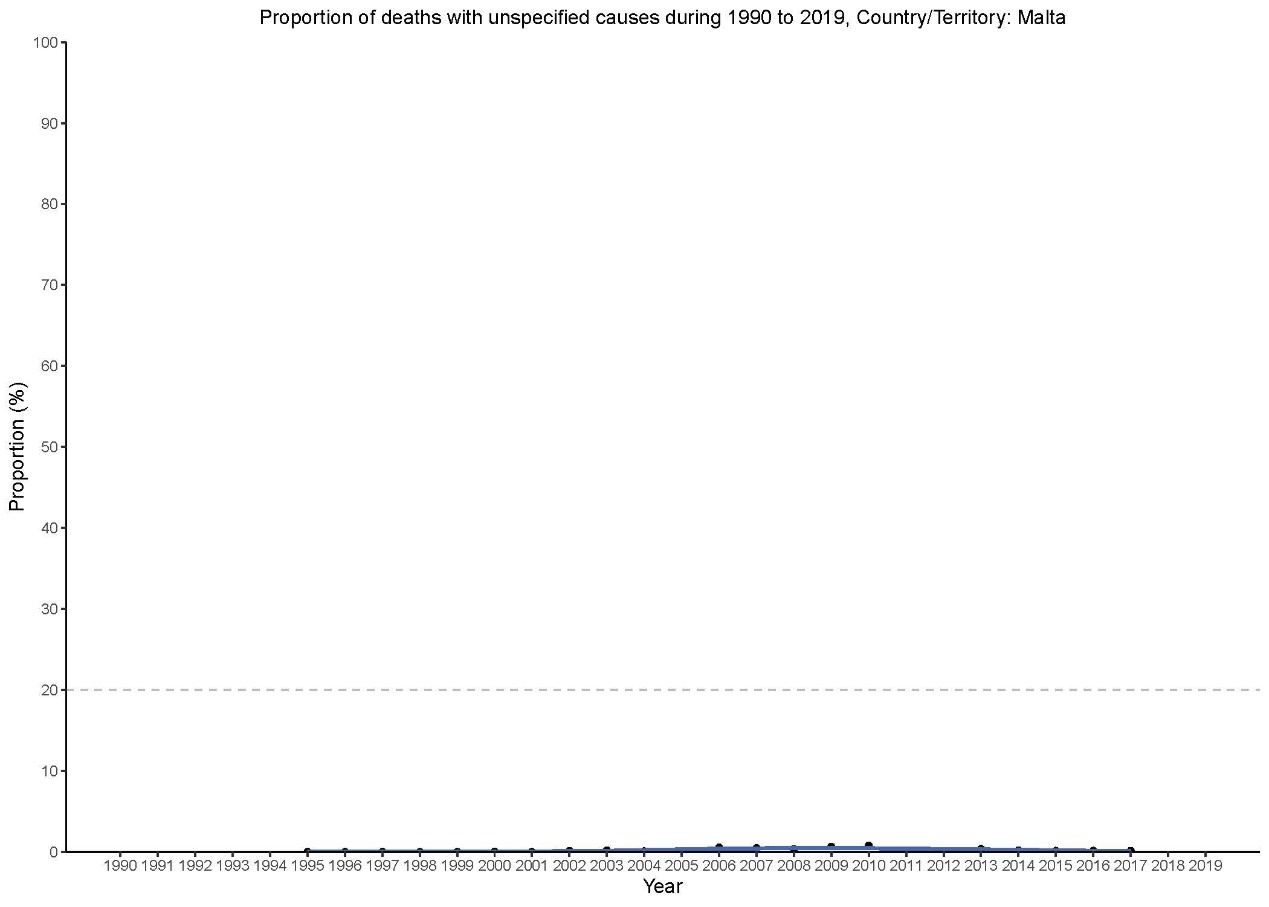

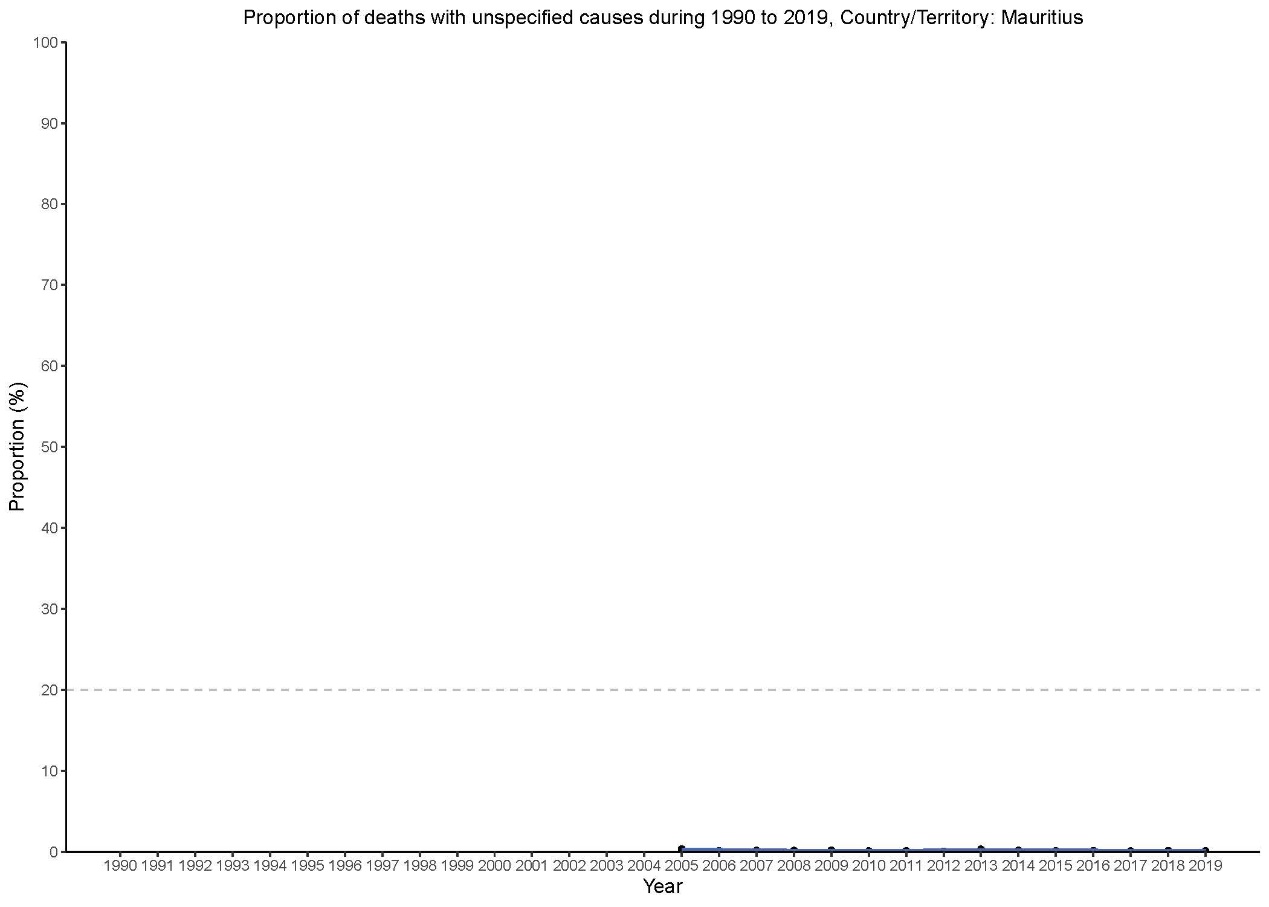

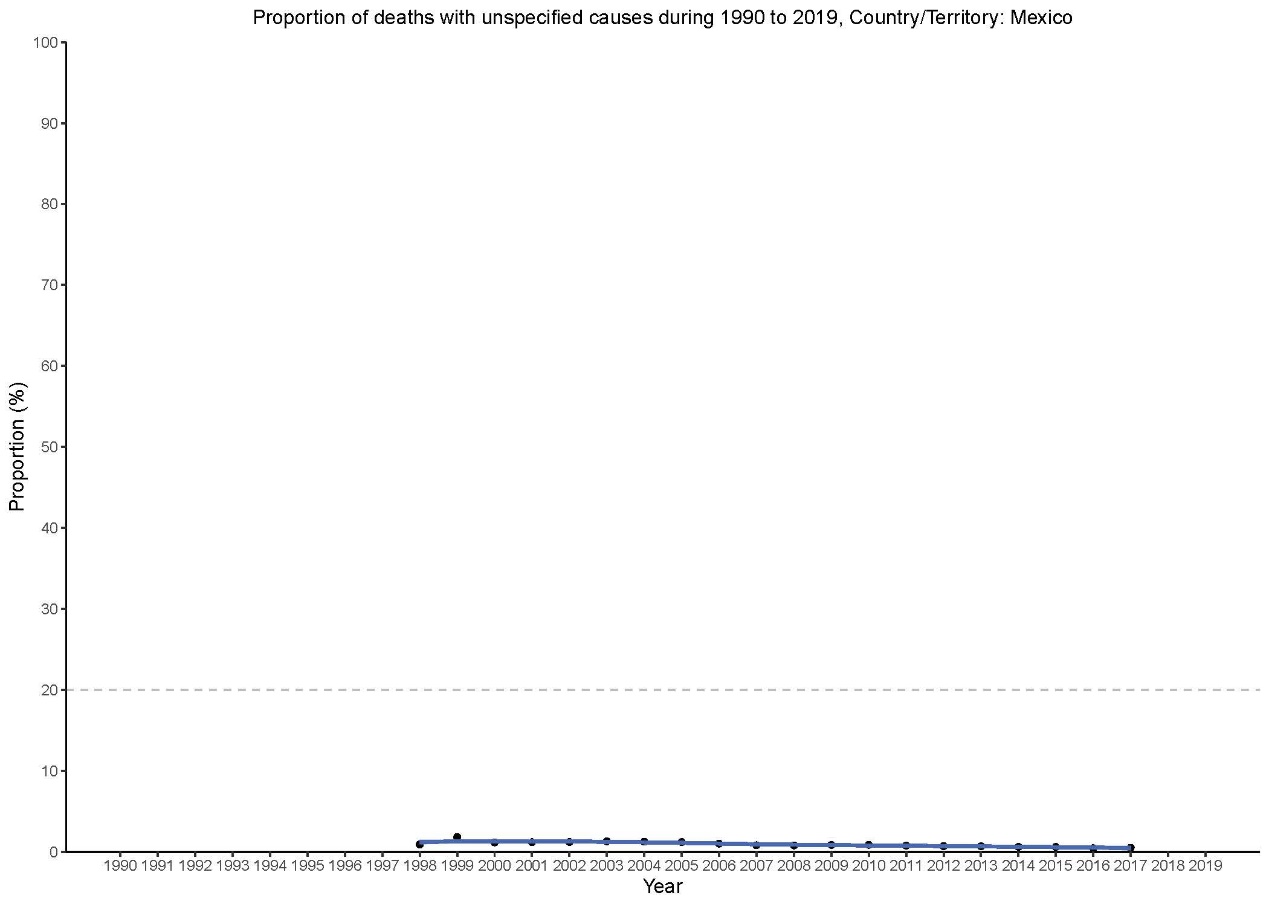

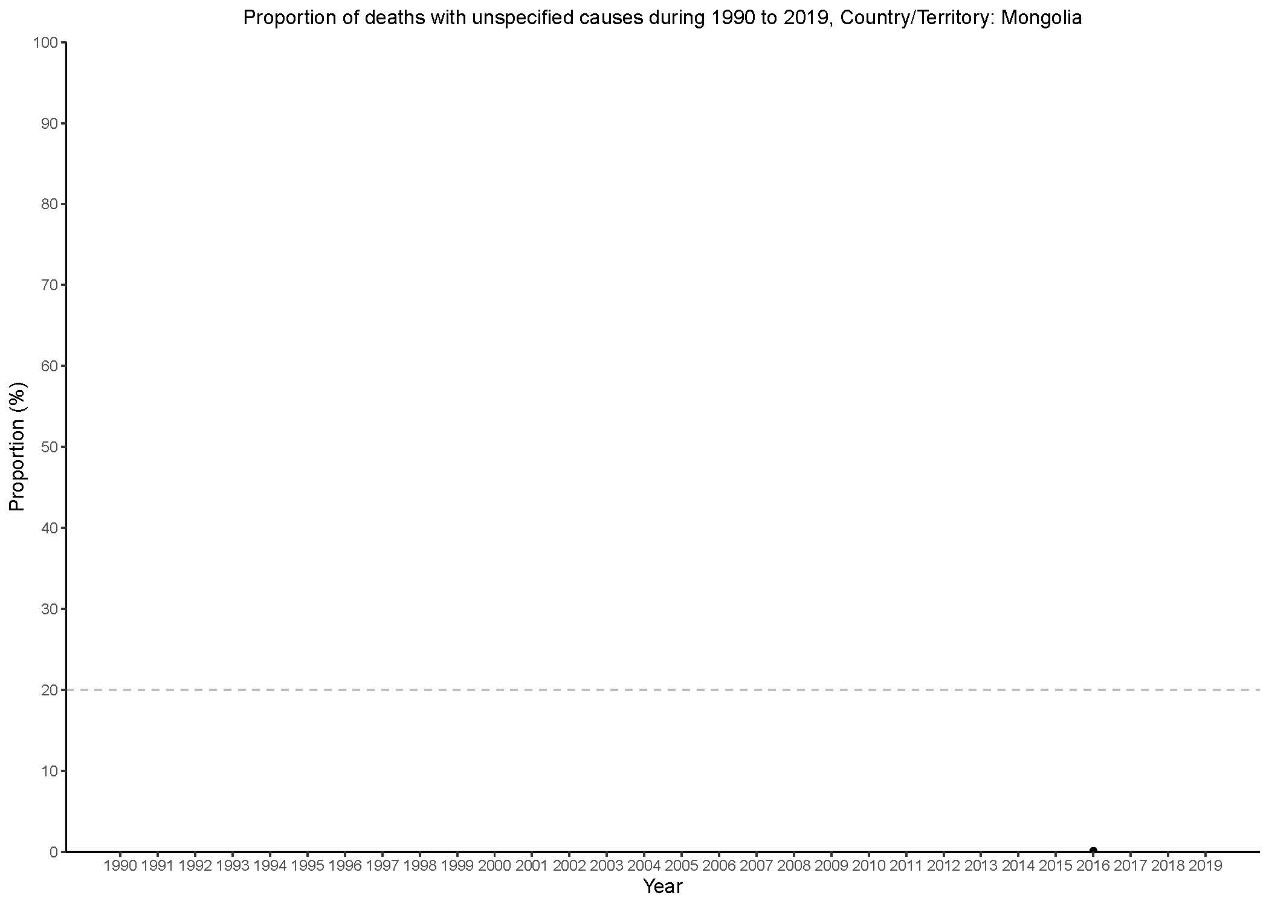

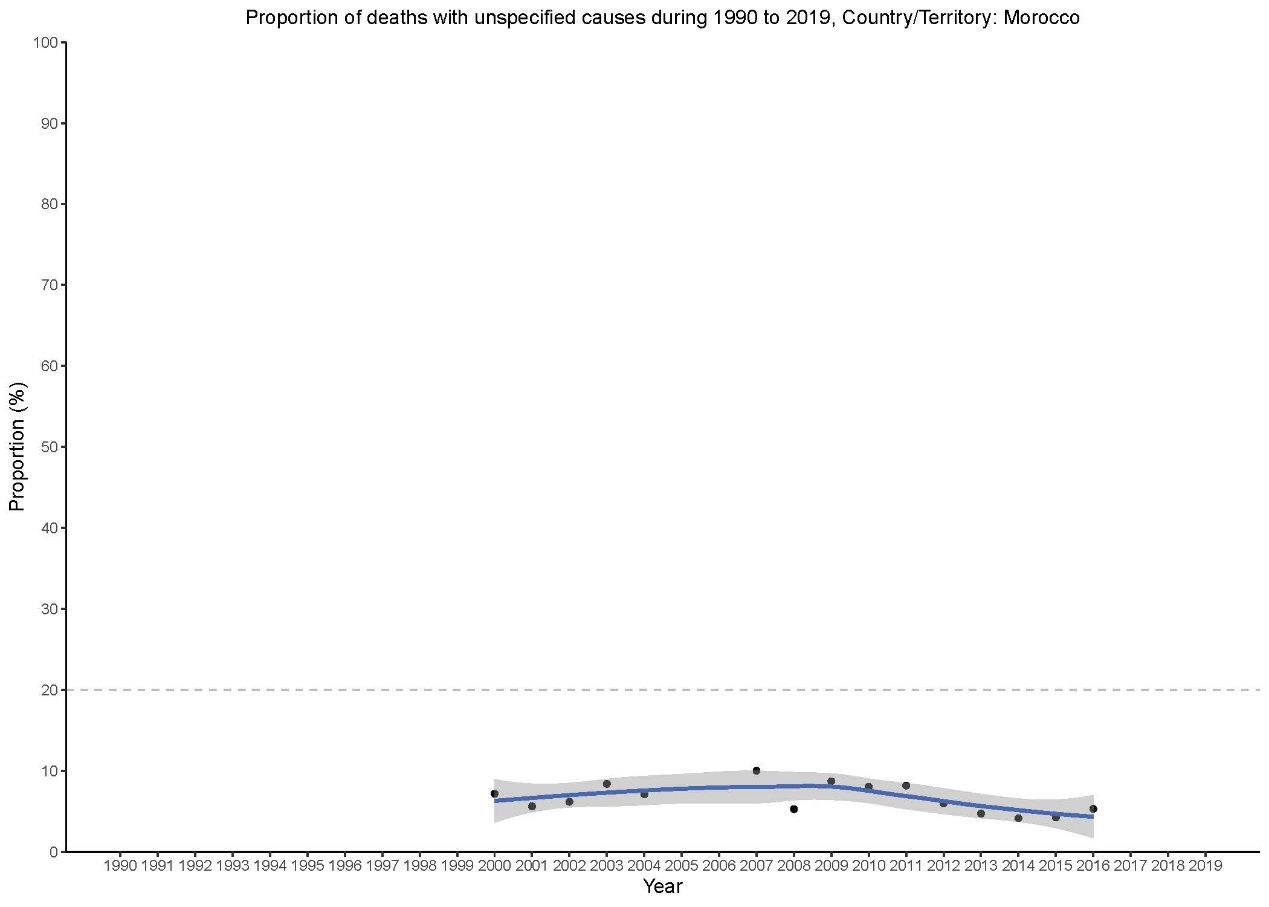

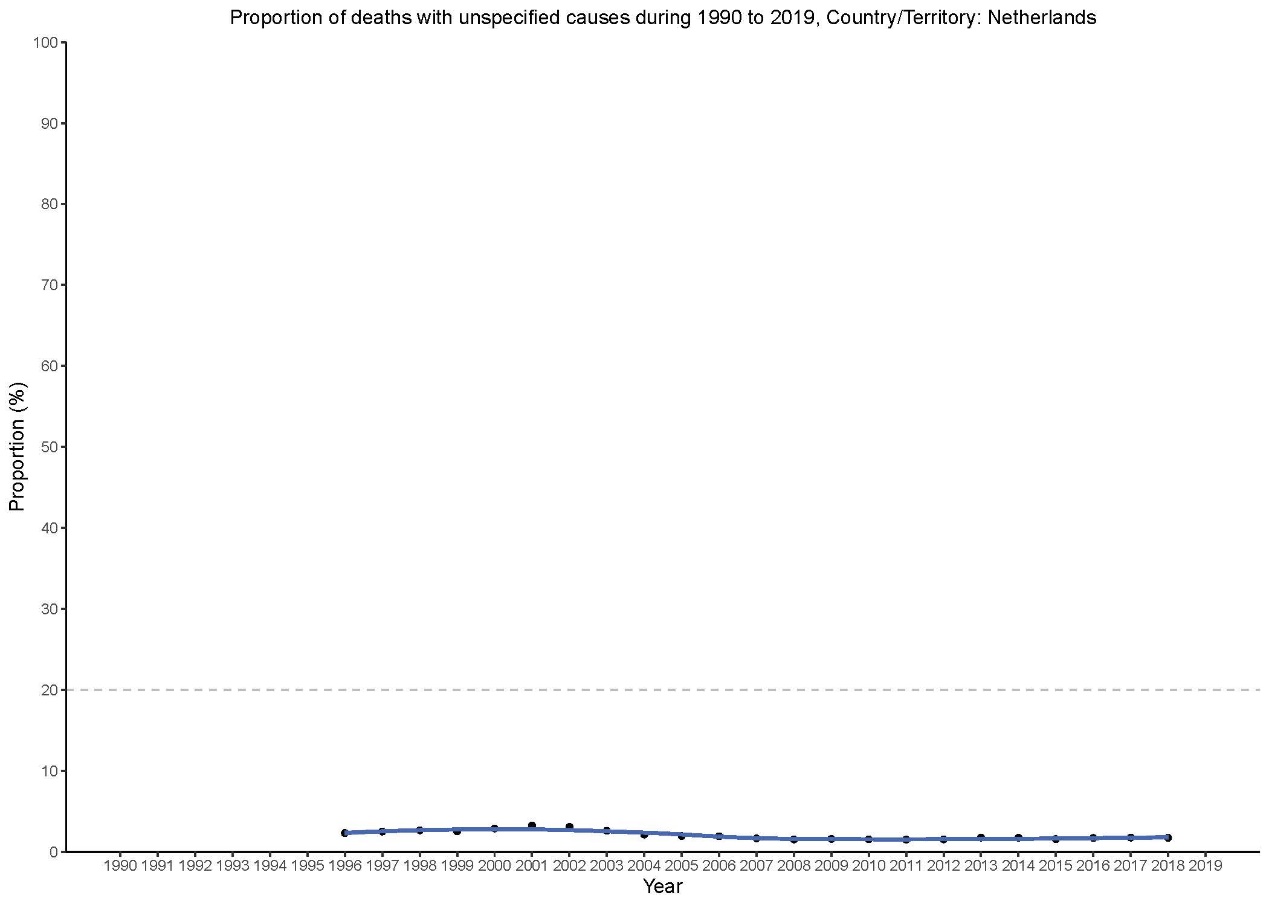

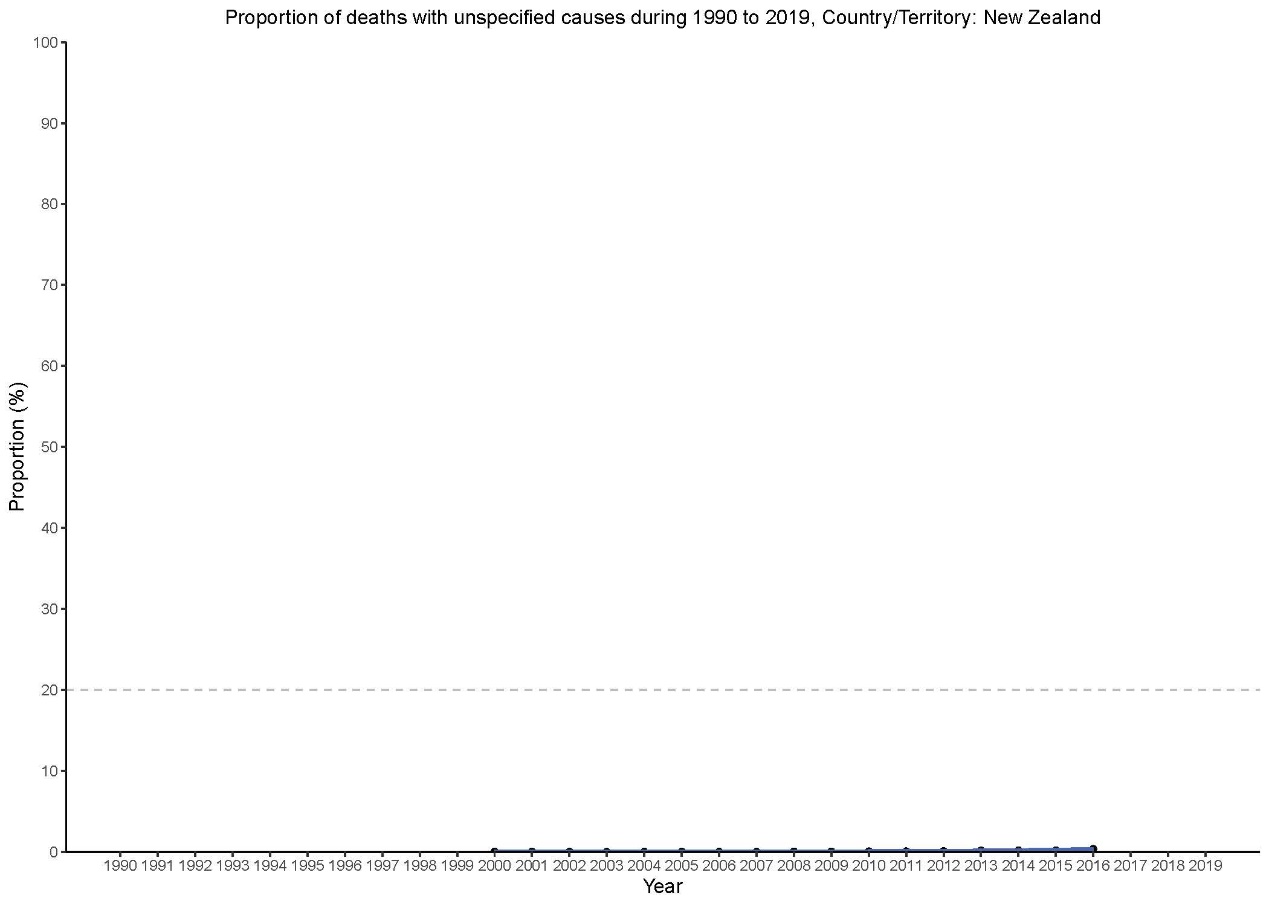

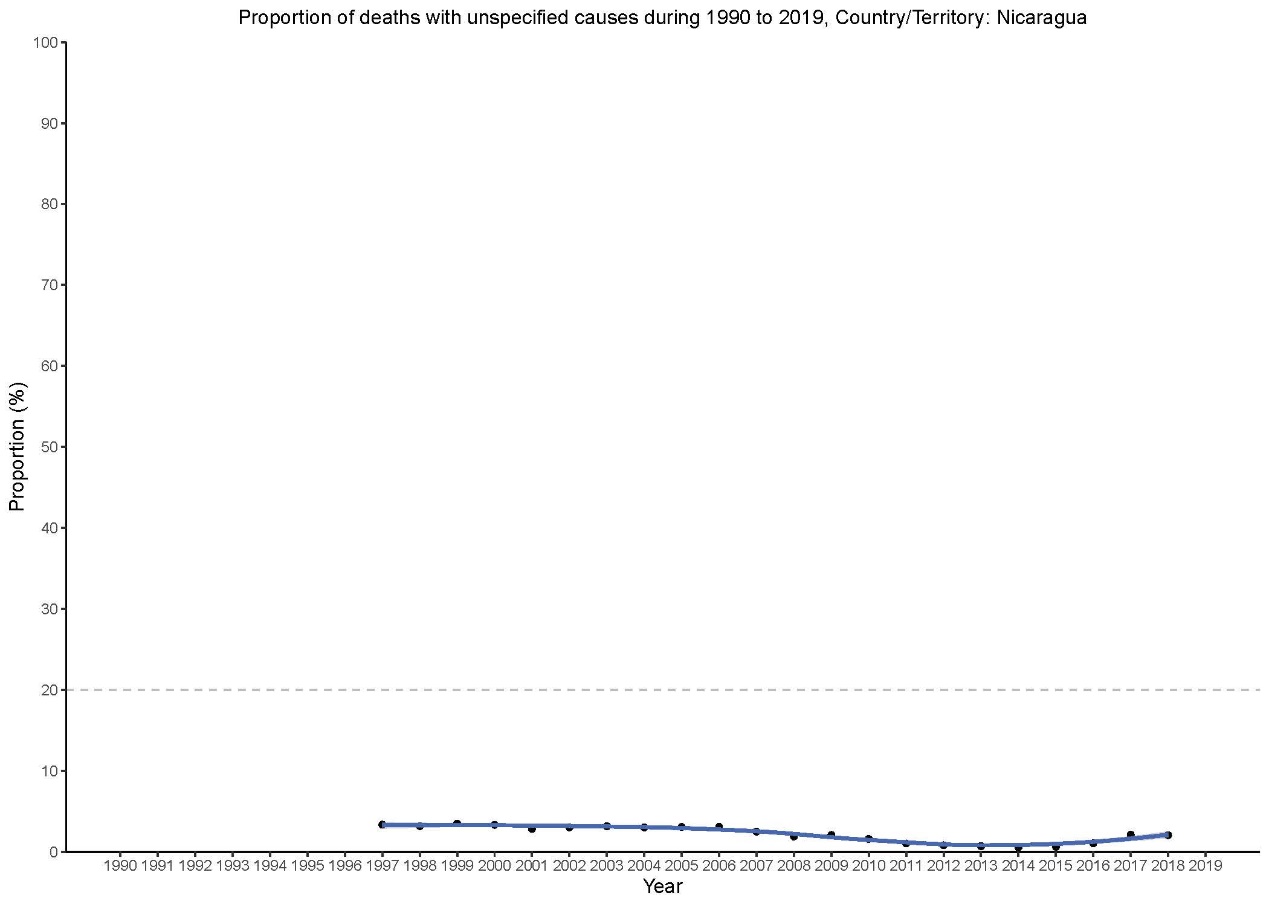

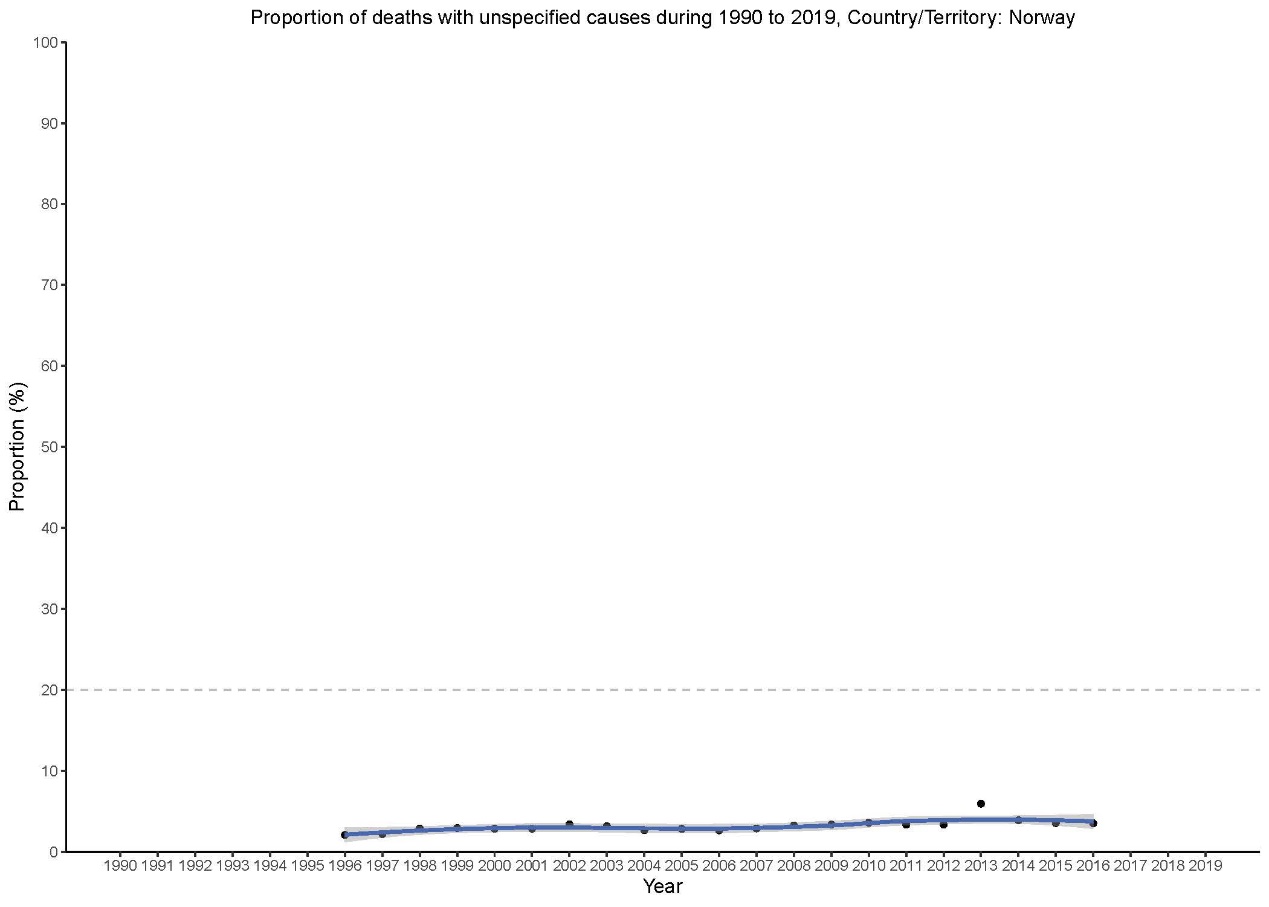

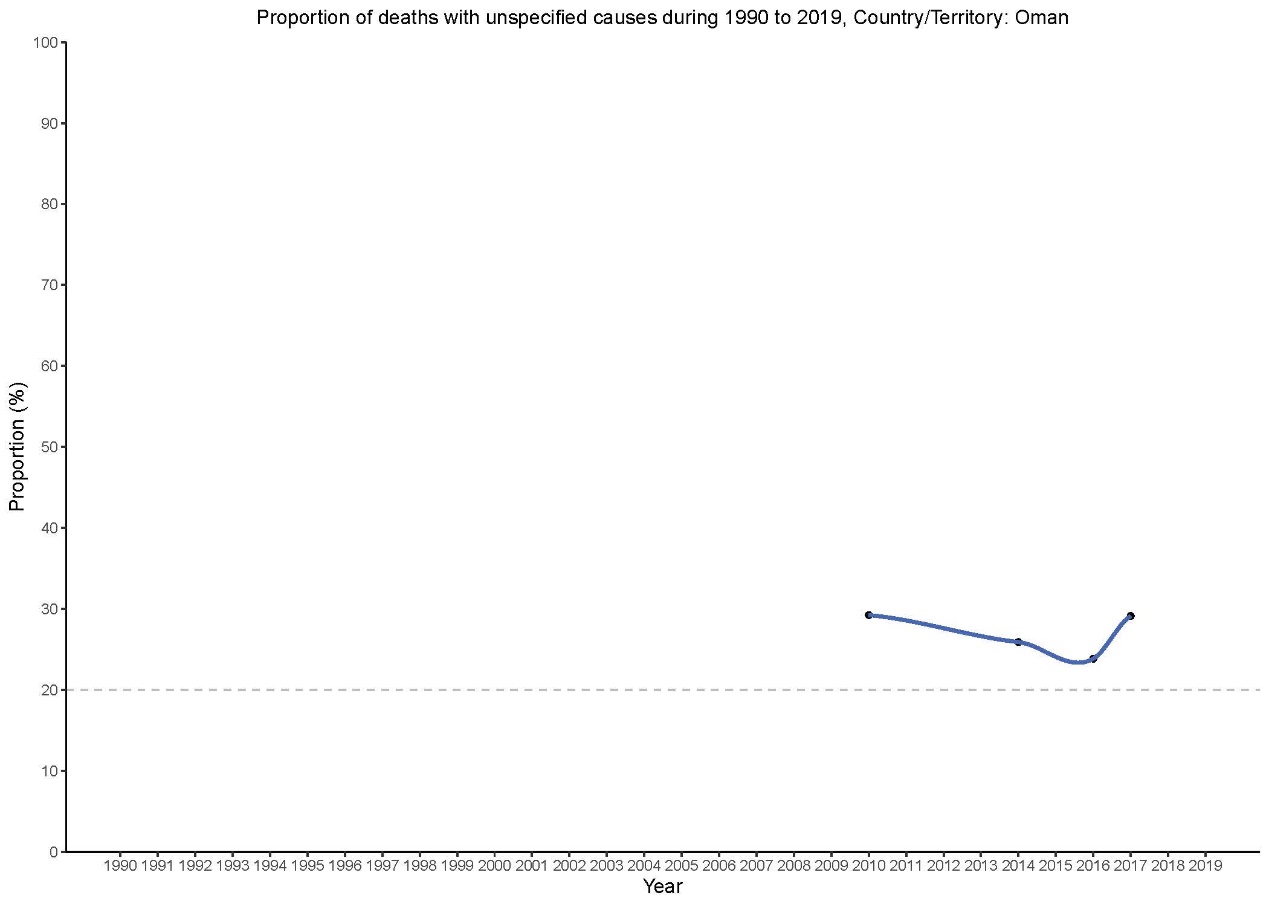

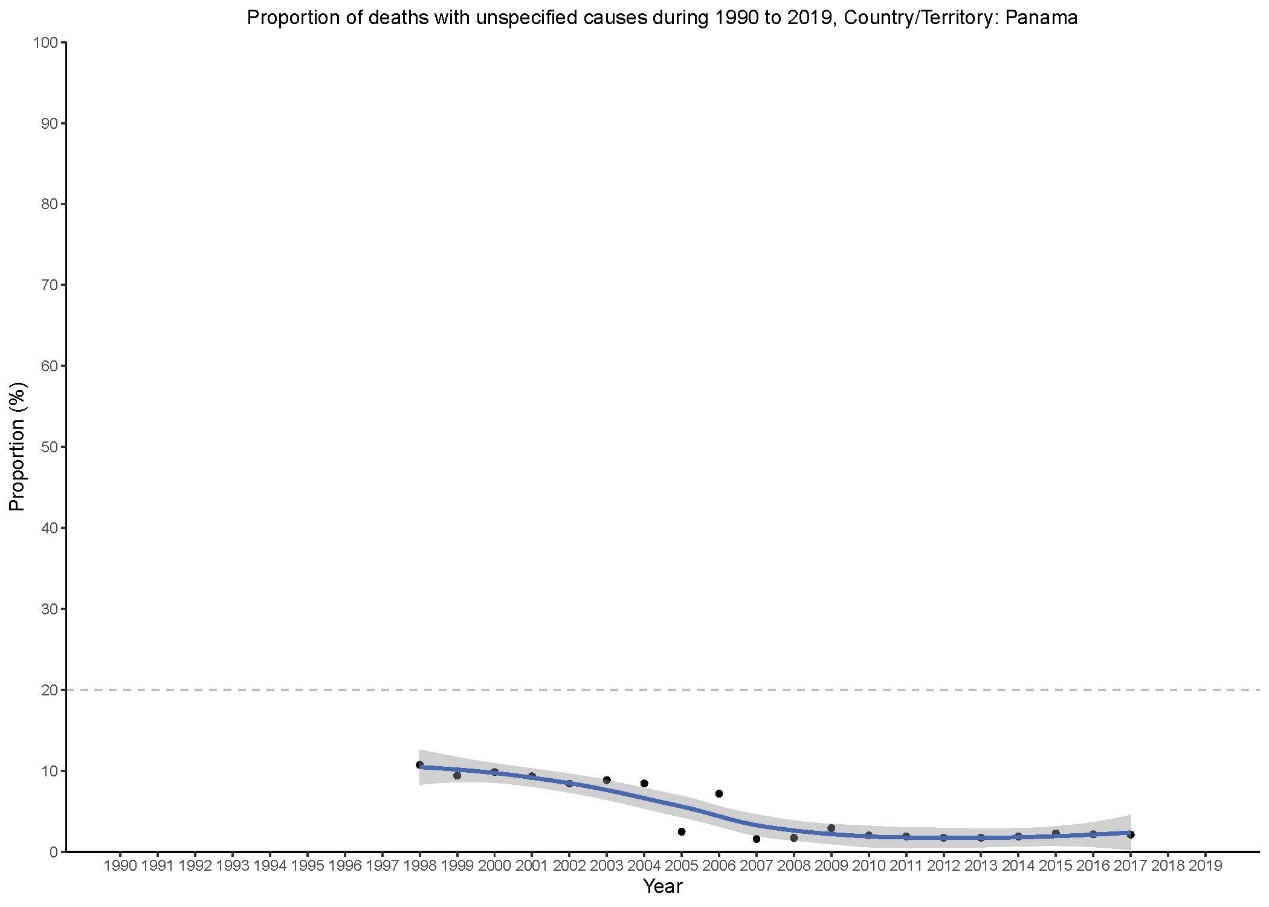

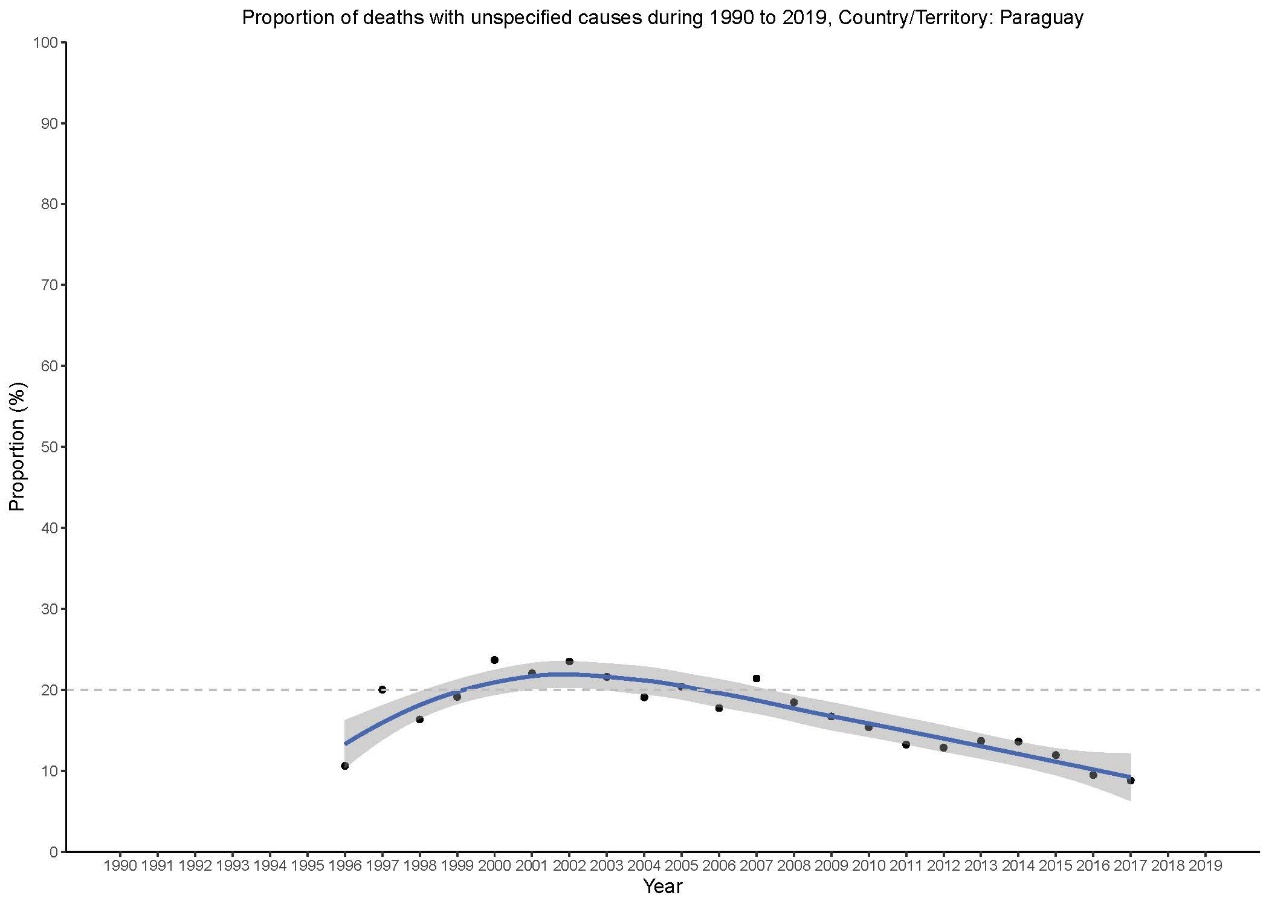

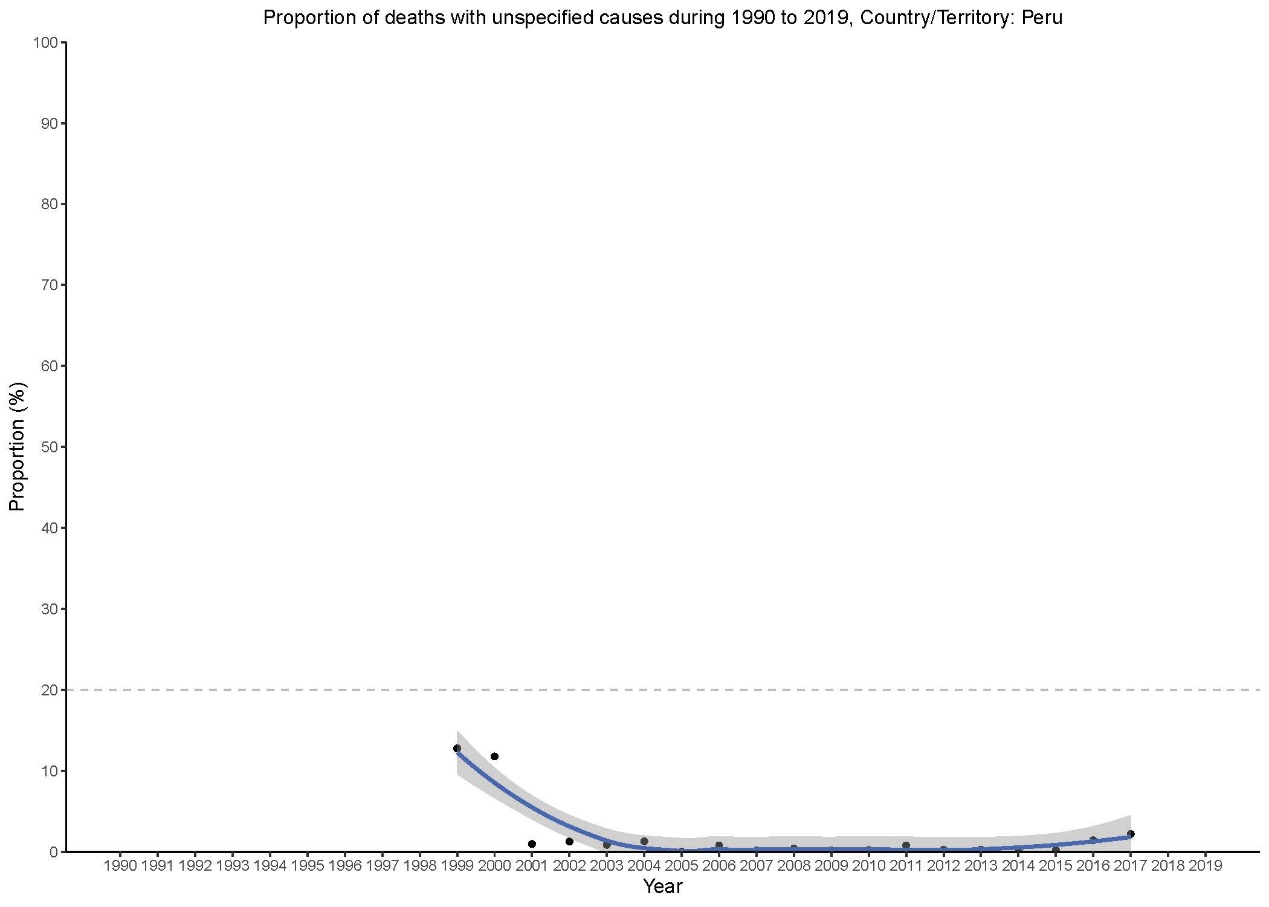

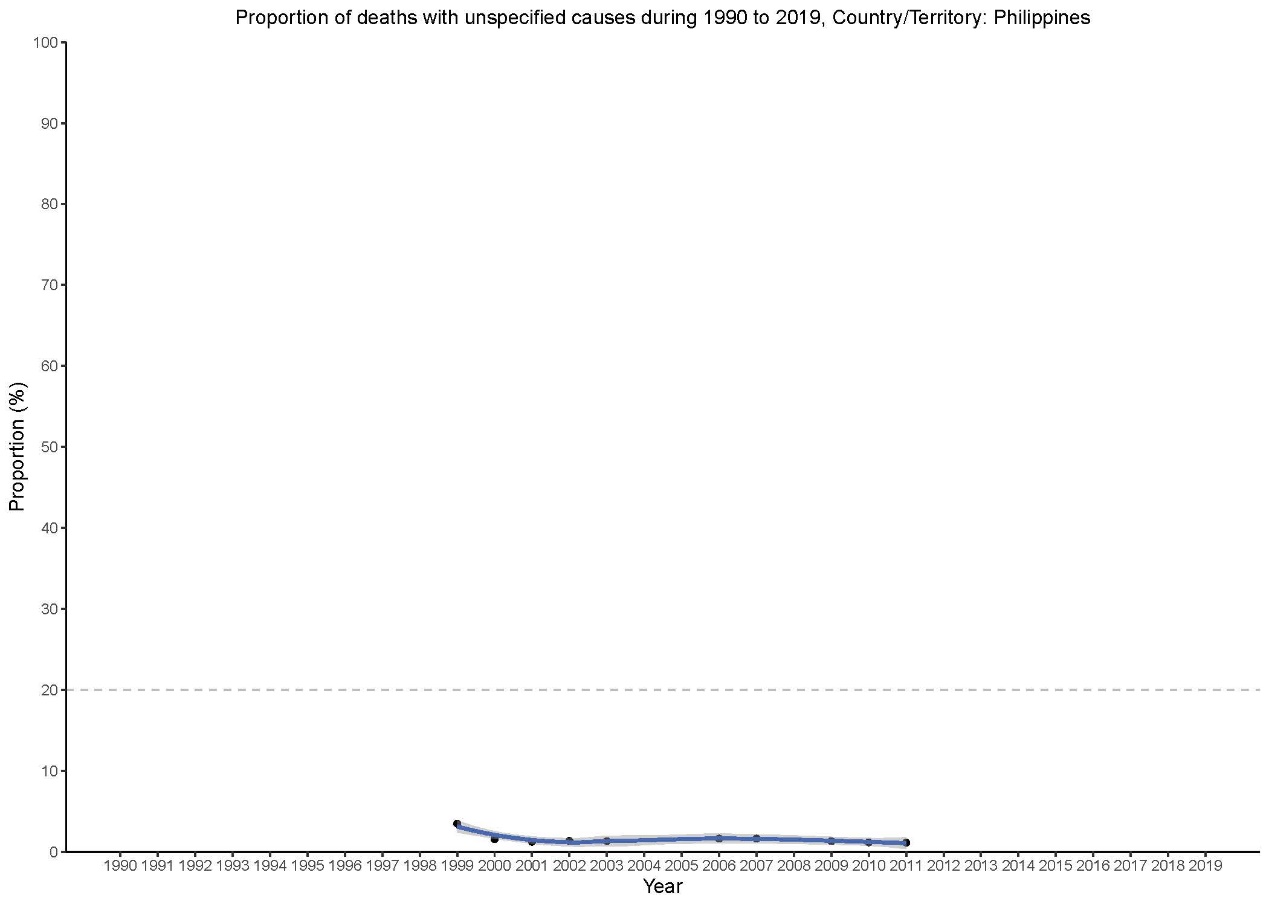

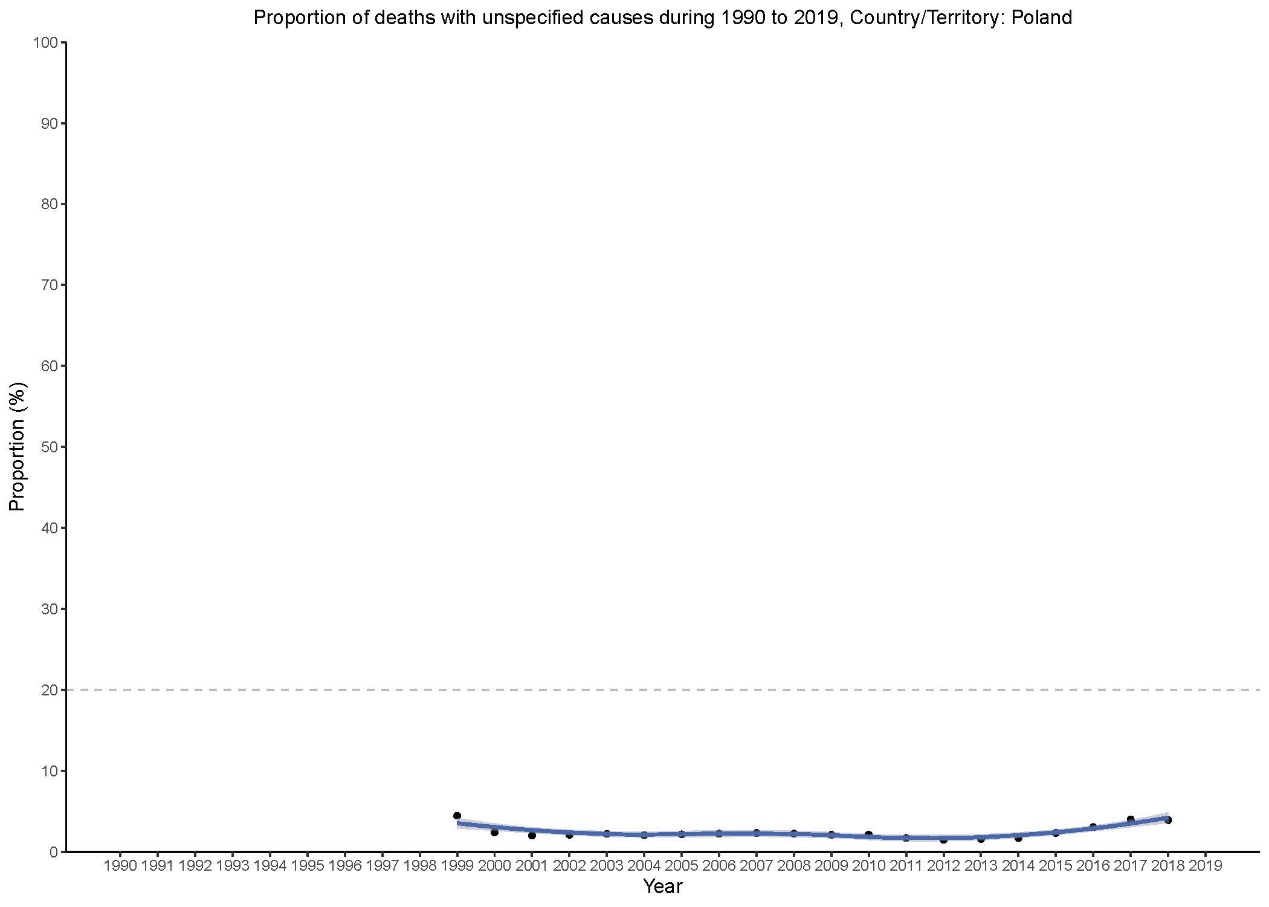

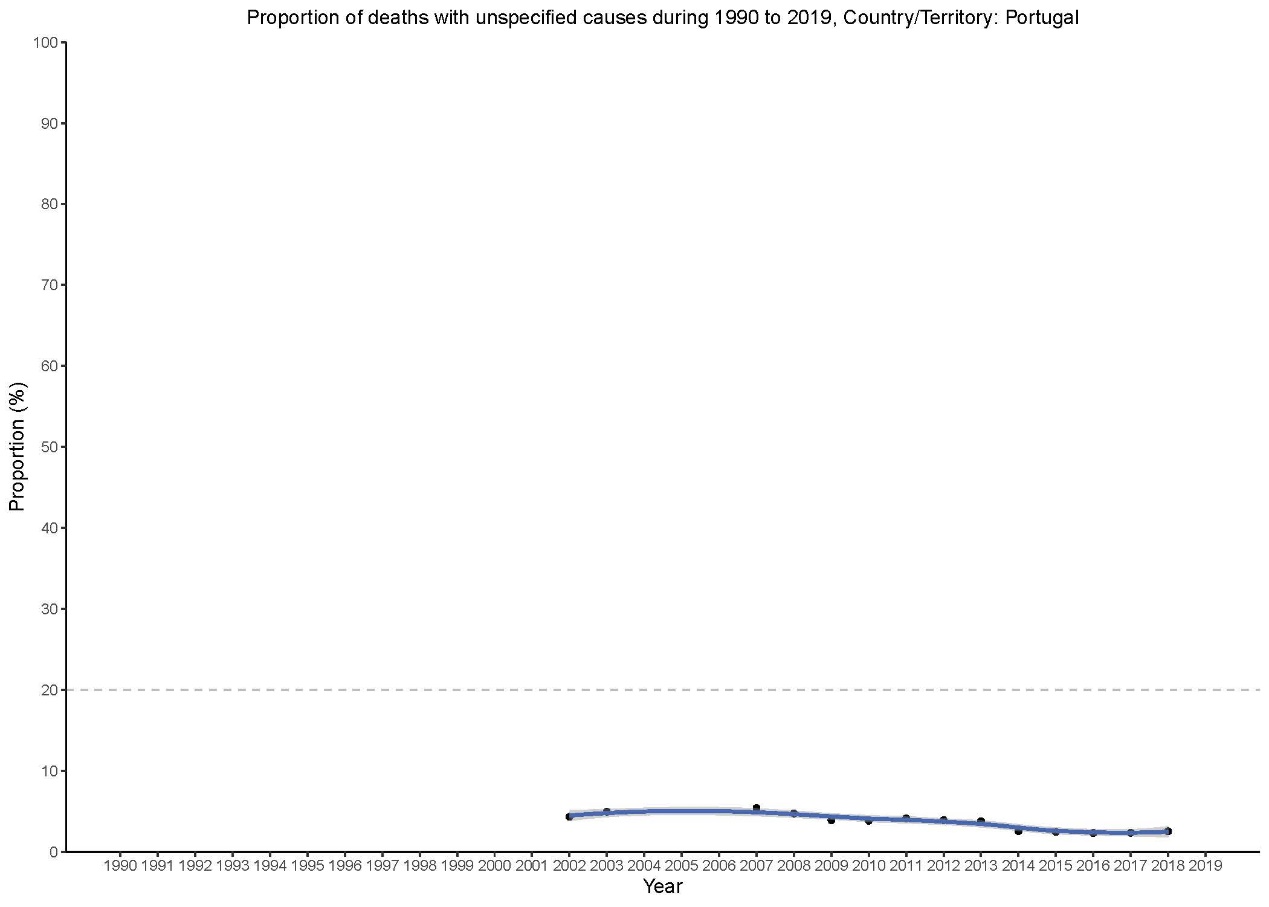

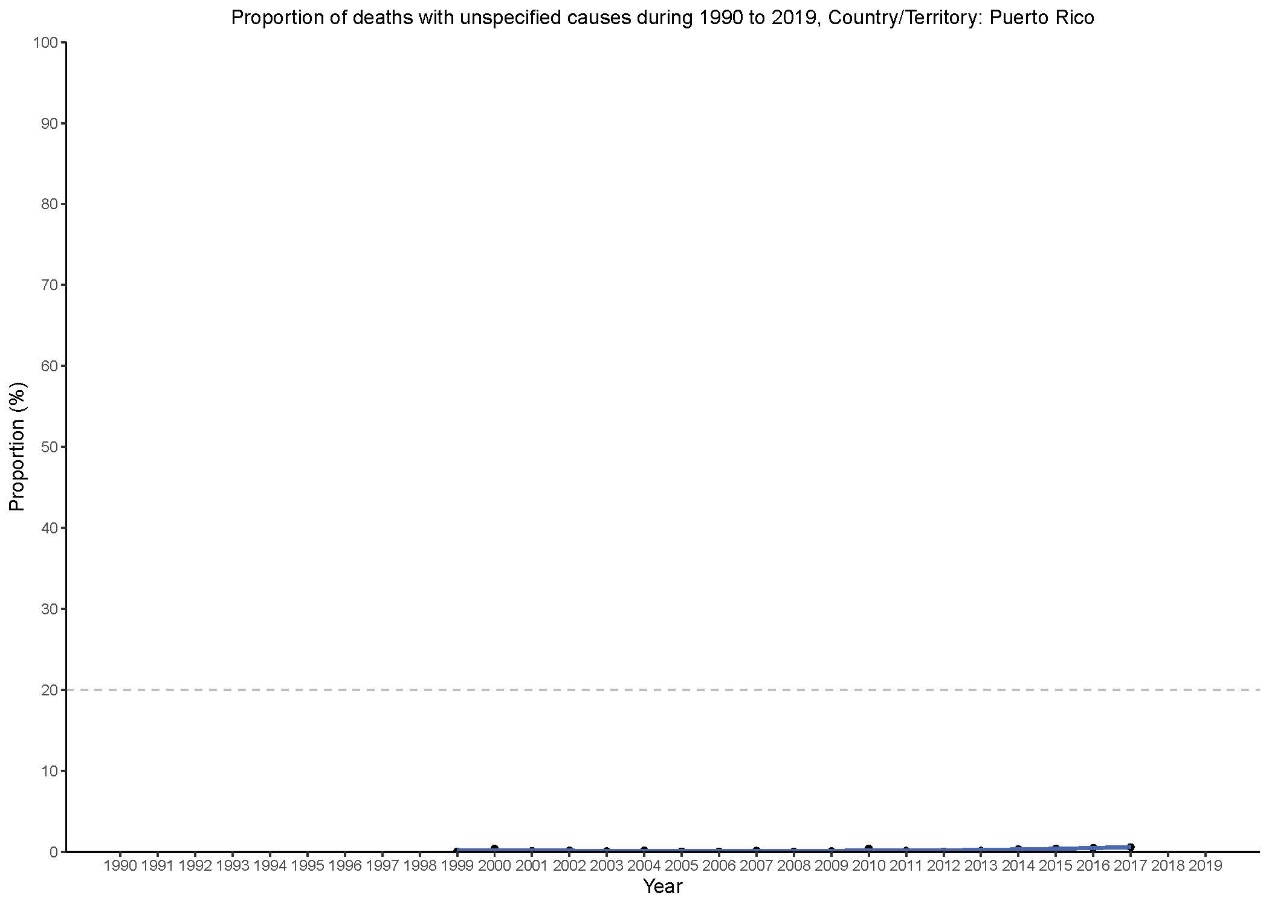

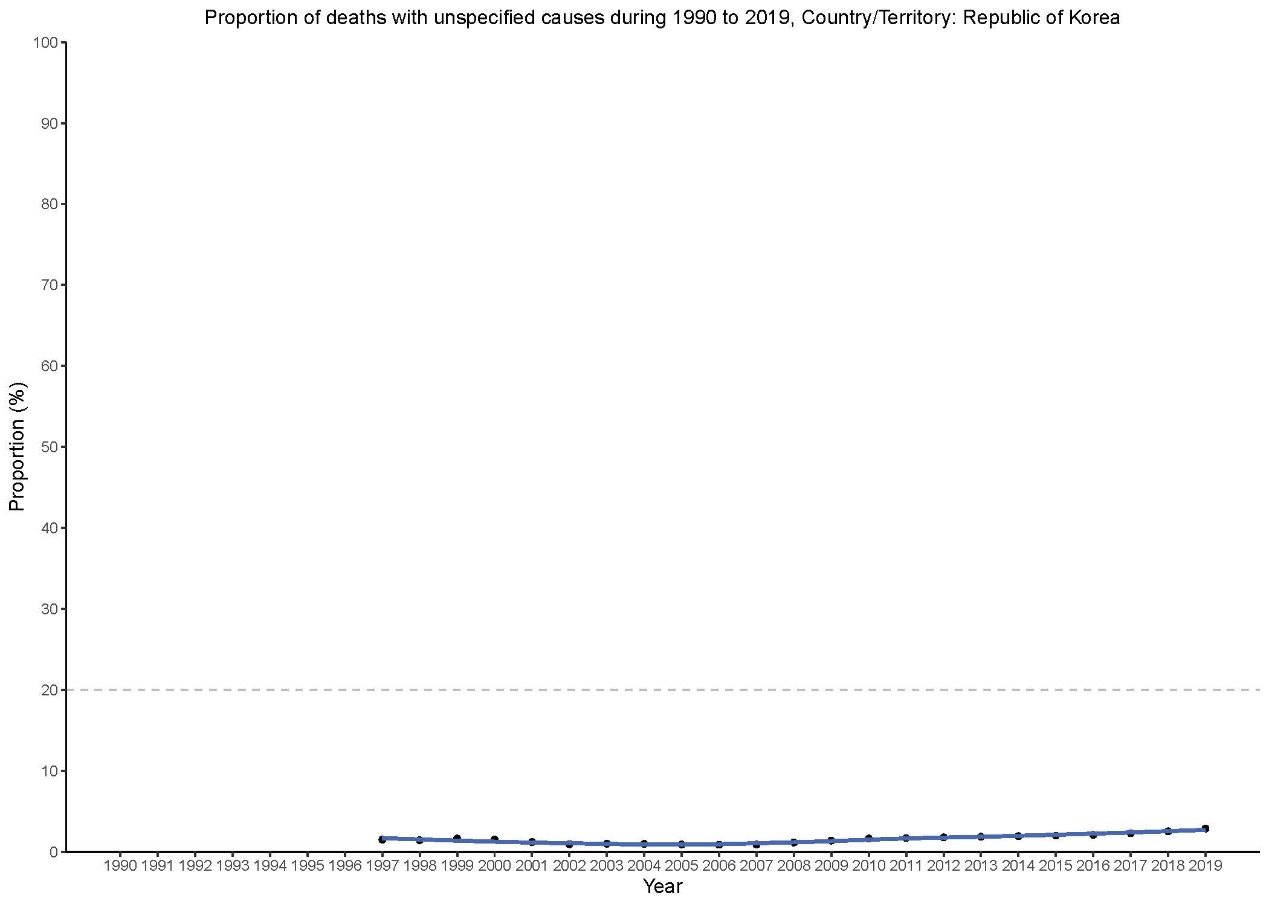

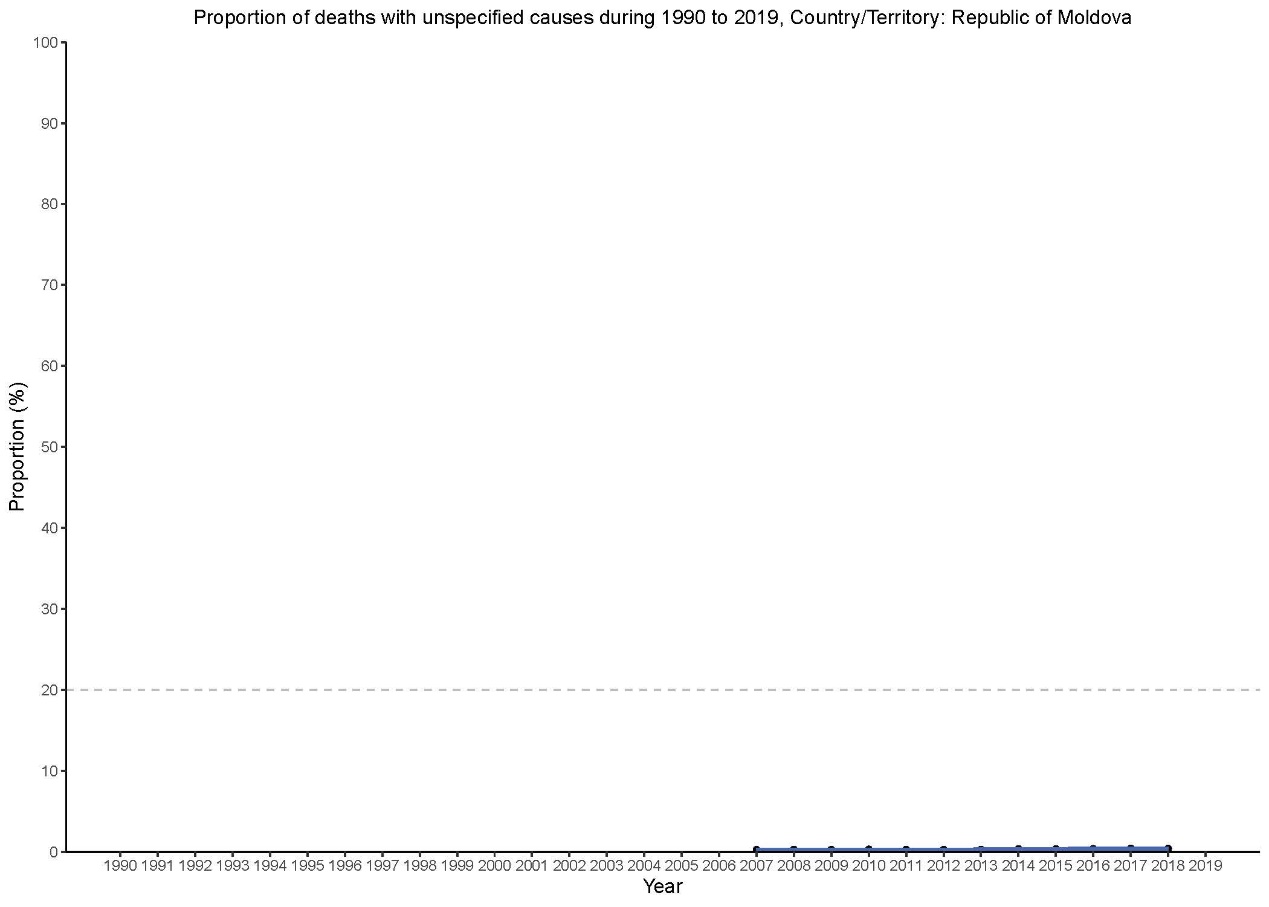

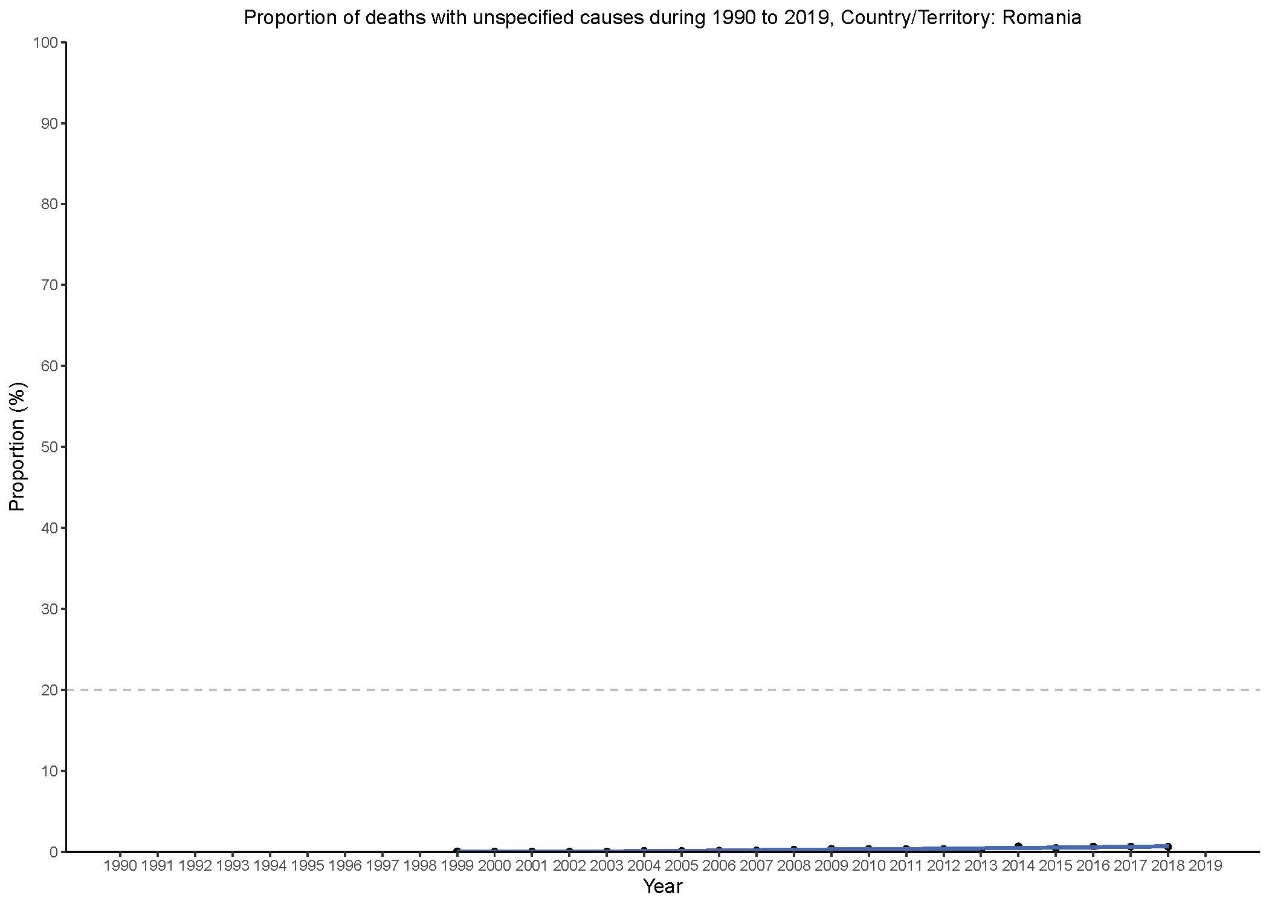

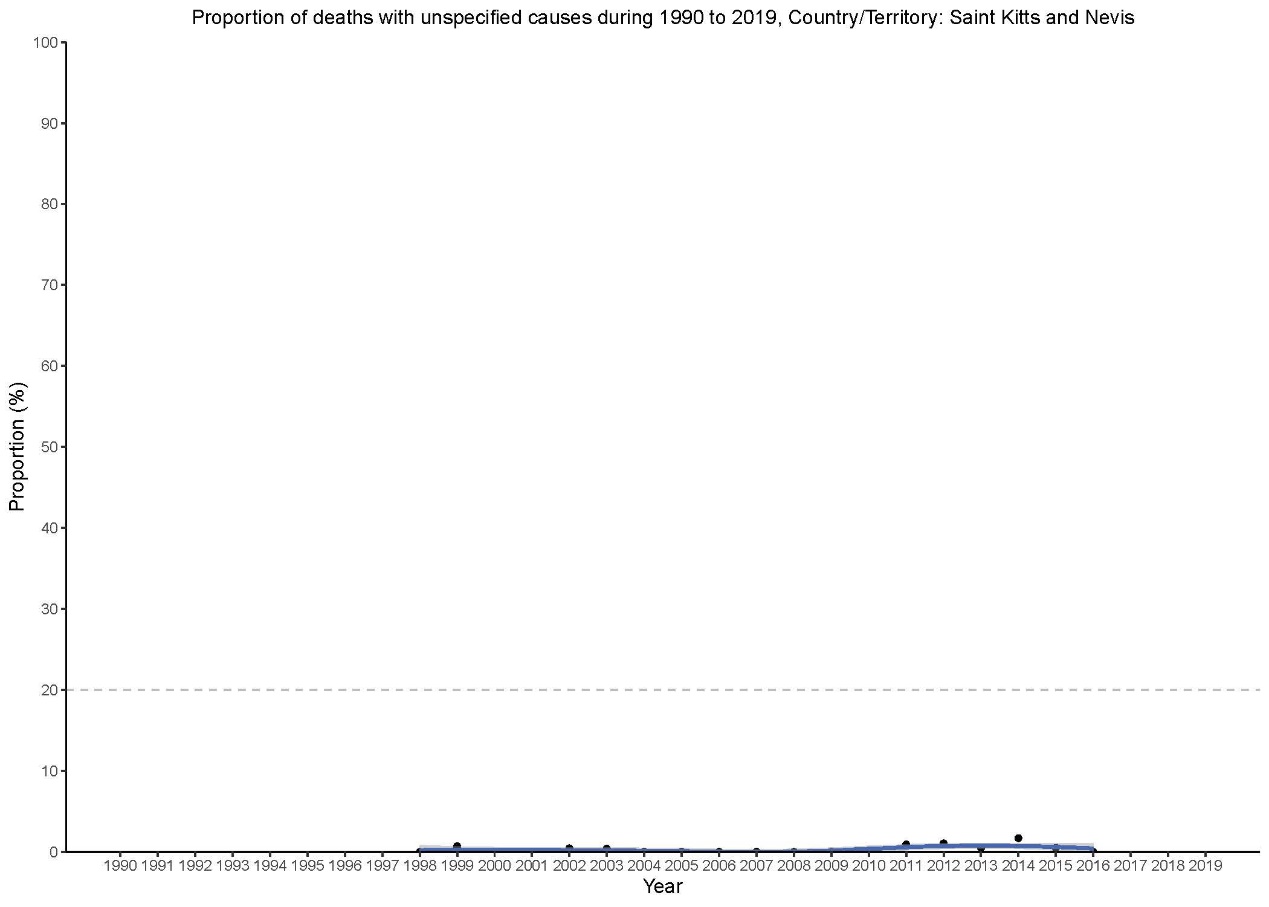

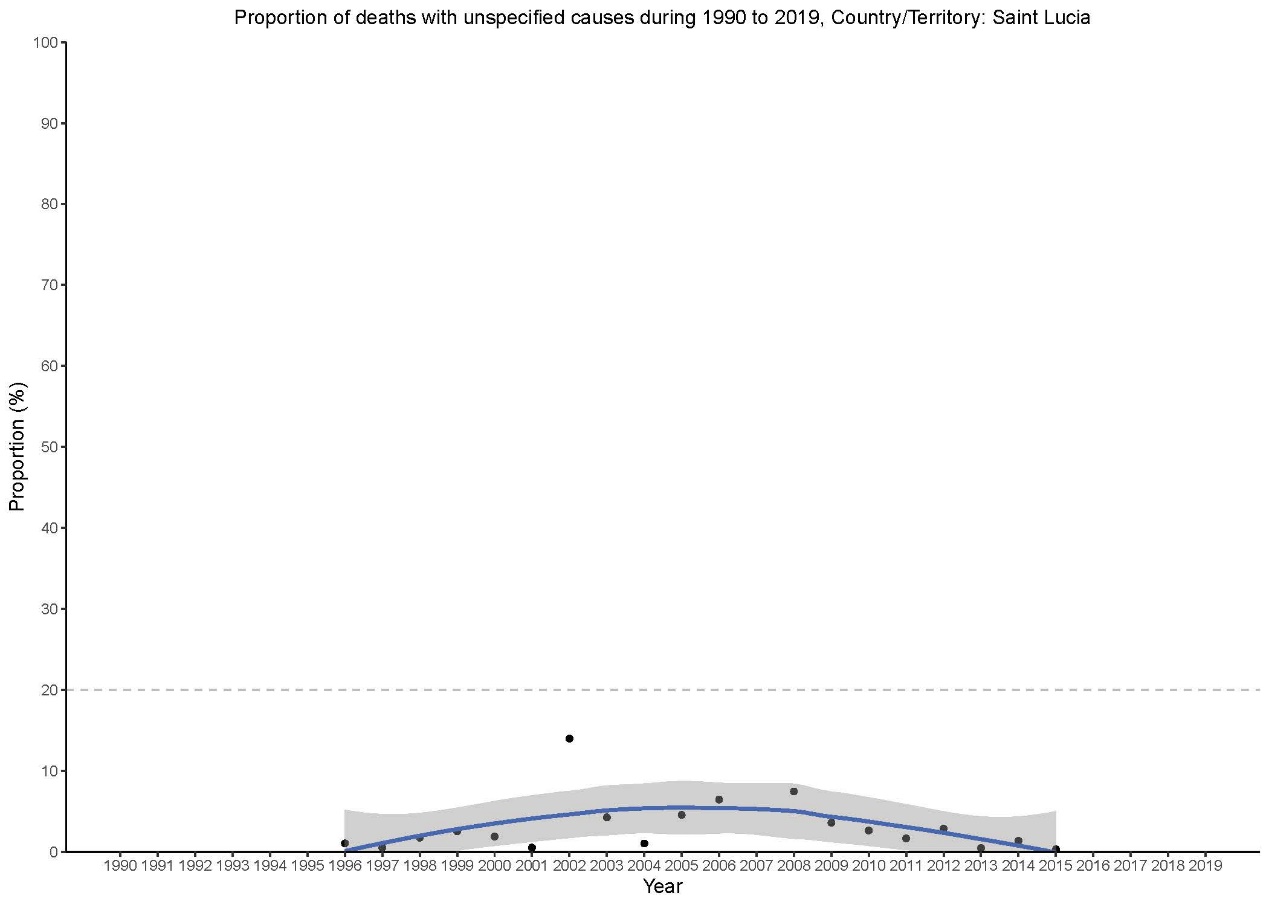

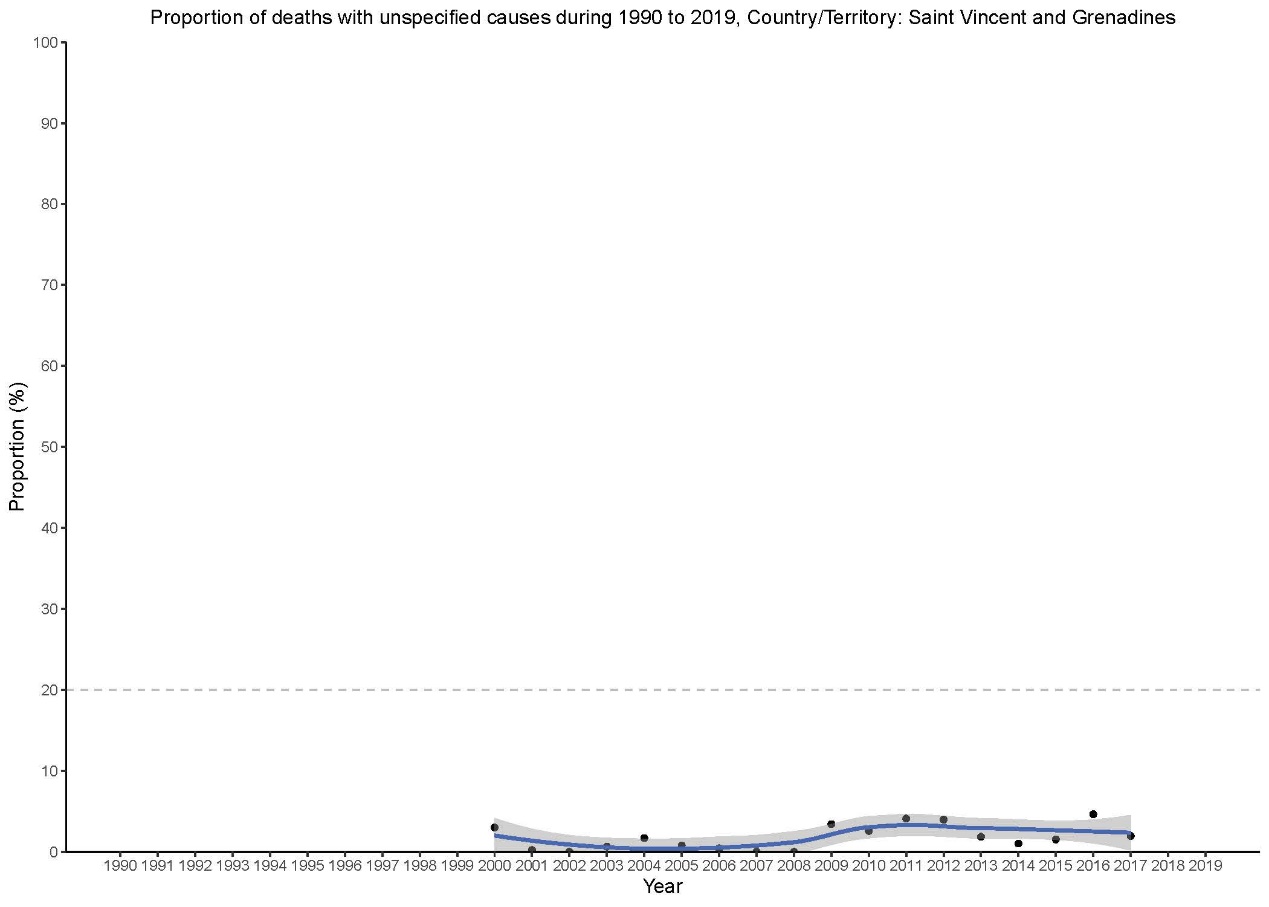

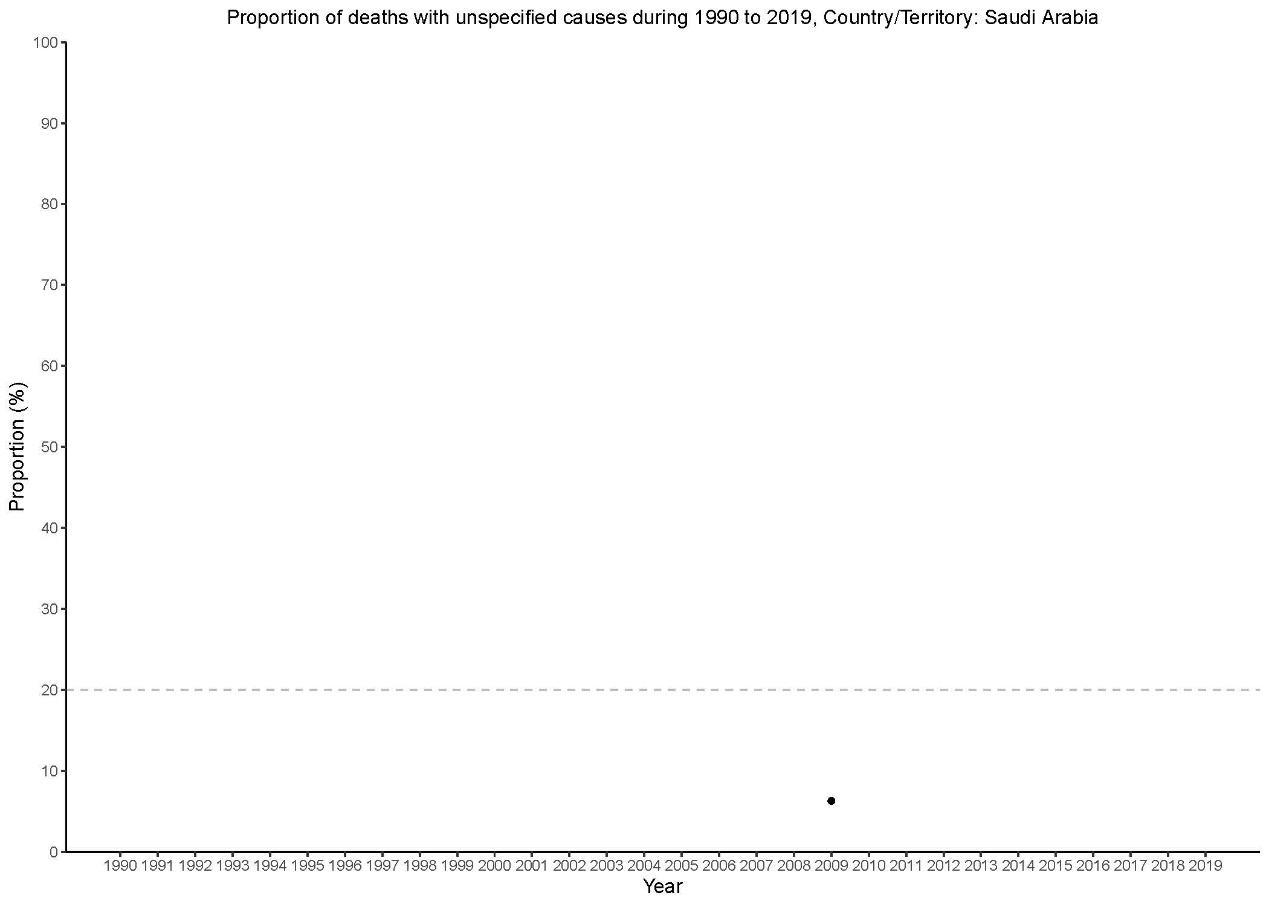

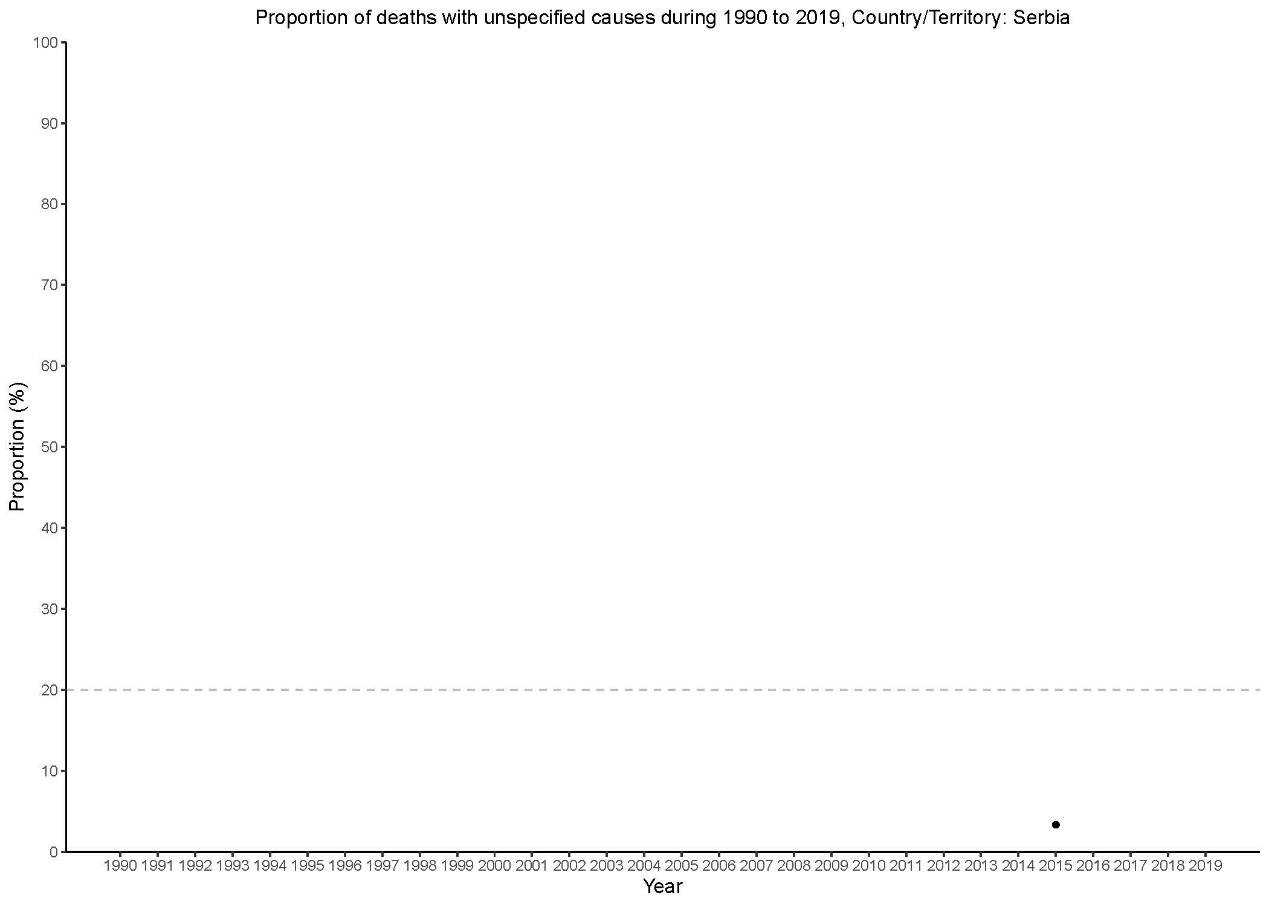

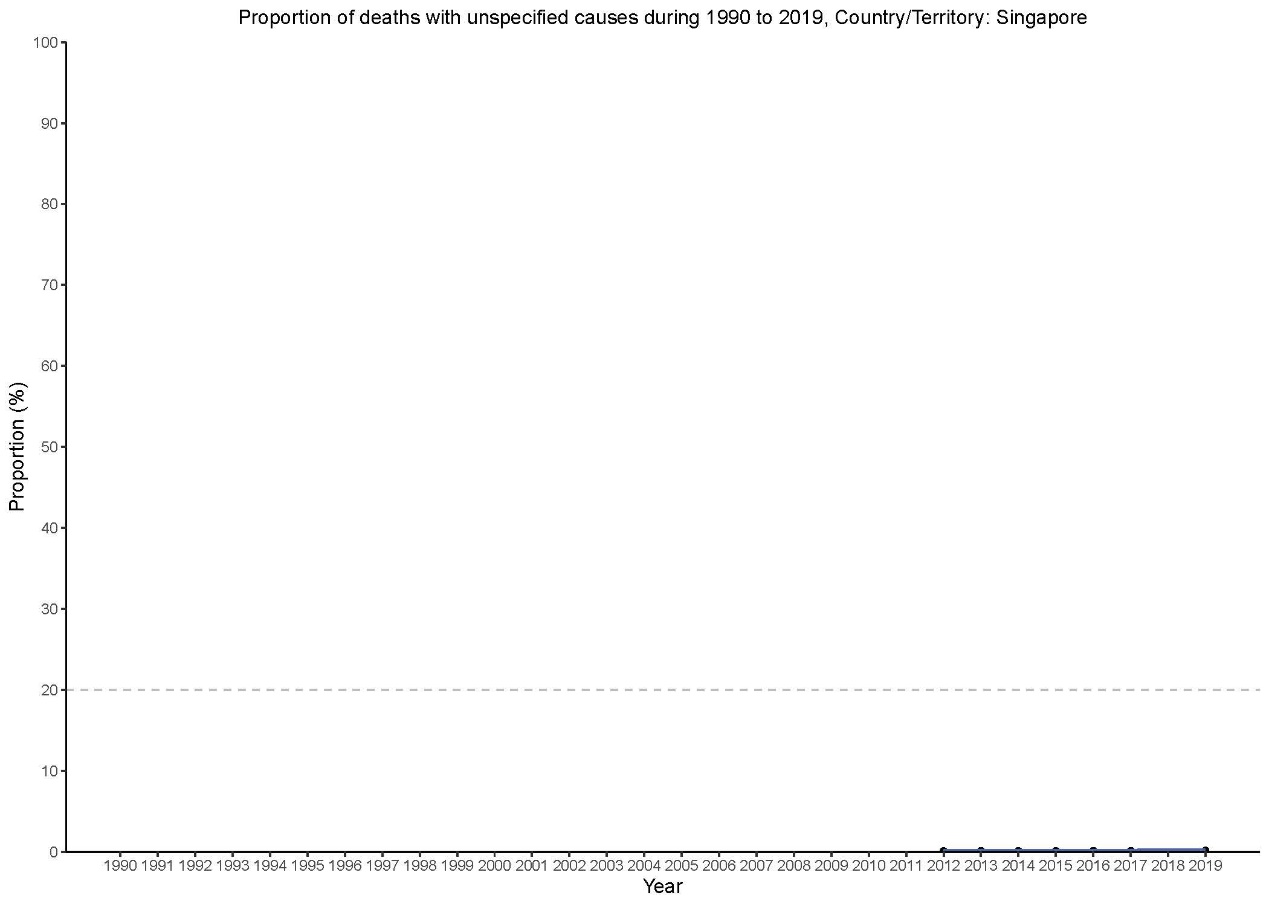

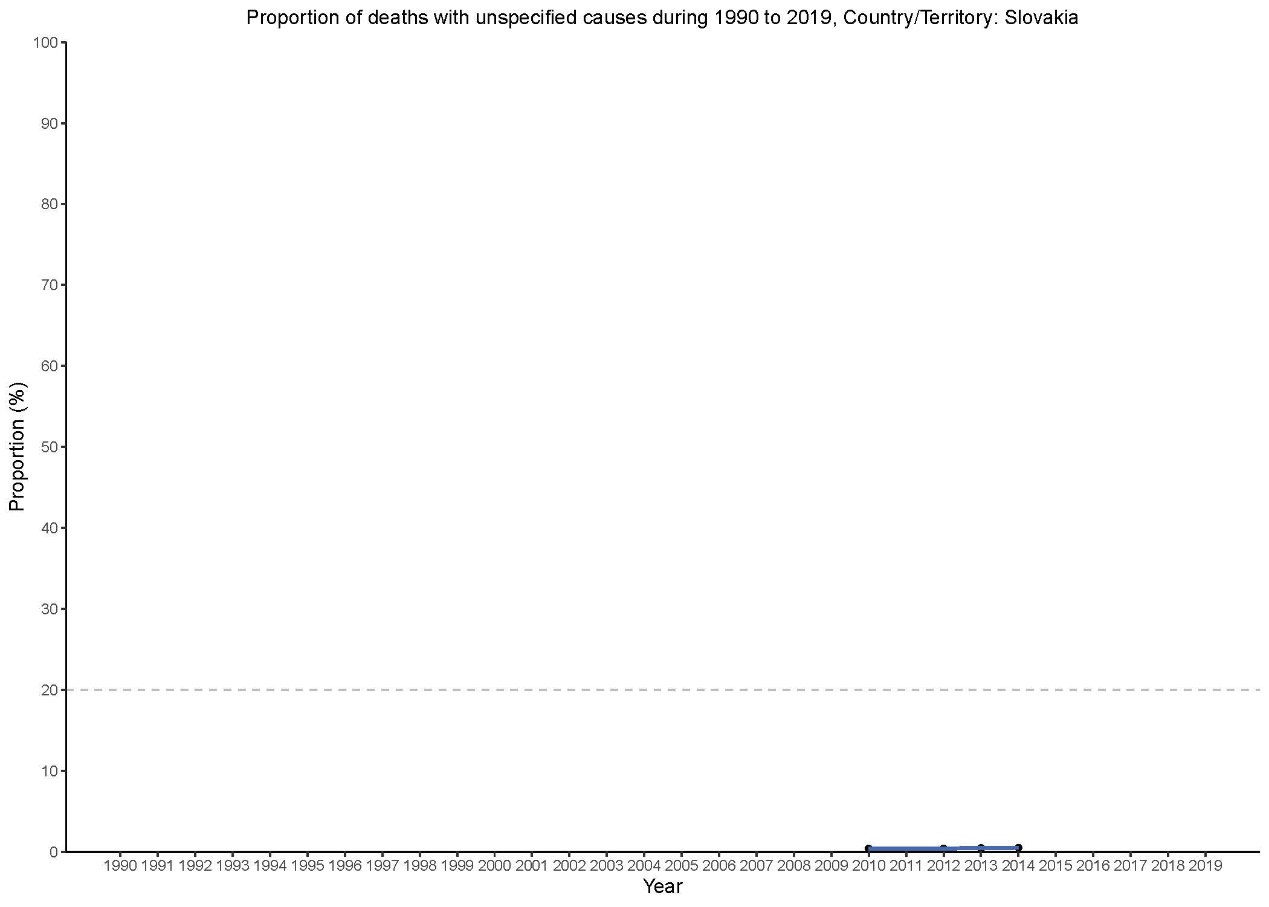

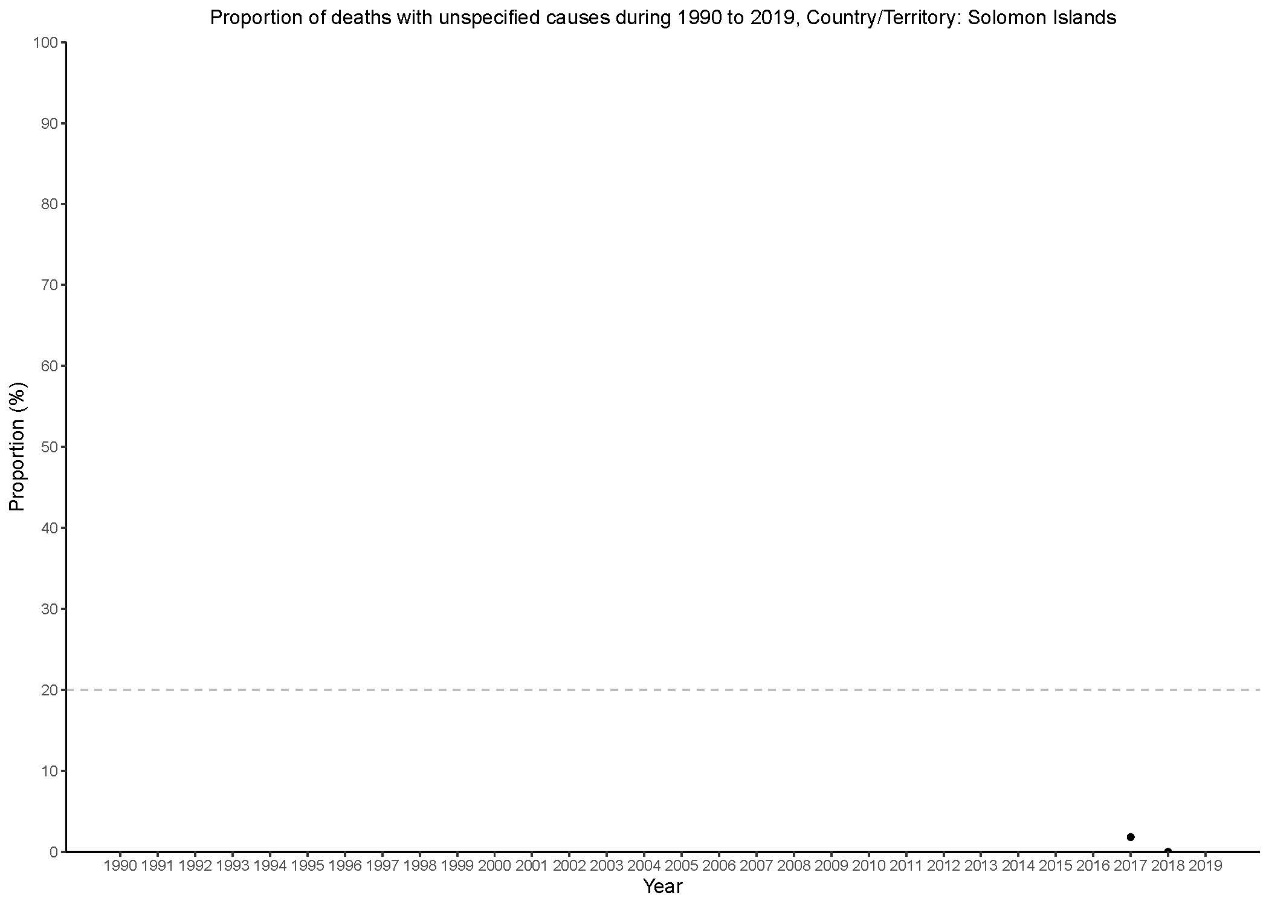

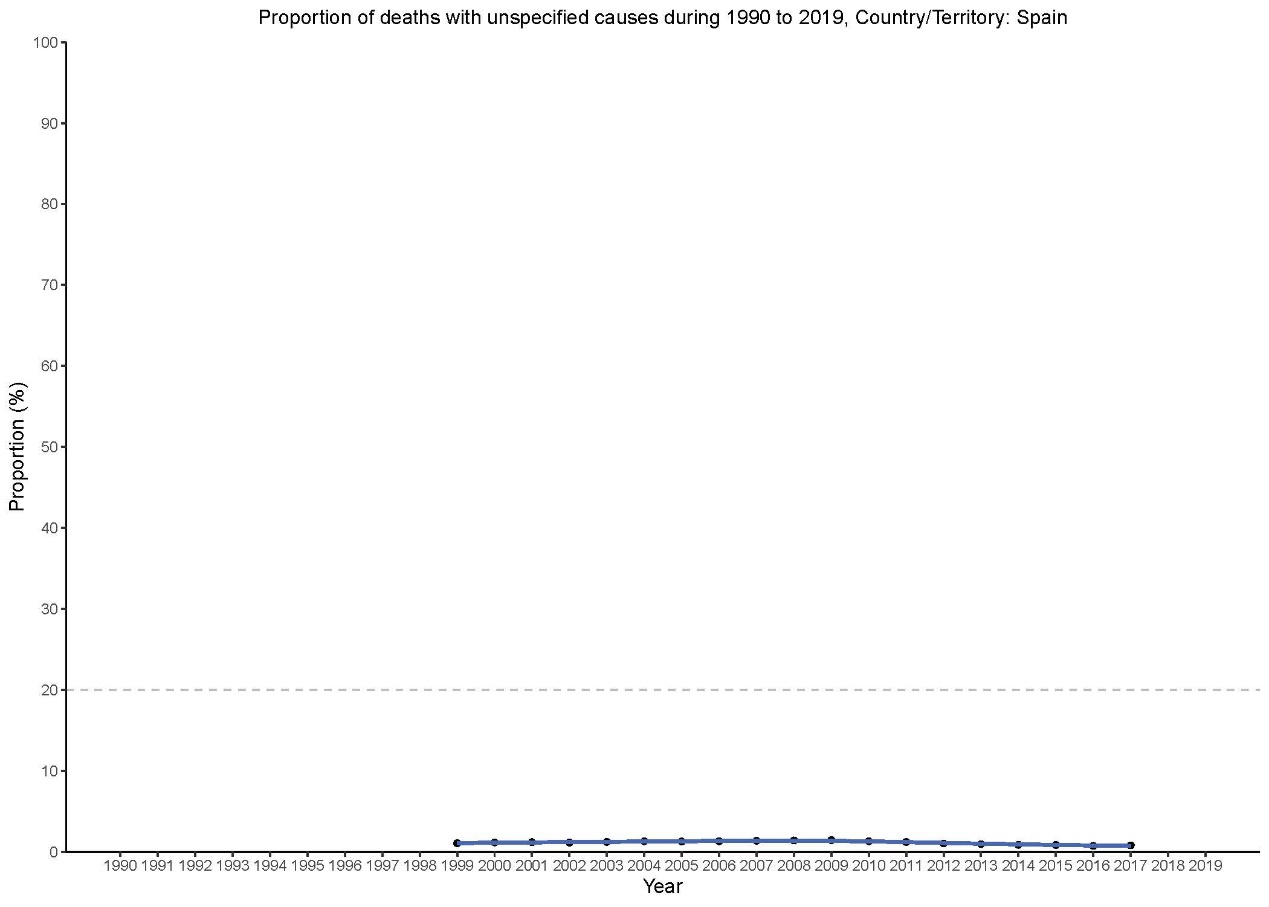

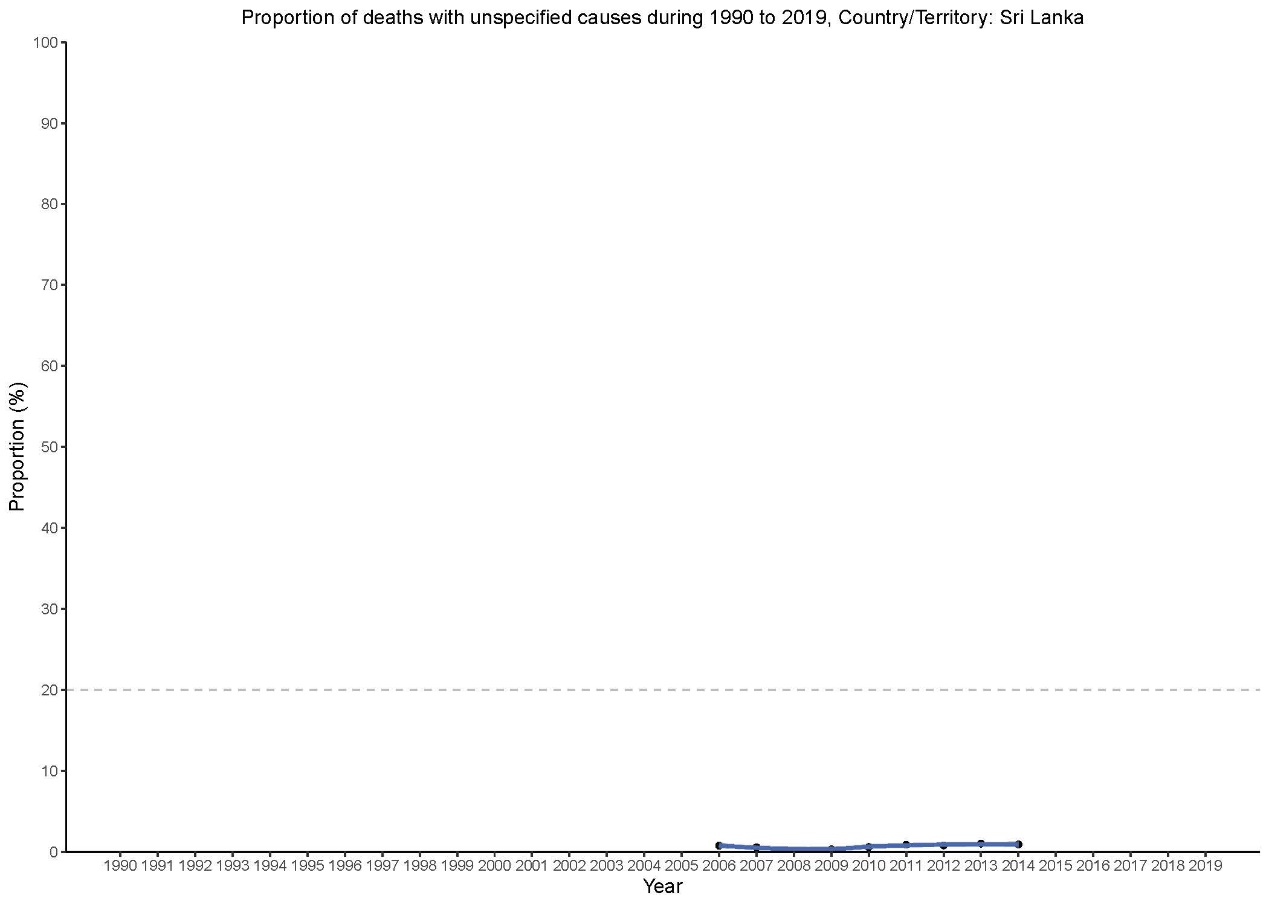

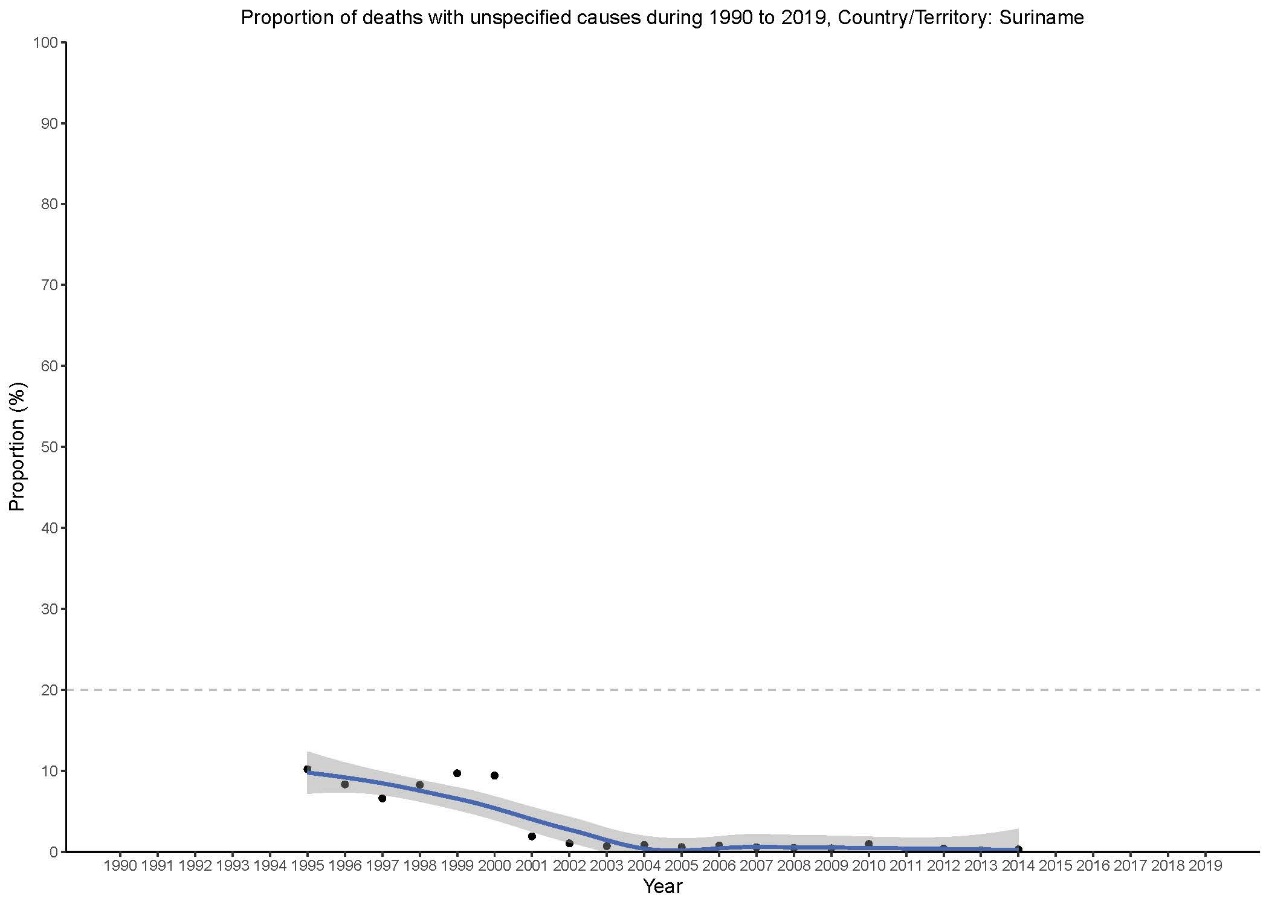

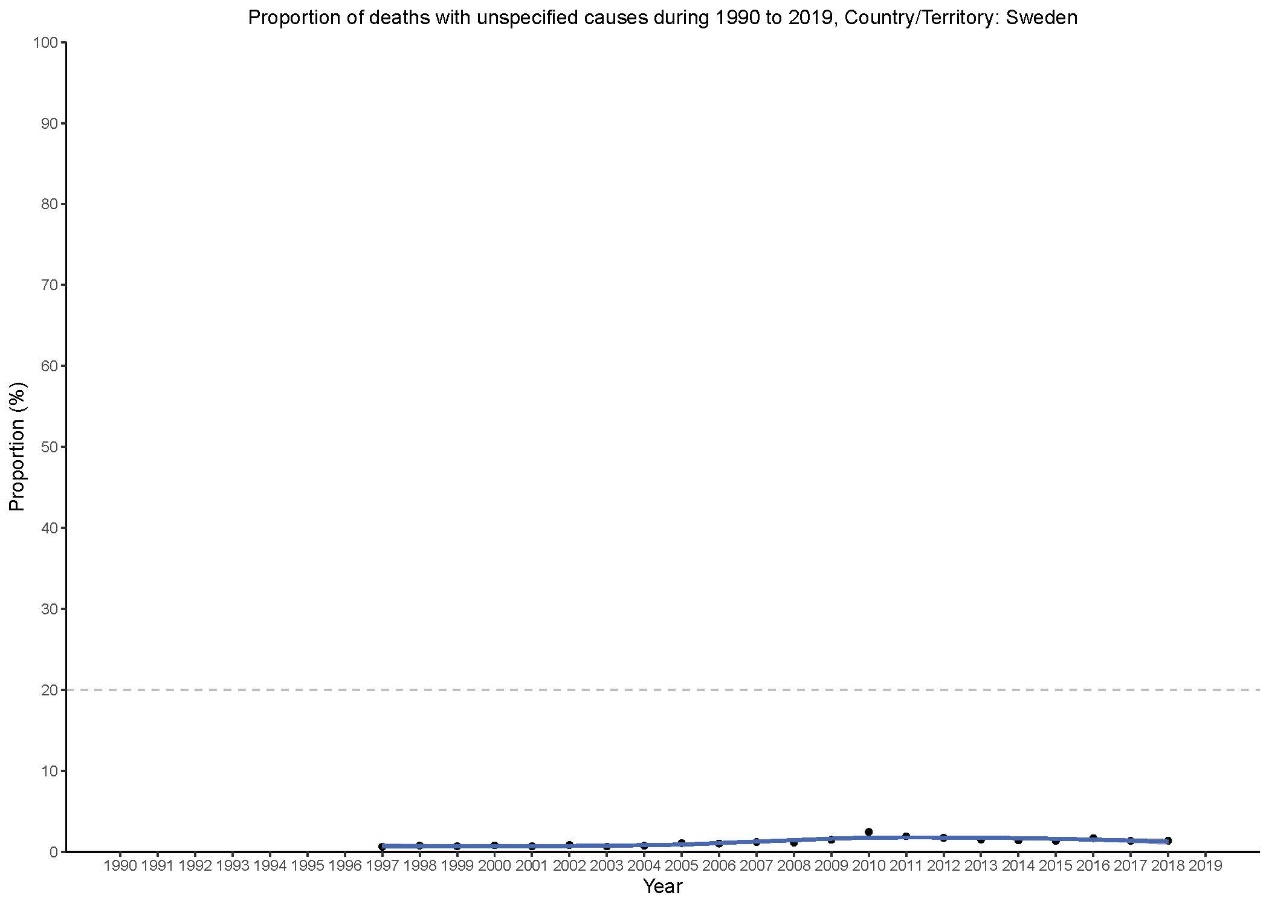

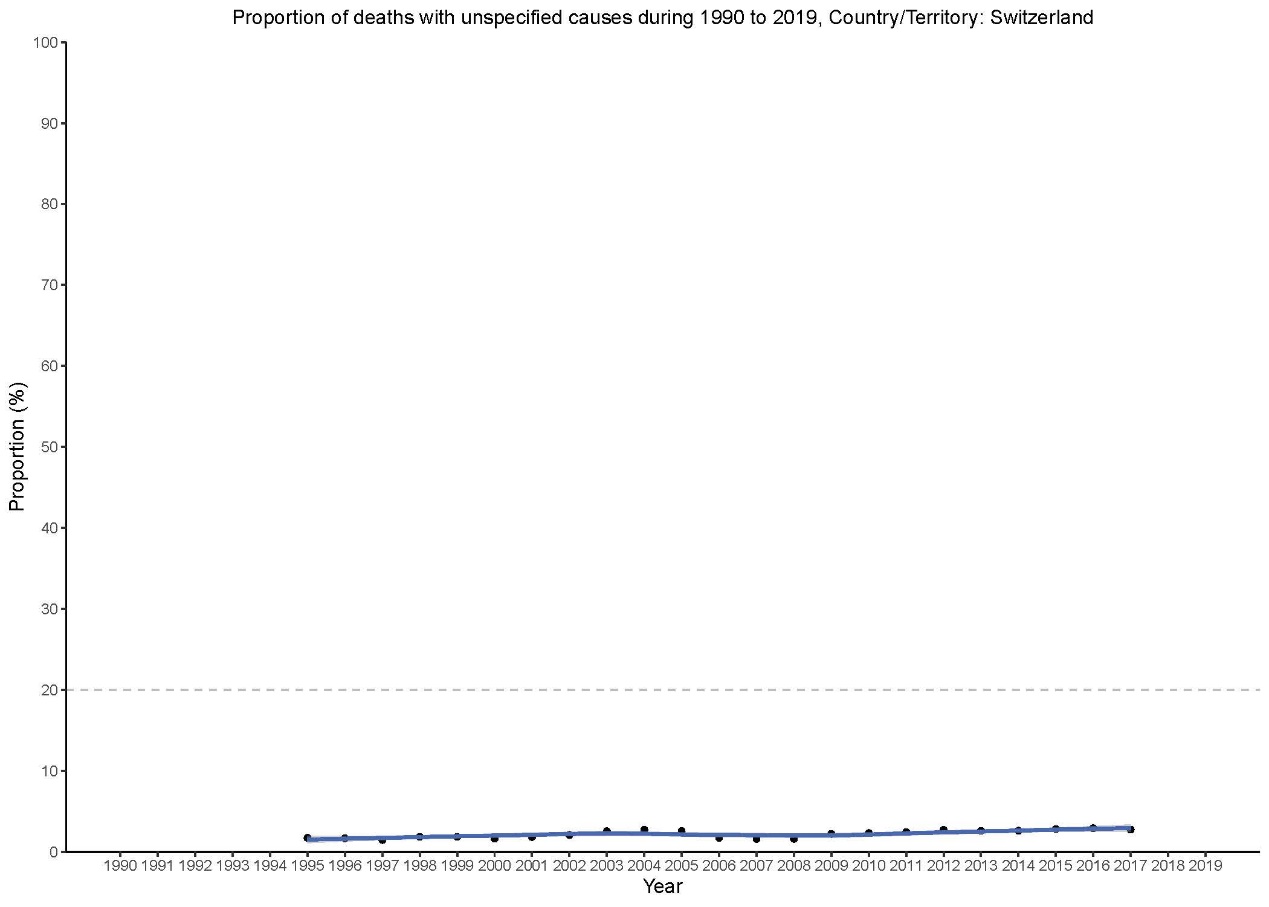

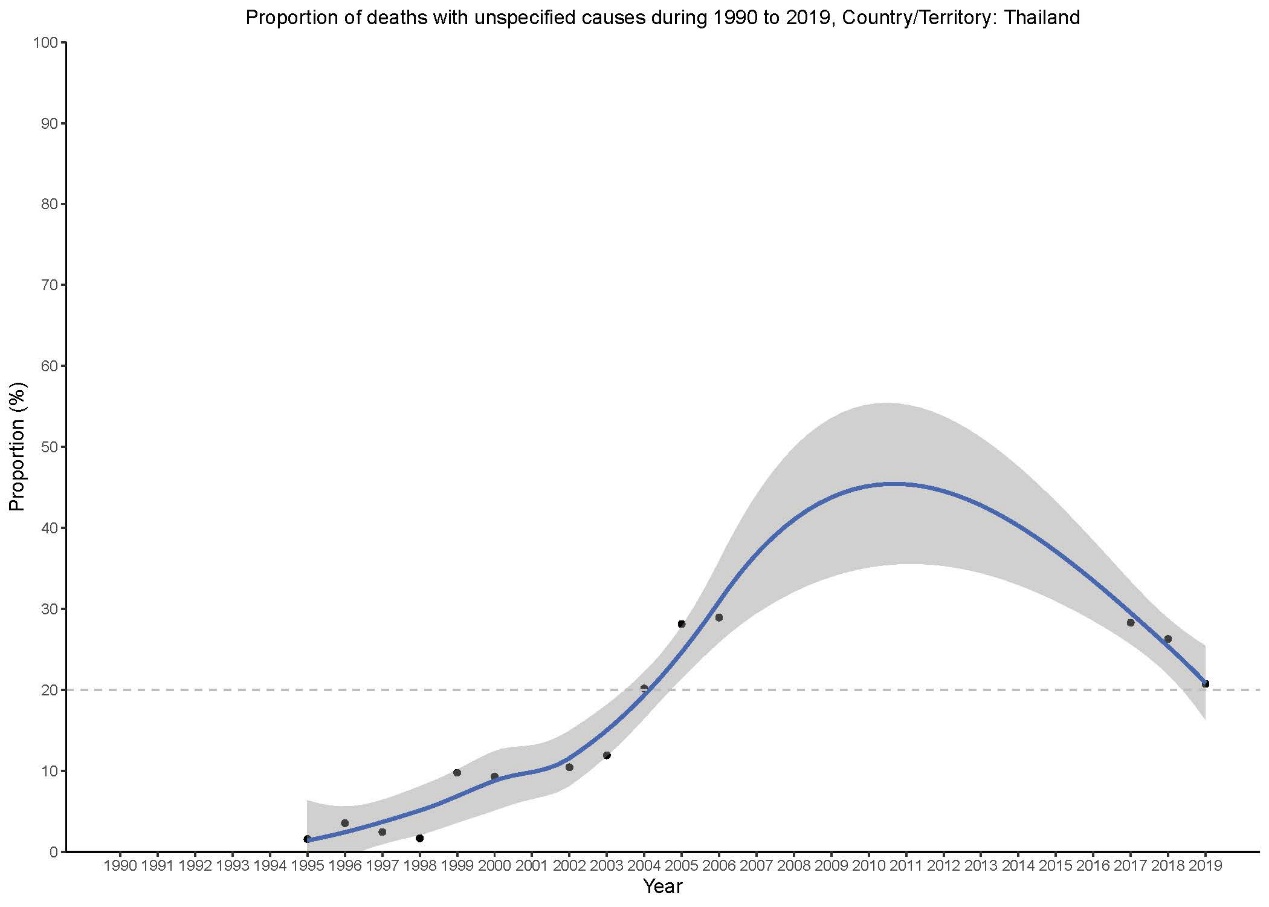

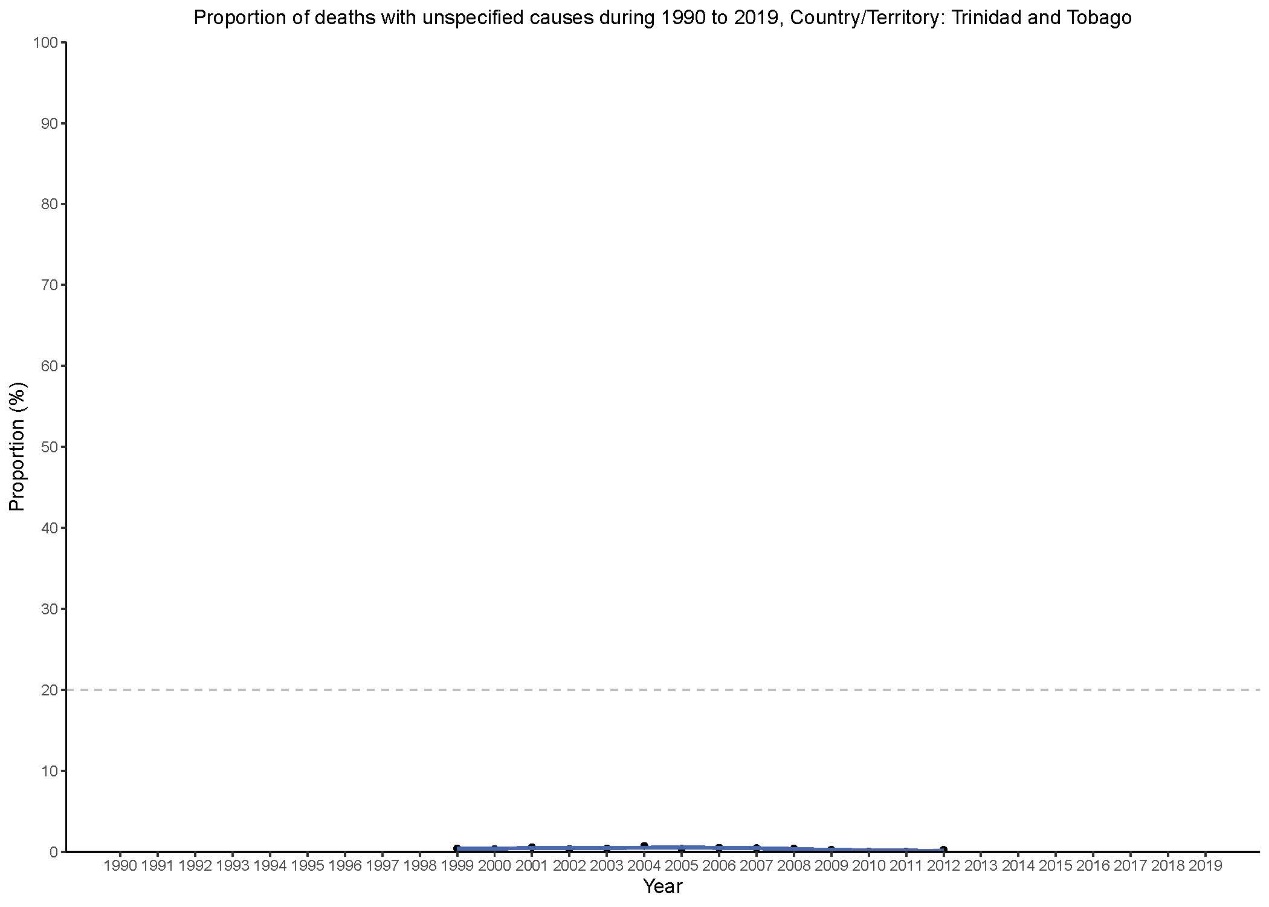

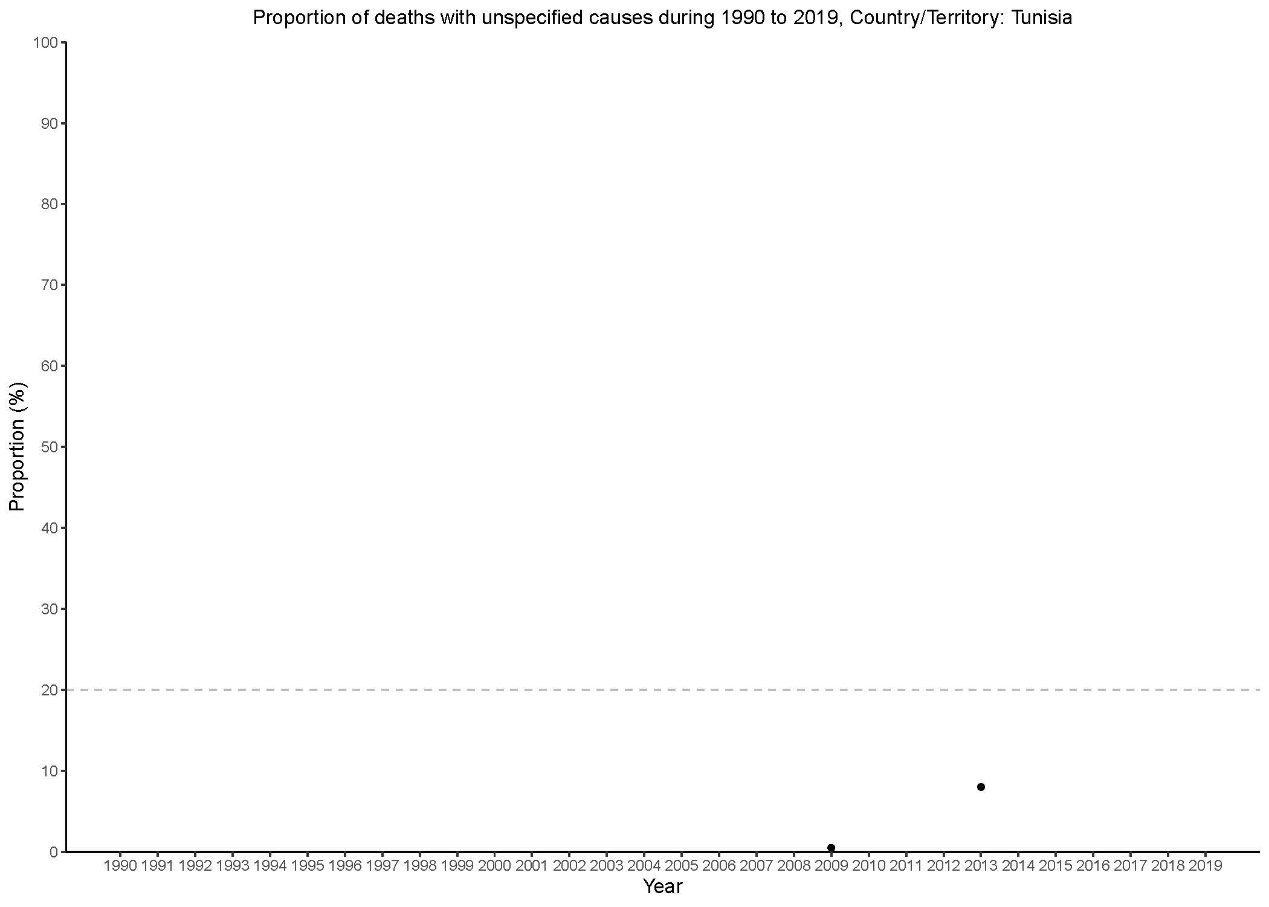

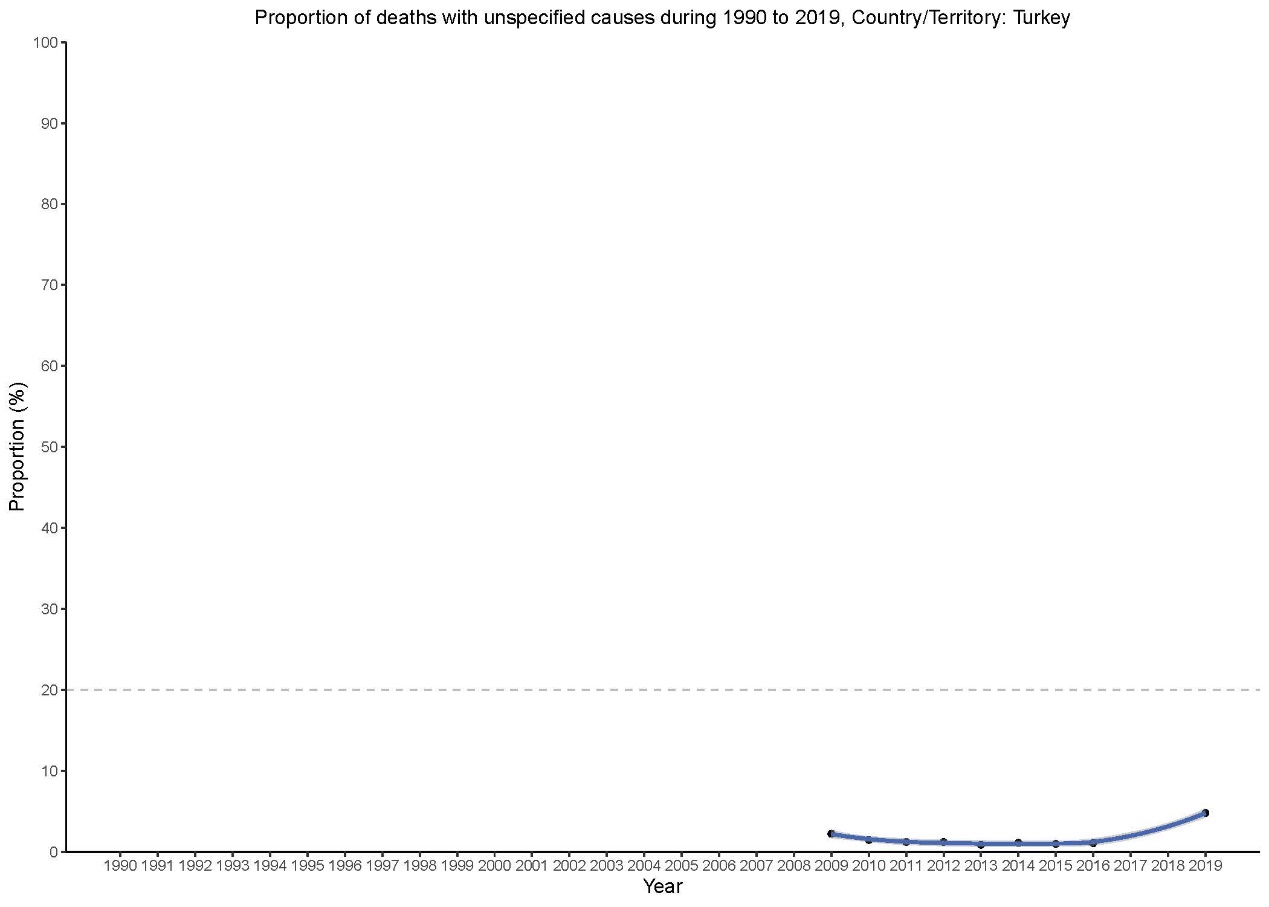

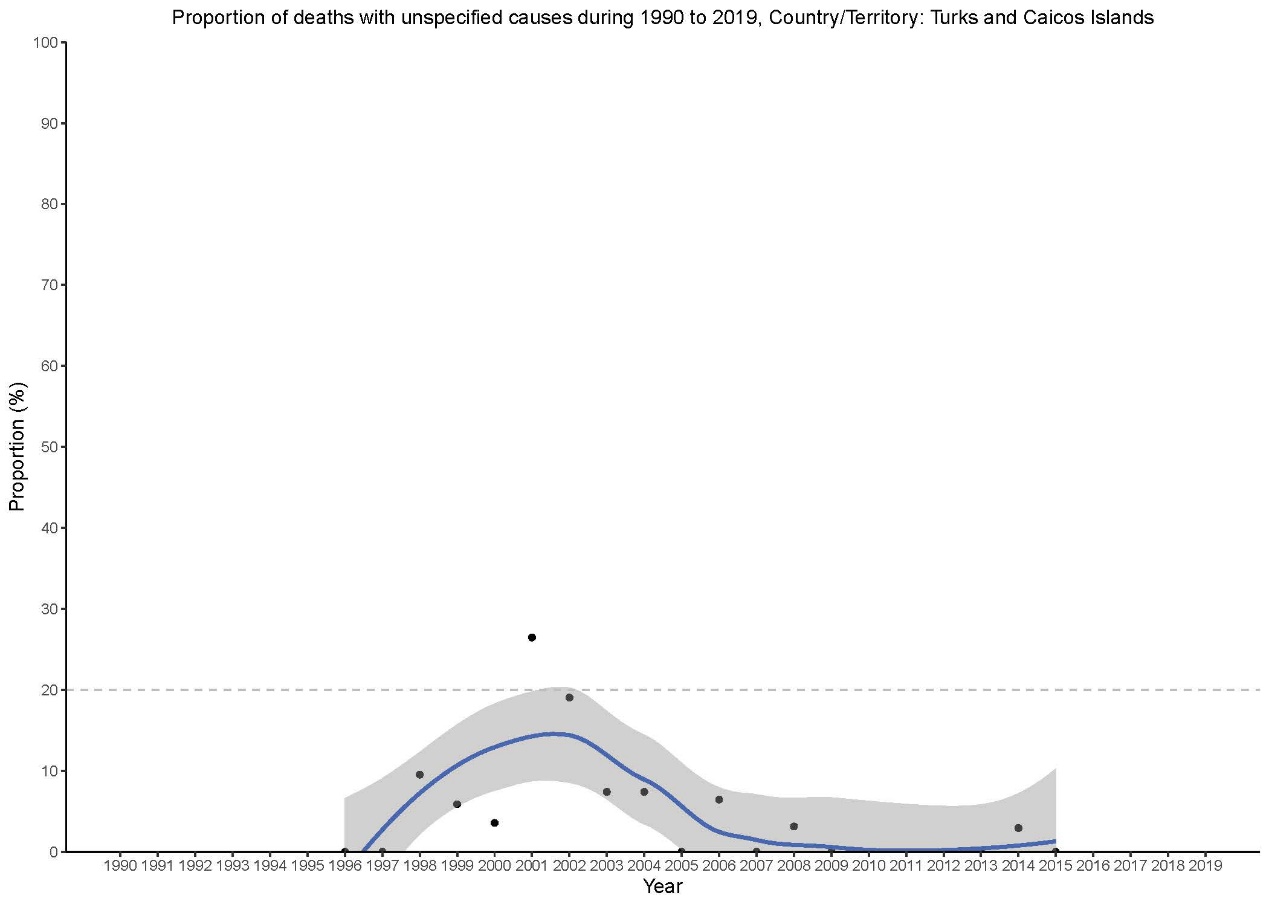

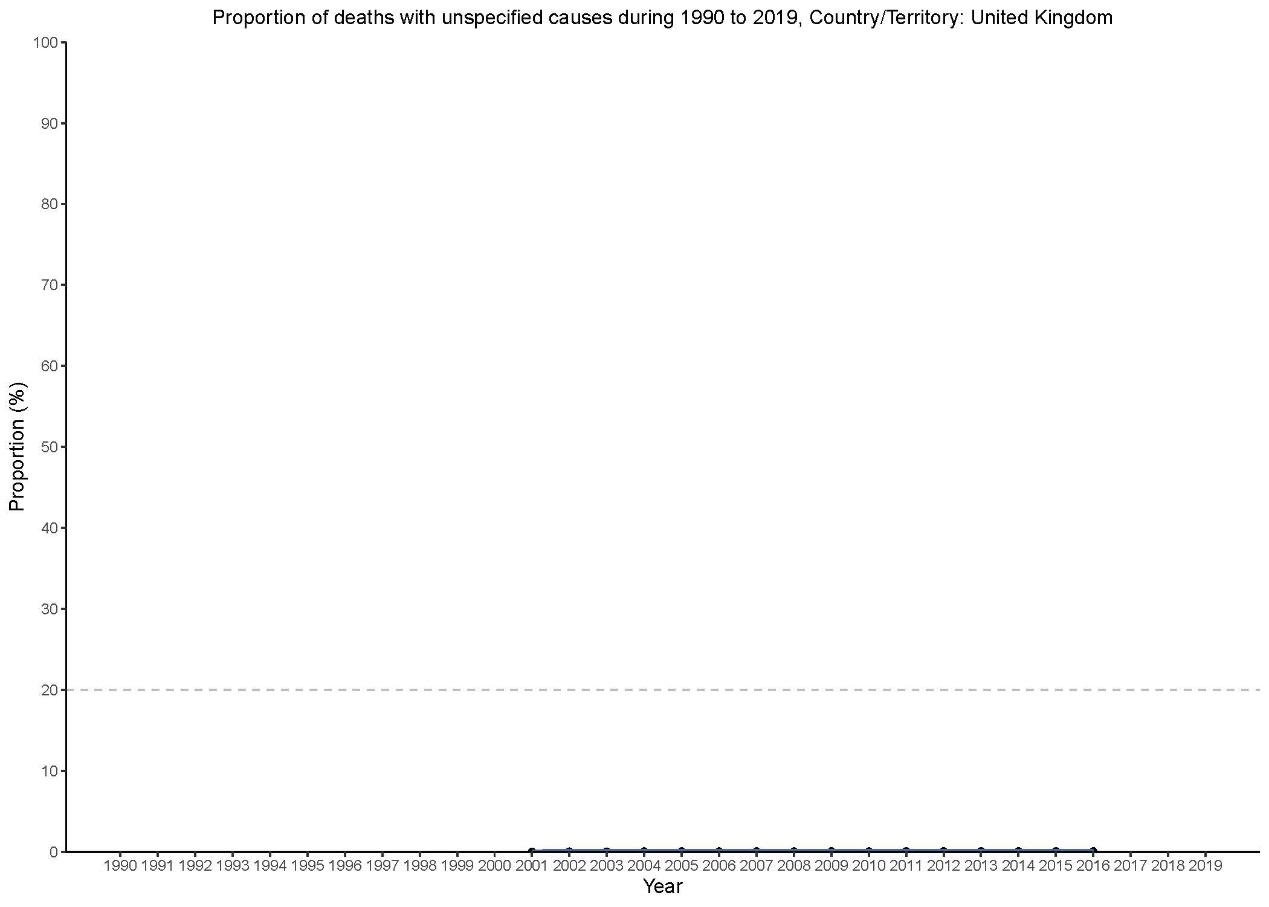

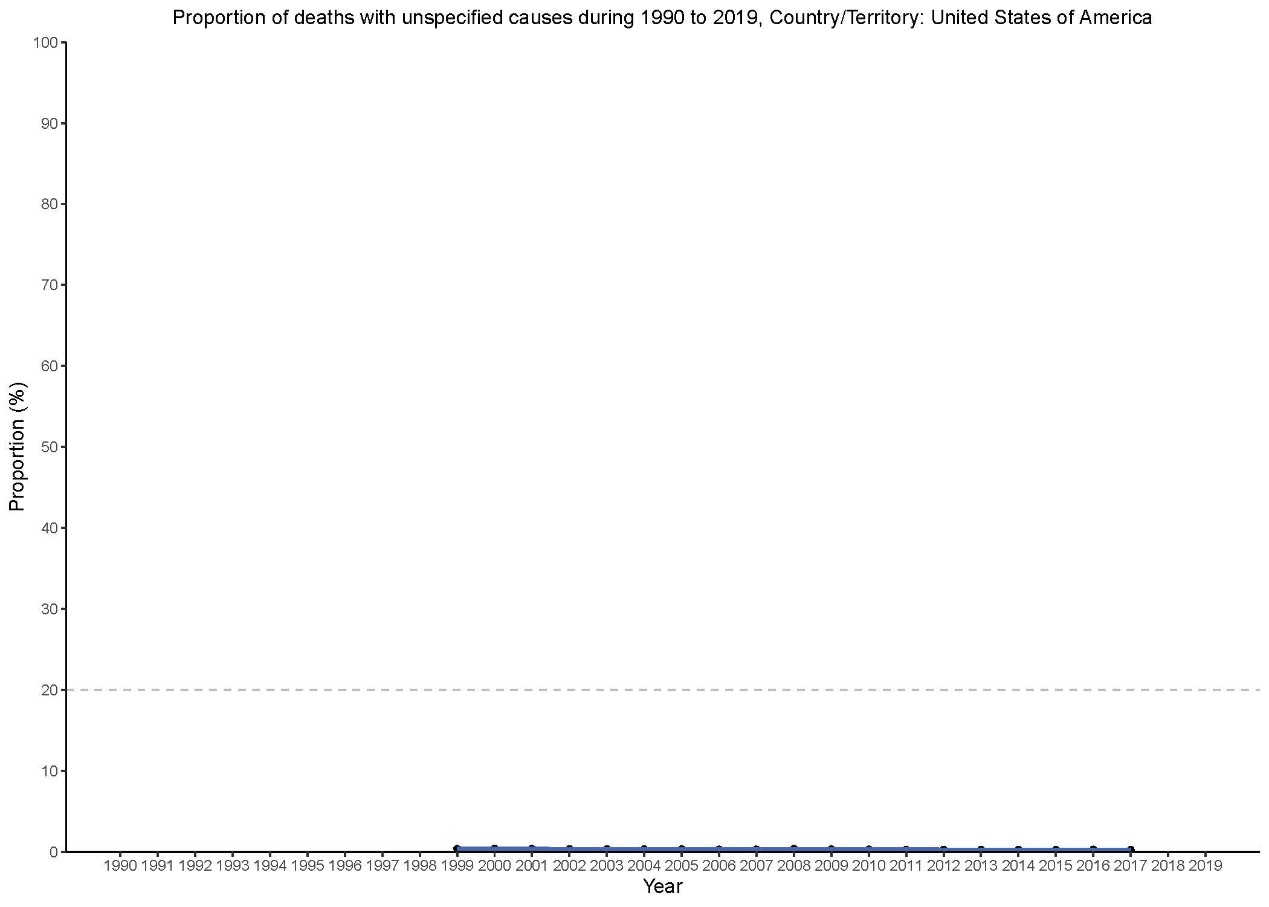

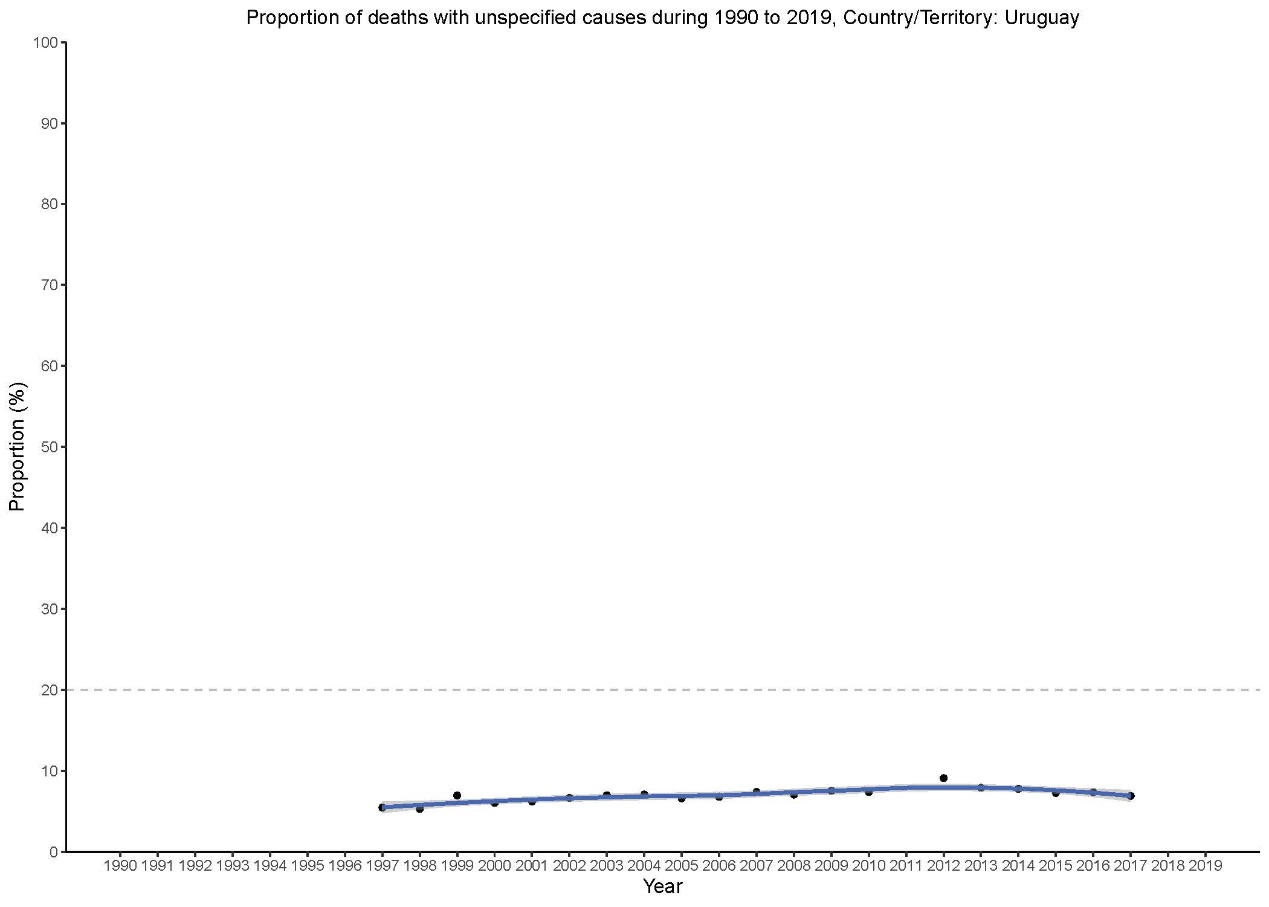
**

**Appendix 3. Crude and corrected age-adjusted unintentional fall mortality**

**Appendix 4. Flow chart of selection of eligible countries/territories**

**Appendix 5. GATHER checklist**

| Item # | Checklist item | Reported on page # |
| --- | --- | --- |
| Objectives and funding | | |
| 1 | Define the indicator(s), populations (including age, sex, and geographic entities), and time period(s) for which estimates were made. | Main text (Methods-Data source-paragraph 1-2, Quality measures of mortality data-paragraph 2-3), page 2-3 |
| 2 | List the funding sources for the work. | Main text (Funding-paragraph 1), page 11 |
| Data Inputs | | |
| *For all data inputs from multiple sources that are synthesized as part of the study:* | | |
| 3 | Describe how the data were identified and how the data were accessed. | Main text (Methods-Data source- paragraph 1-2), page 2 |
| 4 | Specify the inclusion and exclusion criteria. Identify all ad-hoc exclusions. | Main text (Methods- Quality measures of mortality data- a. Data availability- paragraph 1, Statistical analysis-paragraph 1), page 2-3 and appendix 4 (flowchart), page 300 |
| 5 | Provide information on all included data sources and their main characteristics. For each data source used, report reference information or contact name/institution, population represented, data collection method, year(s) of data collection, sex and age range, diagnostic criteria or measurement method, and sample size, as relevant. | Main text (Methods- Data Source- paragraph 1-2, Results-Data availability- paragraph 1), page 2-4 |
| 6 | Identify and describe any categories of input data that have potentially important biases (e.g., based on characteristics listed in item 5). | Main text (Discussion- Study limitations- paragraph 1), page 10 |
| *For data inputs that contribute to the analysis but were not synthesized as part of the study:* | | |
| 7 | Describe and give sources for any other data inputs. | All data were derived from the WHO Mortality Database |
| *For all data inputs:* | | |
| 8 | Provide all data inputs in a file format from which data can be efficiently extracted (e.g., a spreadsheet rather than a PDF), including all relevant meta-data listed in item 5. For any data inputs that cannot be shared because of ethical or legal reasons, such as third-party ownership, provide a contact name or the name of the institution that retains the right to the data. | Available from: https://www.who.int/data/data-collection-tools/who-mortality-database  and https://population.un.org/wpp/Download/Standard/Population/ |
| Data analysis | | |
| 9 | Provide a conceptual overview of the data analysis method. A diagram may be helpful. | Main text (Methods- Statistical analysis- paragraph 2-4), page 3-4 |
| 10 | Provide a detailed description of all steps of the analysis, including mathematical formulae. This description should cover, as relevant, data cleaning, data pre-processing, data adjustments and weighting of data sources, and mathematical or statistical model(s). | Main text (Methods- Quality measures of mortality data- paragraph 2-3, Models correcting problematic codes- paragraph 1, Statistical analysis- paragraph 2-4), page 2-4 |
| 11 | Describe how candidate models were evaluated and how the final model(s) were selected. | No candidate models were evaluated |
| 12 | Provide the results of an evaluation of model performance, if done, as well as the results of any relevant sensitivity analysis. | No relevant content |
| 13 | Describe methods for calculating uncertainty of the estimates. State which sources of uncertainty were, and were not, accounted for in the uncertainty analysis. | No relevant content |
| 14 | State how analytic or statistical source code used to generate estimates can be accessed. | Contact with corresponding author |
| Results and Discussion | | |
| 15 | Provide published estimates in a file format from which data can be efficiently extracted. | Appendix 1., Appendix 2.,  Appendix 3., page 3-299 |
| 16 | Report a quantitative measure of the uncertainty of the estimates (e.g. uncertainty intervals). | Appendix 2.,  Appendix 3., page 4-299 |
| 17 | Interpret results in light of existing evidence. If updating a previous set of estimates, describe the reasons for changes in estimates. | Main text (Discussion- Interpretation of findings- paragraph 1-8), page 7-10 |
| 18 | Discuss limitations of the estimates. Include a discussion of any modelling assumptions or data limitations that affect interpretation of the estimates. | Main text (Discussion- Study limitations- paragraph 1-2), page 10 |

*This checklist should be used in conjunction with the GATHER statement and Explanation and Elaboration document, found on gather-statement.org*
